# Supplementary material for: Structure-guided screening identifies Tucatinib as dual inhibitor for MCT1/2
Source: EMBO Rep. 2025 Dec 11;27(3):677–703. doi: 10.1038/s44319-025-00661-9 (PMC12894981; doi:10.1038/s44319-025-00661-9)
Supplement: Supplementary file 1 — Appendix [file 44319_2025_661_MOESM1_ESM.pdf]

Appendix

Structure-guided screening identifies Tucatinib as dual inhibitor for MCT1/2

Binghong Xu[1,#,*], Xiaoyu Zhou[1,#], Yuanyue Shan[2,#], Sai Shi[3,1,#], Jiachen, Li[1,#], Qinqin Liang[1], Ziyu, Wang[1], Mingfeng Zhang[4,*], Yaxin Wang[1,*], Duanqing Pei[4,*], Sheng Ye[1,*]

*Corresponding authors:
Sheng Ye, sye@tju.edu.cn; Duanqing Pei, peiduanqing@westlake.edu.cn; Yaxin Wang, wangyaxin@tju.edu.cn; Mingfeng Zhang, zhangmingfeng@westlake.edu.cn; Binghong Xu, binghong_xu@tju.edu.cn

List of Appendix Figures and Tables

Appendix Figures

Appendix Figure S1………………..page 2
Appendix Figure S2………………..page 4
Appendix Figure S3………………..page 6
Appendix Figure S4………………..page 7
Appendix Figure S5………………..page 8
Appendix Figure S6………………..page 10
Appendix Figure S7………………..page 11
Appendix Figure S8………………..page 12
Appendix Figure S9………………..page 13
Appendix Figure S10………………page 14
Appendix Figure S11………………page 15

Appendix Tables

Appendix Table S1…………………page 16
Appendix Table S2…………………page 17

Appendix Figures

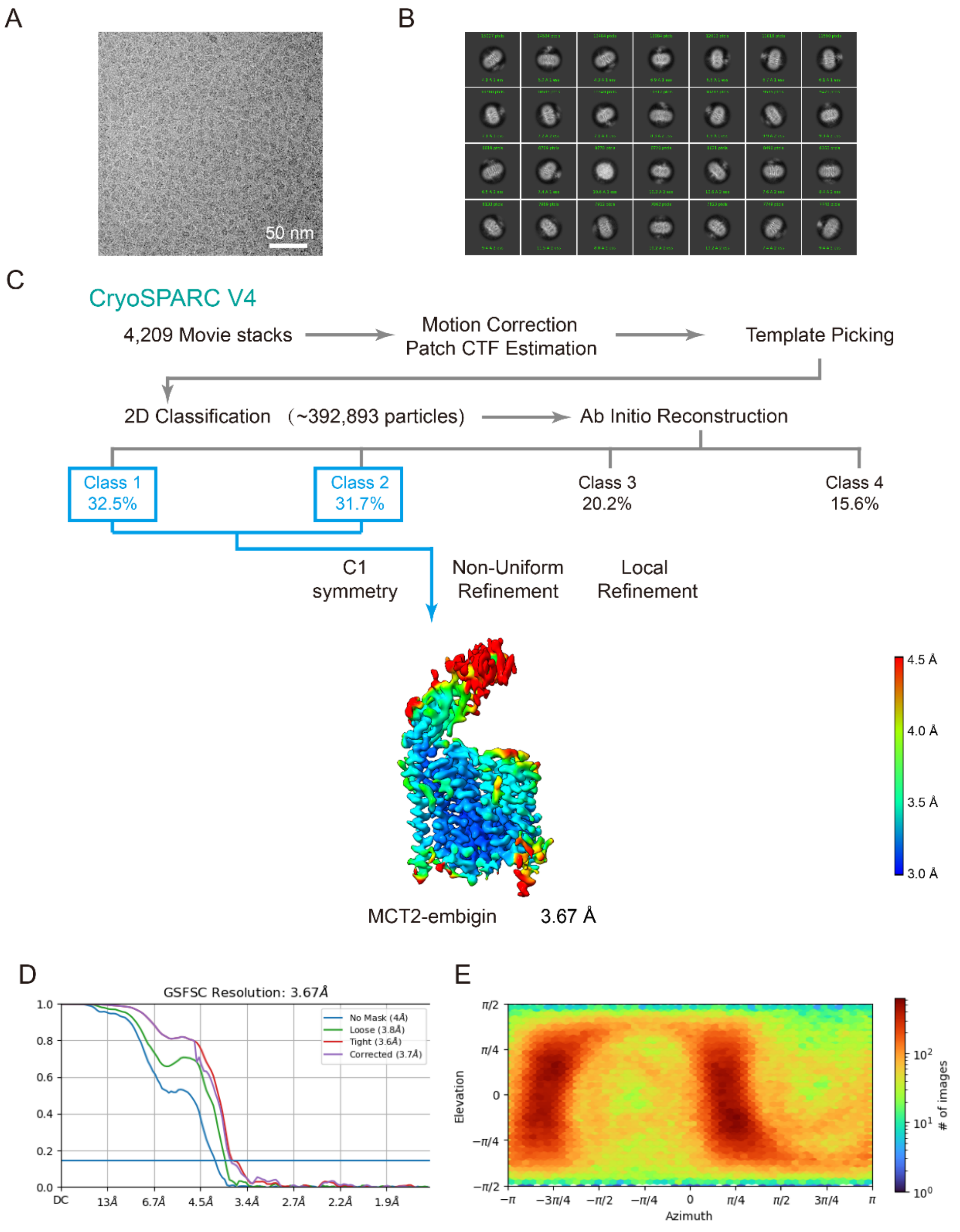

Appendix Figure S1. Structure determination of MCT2-emb, related to Fig. 1, Appendix Table S1.

(A) Representative cryo-EM micrograph of MCT2-emb. Scale bar, 50 nm.

(B) Representative 2D classification averages of MCT2-emb.

(C) Flowchart of image processing for MCT2-emb particles.

(D) Gold standard Fourier shell correlation (FSC) curve for the 3D refinement of the overall structure of the MCT2-emb.

(E) Angular distribution of the particles used for the final reconstructions.

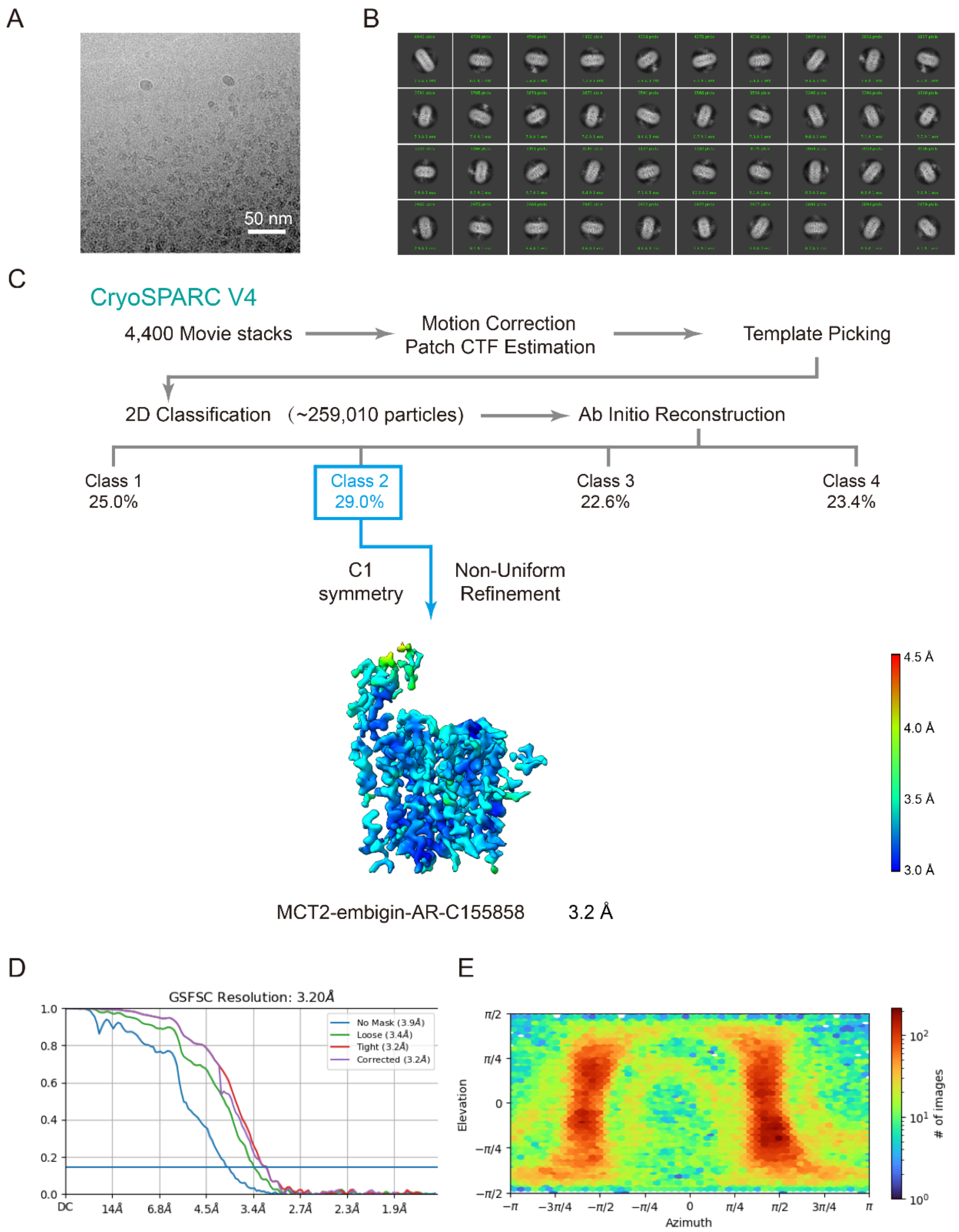

Appendix Figure S2. Structure determination of MCT2R-emb, related to Fig. 2, Appendix Table S1.

(A) Representative cryo-EM micrograph of MCT2R-emb. Scale bar, 50 nm.

(B) Representative 2D classification averages of MCT2R-emb.

(C) Flowchart of image processing for MCT2R-emb particles.

(D) Gold standard Fourier shell correlation (FSC) curve for the 3D refinement of the overall structure of the MCT2R-emb.

(E) Angular distribution of the particles used for the final reconstructions.

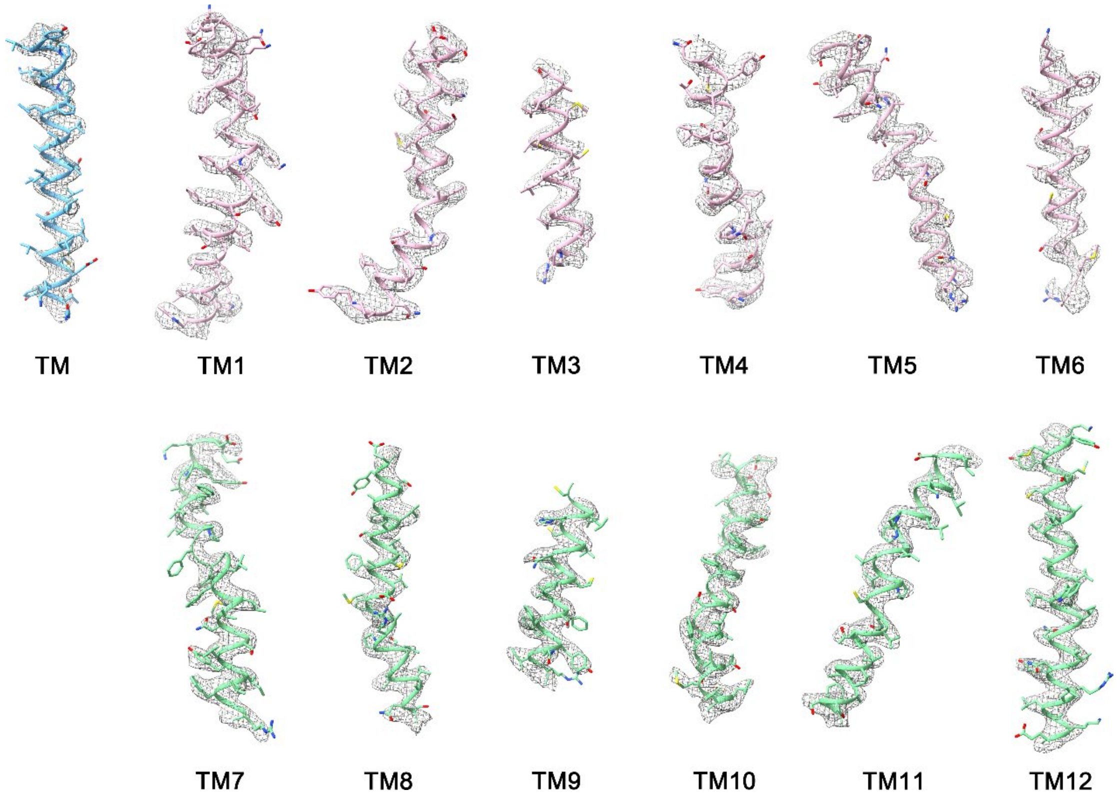

Appendix Figure S3. EM density maps of MCT2-emb, related to Fig. 1.

Sample maps at transmembrane helice of embigin, 12 transmembrane helices of MCT2.

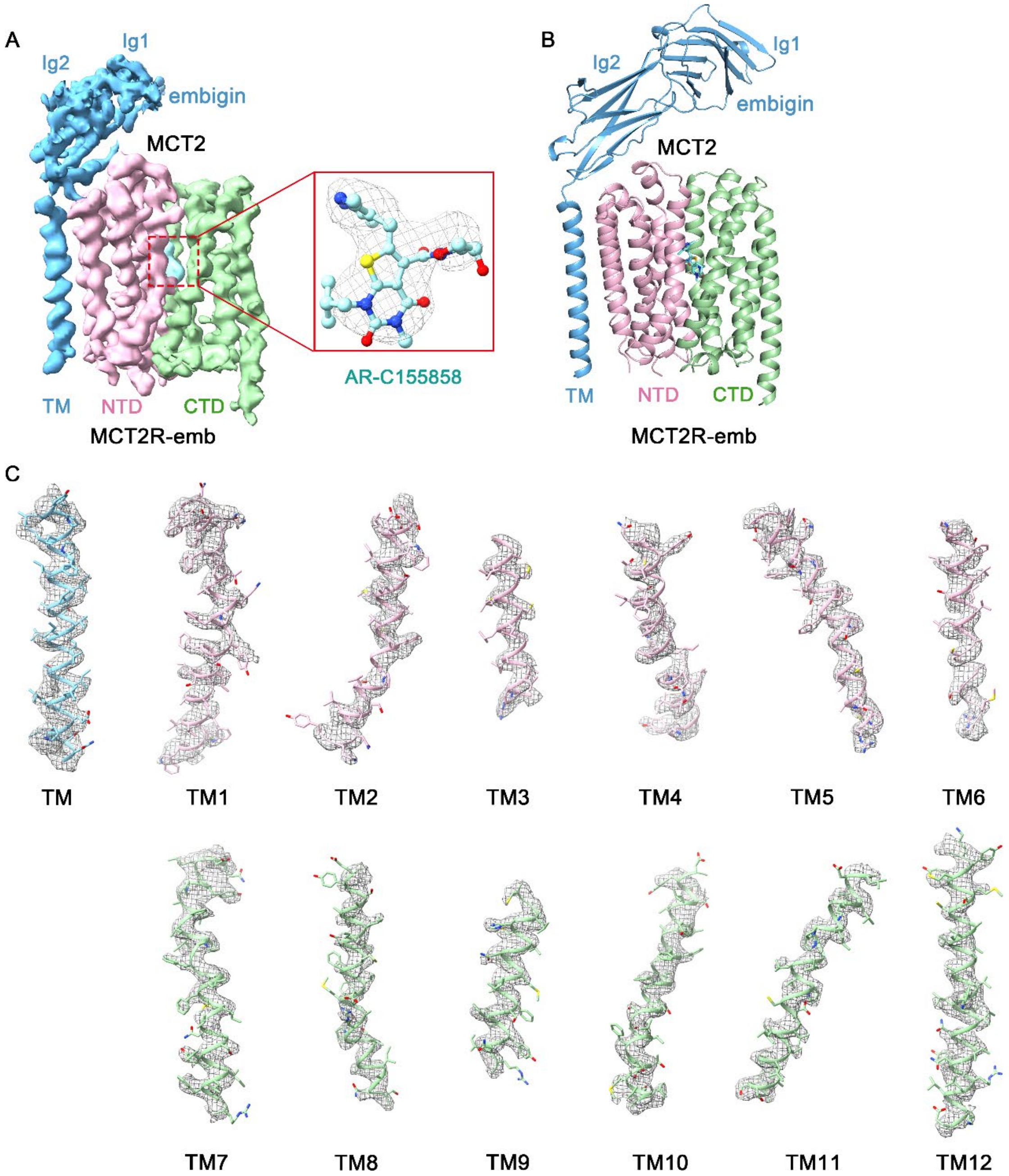

Appendix Figure S4. Overall structure and EM density maps of MCT2R-emb, related to Fig. 2.

(A) 3D EM reconstruction of MCT2R-emb (left); The cryo-EM density of AR-C155858 (right).

(B) Overall structure of MCT2R-emb at outward-open conformation. The colors of the structural domains are corresponding to those in MCT2-emb, AR-C155858 (cyan).

(C) Sample maps at transmembrane helice of embigin, 12 transmembrane helices of MCT2.

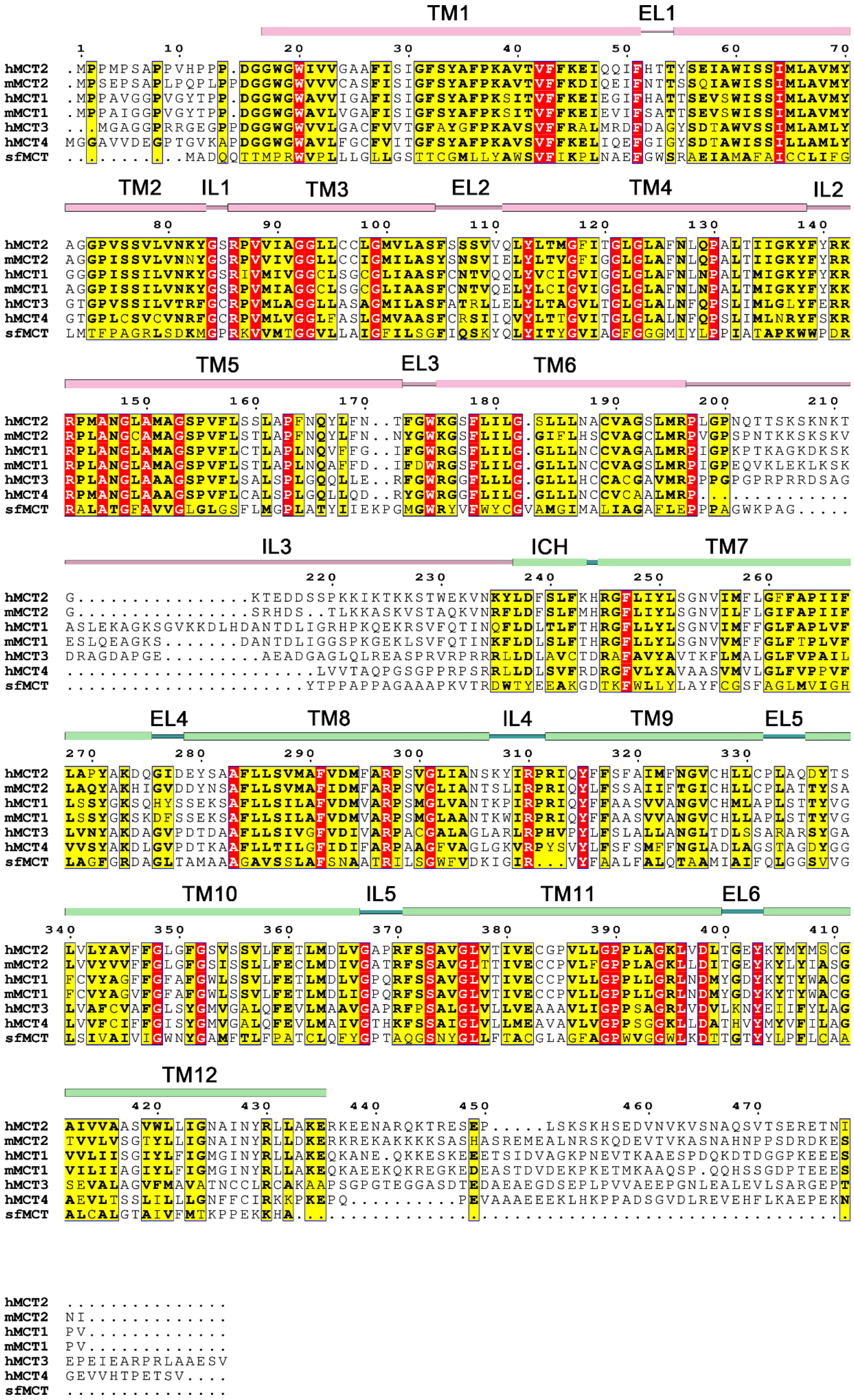

Appendix Figure S5. Sequence alignment of human MCT1-4, mouse MCT1-2 and

sfMCT, related to Figs. 1-3.

Secondary structure of assignments is based on the human MCT2-embigin structure. Invariant and highly conserved residues are shaded red and yellow, respectively.

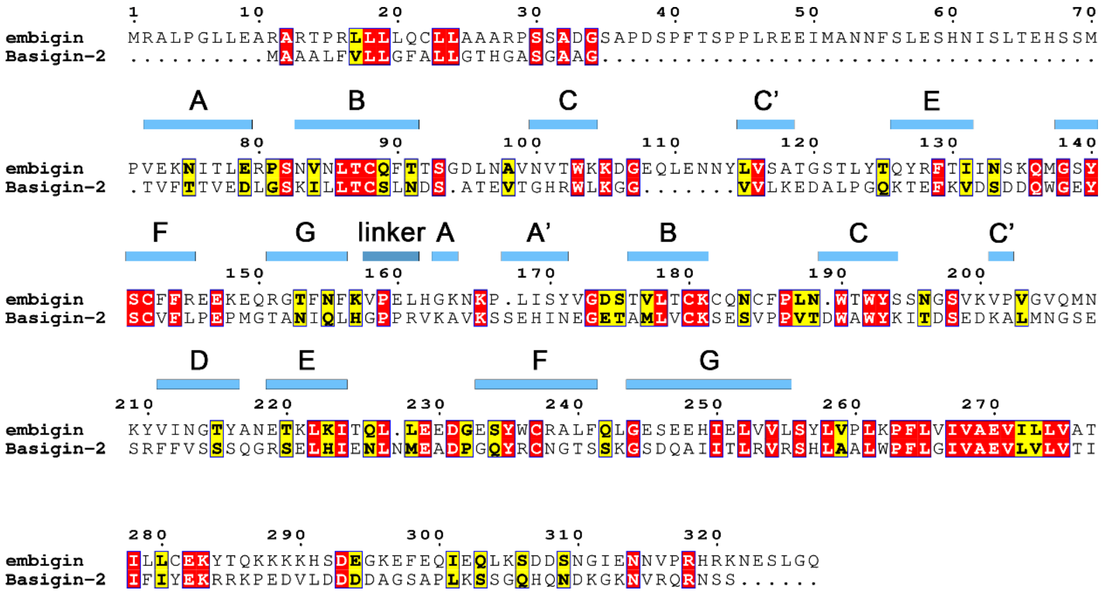

Appendix Figure S6. Sequence alignment of human Basigin-2 with embigin, related to Figs. 1-3.

Secondary structure of assignments is based on the human MCT2-embigin structure. Invariant and highly conserved residues are shaded red and yellow, respectively.

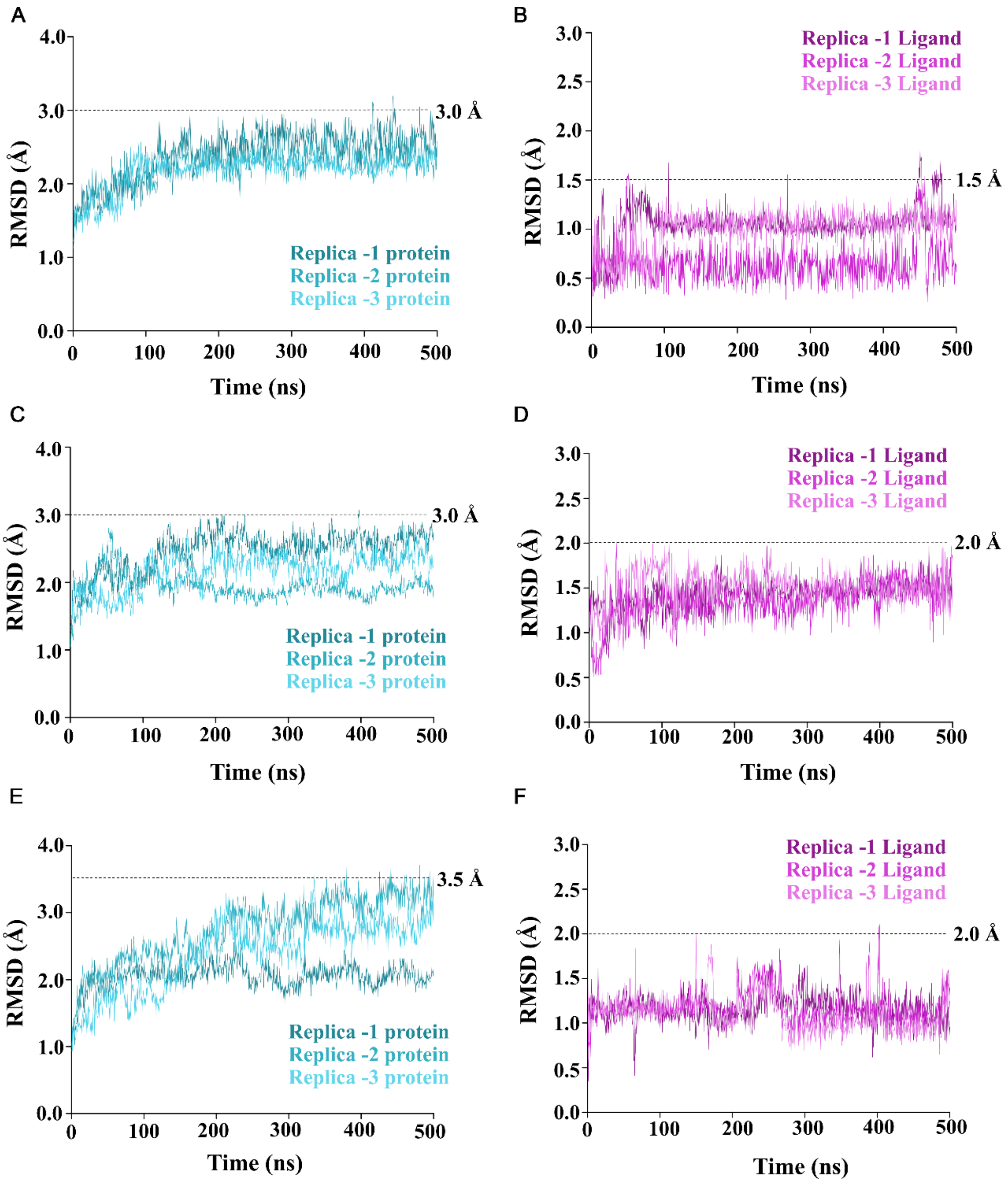

Appendix Figure S7. MD between MCTs and inhibitors, related to Figs. 2 and 4.

AR-C155858 with MCT2 simulated system in protein backbone RMSD (A), ligand heavy atom RMSD (B).

RMSD of protein backbone Cα atom in MCT1 vs. Tucatinib simulation system (C) and RMSD of Tucatinib heavy atom (D).

RMSD of MCT2 with the Cα atom of the main chain of the Tucatinib mimetic system protein (E) and the RMSD of the Tucatinib heavy atom (F).

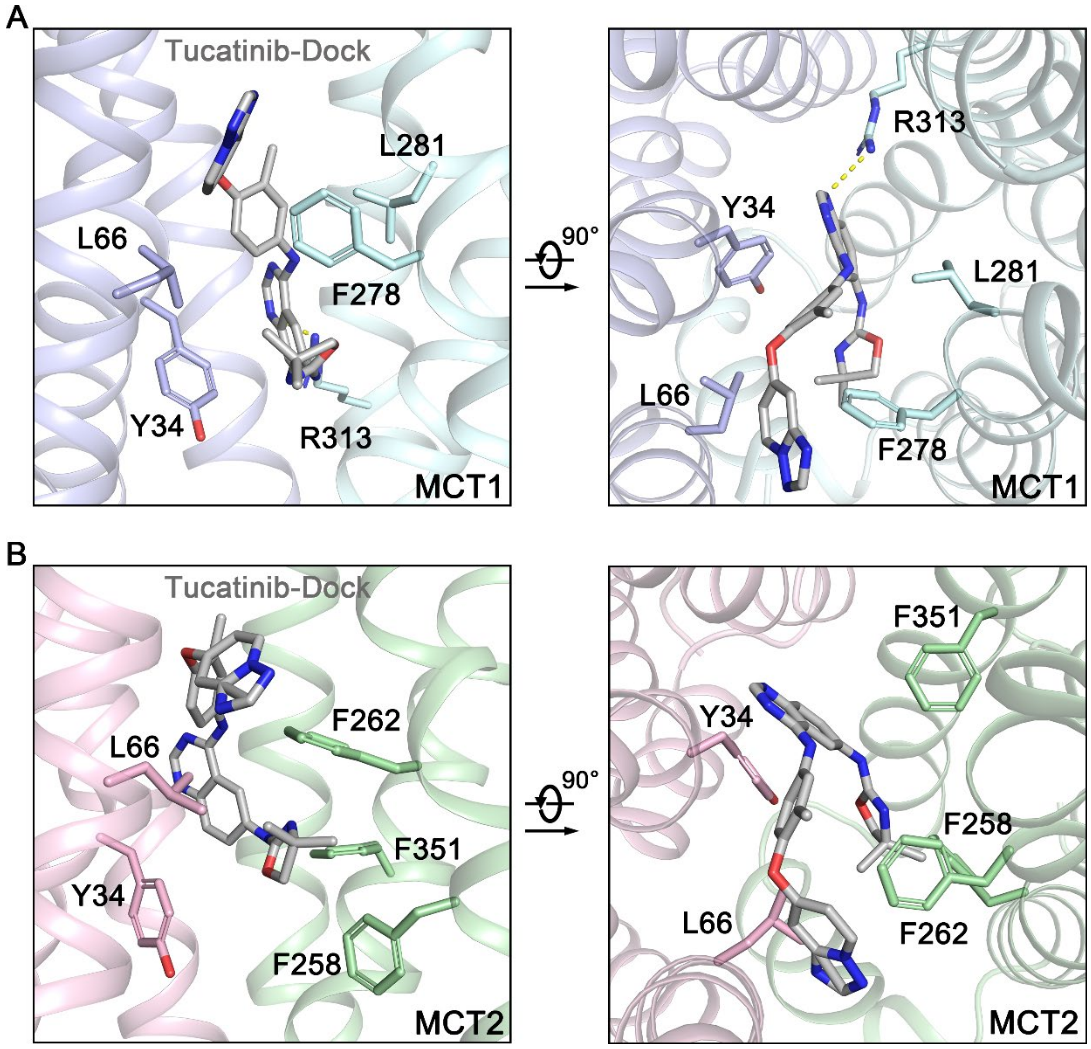

Appendix Figure S8. **Binding modes of Tucatinib with MCTs, related to Fig. 4.**

(A) Coordination of Tucatinib by MCT1 in Tucatinib-MCT1 docking model. Both hydrophobic residues and hydrophilic residues are involved in inhibitor recognition.

(B) Coordination of Tucatinib by MCT2 in Tucatinib-MCT2 docking model. Major hydrophobic residues involved in inhibitor recognition are shown as sticks.

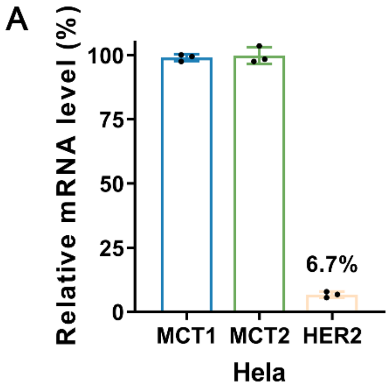

Appendix Figure S9. Relative mRNA level of MCT1, MCT2 and HER2 in Hela cells, related to Fig. 5.

Relative mRNA level of MCT1, MCT2 and HER2 in Hela cells.

The graphical presentation and data analysis were conducted using GraphPad Prism 9.

The data are displayed as mean ± standard deviation (S.D.). n = 3 biological replicates.

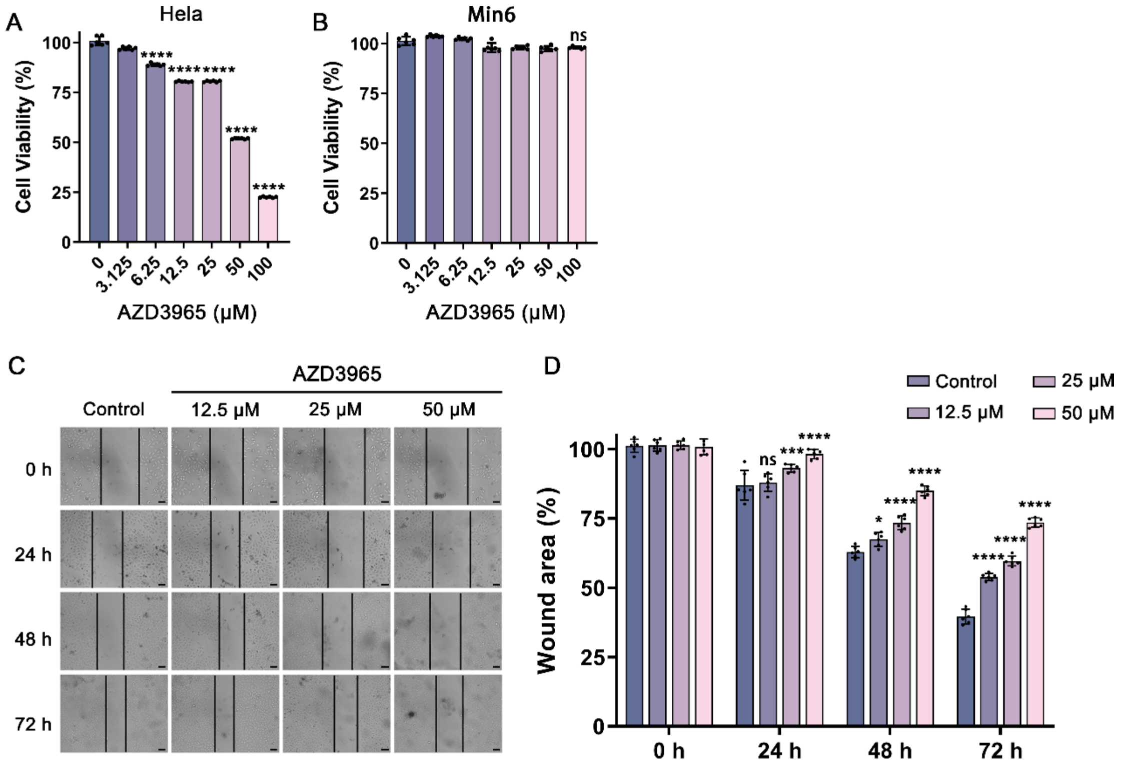

Appendix Figure S10. AZD3965 inhibits the proliferation and migration of Hela cells by targeting MCT1/2, related to Figs. 5 and 6.

Inhibitory effect of AZD3965 on the proliferation of Hela (A), Min6 (B). n = 6 biological replicates.

(C) Inhibitory effect of the migration of Hela cells with different concentrations of AZD3965. Scale bar, 100 µm.

(D) Statistical results of (C). n = 6 biological replicates. *: *p*=0.0107

The graphical presentation and data analysis were conducted using GraphPad Prism 9. The data are displayed as mean ± standard deviation (S.D.). Statistical significance of the differences between group means was evaluated by one-way analysis of variance (ANOVA) using the Tukey honestly significant difference test as a post hoc test; p values≤0.05 were considered statistically significant (*, $p < 0.05$; **, $p < 0.01$; ***, $p < 0.001$; ****, $p < 0.0001$, ns, not significant).

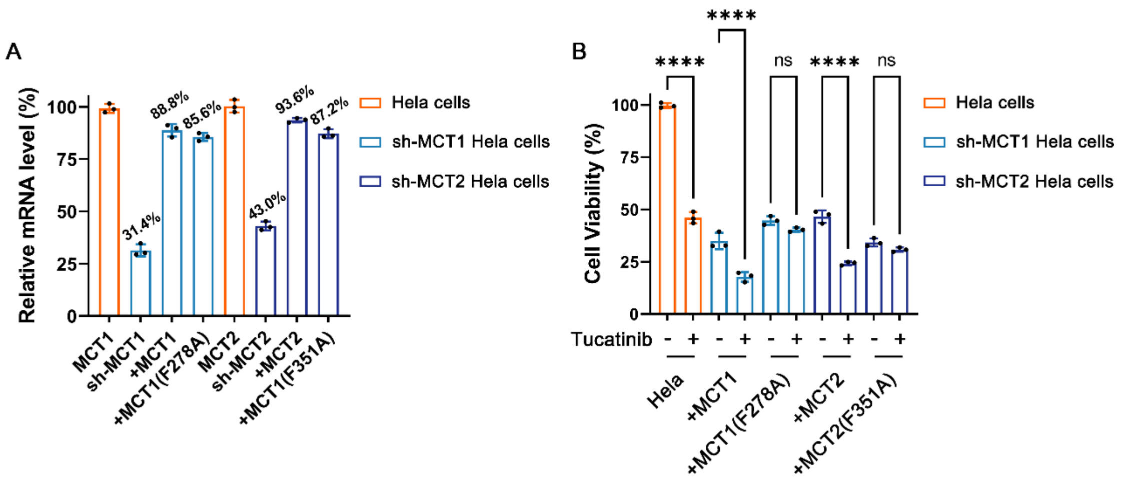

Appendix Figure S11. Tucatinib inhibits the propagation of Hela cells by targeting MCT1/2, related to Fig. 5.

(A) Relative mRNA level of the rescue MCT1 (WT or F278A) or MCT2 (WT or F351A) in corresponding knockdown Hela cells using qPCR. n = 3 biological replicates.

(B) The cell viability of Hela cells after rescue MCT1 (WT or F278A) or MCT2 (WT or F351A) in corresponding knockdown cell lines treated by Tucatinib (12.5 μM). n = 3 biological replicates.

The graphical presentation and data analysis were conducted using GraphPad Prism 9. The data are displayed as mean ± standard deviation (S.D.). Statistical significance of the differences between group means was evaluated by one-way analysis of variance (ANOVA) using the Tukey honestly significant difference test as a post hoc test; p values≤0.05 were considered statistically significant (*, $p < 0.05$; **, $p < 0.01$; ***, $p < 0.001$; ****, $p < 0.0001$, ns, not significant).

Appendix Tables

Appendix Table S1. Data collection and refinement statistics.

**Data collection and processing**	**MCT2-emb**	**MCT2R-emb**
Magnification	105K	105K
Voltage (kV)	300	300
Electron exposure (e–/Å^2)	60	60
Defocus range (μm)	-1.5 to -2.0	-1.5 to -2.0
Pixel size (Å)	0.839	0.849
Symmetry imposed	C1	C1
Total number of movies	4209	4400
Initial particle images	1,196,214	700,440
Final particle images	305,119	76,628
Map resolution (Å)	3.7	3.2
FSC threshold	0.143	0.143
**Refinement**		
Model resolution (Å)	3.7	3.2
Map sharpening B factor (Å^2)	138.6	115.8
Model composition		
Non-hydrogen atoms	4,826	4,876
Protein residues	619	621
Ligands	0	1
r.m.s. deviation		
Bond lengths (Å)	0.003	0.004
Bond angles (°)	0.749	0.728
Validation		
MolProbity score	1.73	1.83
Clashscore	9.18	11.26
Rotamer outliers (%)	0.00	0.00
Ramachandran plot		
Favored (%)	96.41	96.10
Allowed (%)	3.59	3.90
Outliers (%)	0.00	0.00

Appendix Table S2. Primers used in the study.

MCT2	Sequences (5'-3')
MCT2-F	GGTCCGAAGCGCGCGGAATTCATGCCACCAATGCCAAGT
MCT2-R	GCGACTAGTGAGCTCGTCGACAATGTTAGTTTCTCTTTC
R86A-F	AATAAATACGGCAGCGCGCCGGTGGTGATAGCA
R86A-R	TGCTATCACCACCGGCGCGCTGCCGTATTTATT
R86E-F	AATAAATACGGCAGCGAGCCGGTGGTGATAGCA
R86E-R	TGCTATCACCACCGGCTCGCTGCCGTATTTATT
K175A-F	AATACTTTTGGCTGGGCAGGAAGCTTCCTGATT
K175A-R	AATCAGGAAGCTTCCTGCCCAGCCAAAAGTATT
K175D-F	AATACTTTTGGCTGGGATGGAAGCTTCCTGATT
K175D-R	AATCAGGAAGCTTCCATCCCAGCCAAAAGTATT
N187A-F	GGAAGTCTACTTTTGGCTGCCTGTGTGGCTGGT
N187A-R	ACCAGCCACACAGGCAGCCAAAAGTAGACTTCC
N187E-F	GGAAGTCTACTTTTGGAAGCCTGTGTGGCTGGT
N187E-R	ACCAGCCACACAGGCTTCCAAAAGTAGACTTCC
F172D-F	TACCTTTTTAATACTGATGGCTGGAAAGGAAGC
F172D-R	GCTTCCTTTCCAGCCATCAGTATTAAAAAGGTA
G176D-F	ACTTTTGGCTGGAAAGACAGCTTCCTGATTTTG
G176D-R	CAAAATCAGGAAGCTGTCTTTCCAGCCAAAAGT
I180D-F	AAAGGAAGCTTCCTGGATTTGGGAAGTCTACTT
I180D-R	AAGTAGACTTCCCAAATCCAGGAAGCTTCCTTT

S183D-F	TTCCTGATTTTGGGAGATCTACTTTTGAATGCC
S183D-R	GGCATTCAAAAGTAGATCTCCCAAAATCAGGAA
L186D-F	TTGGGAAGTCTACTTGATAATGCCTGTGTGGCT
L186D-R	AGCCACACAGGCATTATCAAGTAGACTTCCCAA
V190D-F	CTTTTGAATGCCTGTGATGCTGGTTCCCTCATG
V190D-R	CATGAGGGAACCAGCATCACAGGCATTCAAAAG
S193D-F	TGTGTGGCTGGTGACCTCATGAGACCC
S193D-R	GGGTCTCATGAGGTCACCAGCCACACA
L194D-F	TGTGTGGCTGGTTCCGACATGAGACCCCTTGGA
L194D-R	TCCAAGGGGTCTCATGTCGGAACCAGCCACACA
Y34A-F	TCCATTGGATTTTCCGCTGCATTCCCCAAAGCT
Y34A-R	AGCTTTGGGGAATGCAGCGGAAAATCCAATGGA
F262A-F	ATGTTCCTAGGTTTTGCTGCCCCCATTATATTC
F262A-R	GAATATAATGGGGGCAGCAAAACCTAGGAACAT
D293A-F	GTTATGGCTTTCGTTGCTATGTTTGCTAGGCCT
D293A-R	AGGCCTAGCAAACATAGCAACGAAAGCCATAAC
R297A-F	GTTGATATGTTTGCTGCGCCTTCTGTAGGATTA
R297A-R	TAATCCTACAGAAGGCGCAGCAAACATATCAAC
F351A-F	TTTTTTGGCCTTGGAGCTGGGAGTGTTAGCAGT
F351A-R	ACTGCTAACACTCCCAGCTCCAAGGCCAAAAAA

**Embigin**	**Sequences (5'-3')**
Embigin-F	GGTCCGAAGCGCGCGGAATTCATGCGTGCTCTGCCTGGTTTG

Embigin-R	GCGACTAGTGAGCTCGTCGACCTGACCCAGGCTTTCGTT
Y171A-F	AAACCTCTGATTAGCGCTGTGGGCGATAGCACC
Y171A-R	GGTGCTATCGCCCACAGCGCTAATCAGAGGTTT
Y171R-F	AAACCTCTGATTAGCCGTGTGGGCGATAGCACC
Y171R-R	GGTGCTATCGCCCACACGGCTAATCAGAGGTTT
D174A-F	CTGATTAGCTATGTGGGCGCTAGCACCGTGTTGACCTGC
D174A-R	GCAGGTCAACACGGTGCTAGCGCCCACATAGCTAATCAG
D174K-F	ATTAGCTATGTGGGCAAGAGCACCGTGTTGACC
D174K-R	GGTCAACACGGTGCTCTTGCCCACATAGCTAAT
Y257A-F	GAACTGGTGGTGCTGTCCGCTCTGGTGCCTCTGAAACCT
Y257A-R	AGGTTTCAGAGGCACCAGAGCGGACAGCACCACCAGTTC
Y257R-F	GAACTGGTGGTGCTGTCCCGTCTGGTGCCTCTGAAACCT
Y257R-R	AGGTTTCAGAGGCACCAGACGGGACAGCACCACCAGTTC
E270A-F	TTTCTGGTGATTGTGGCCGCGGTGATTTTGCTGGTGGCC
E270A-R	GGCCACCAGCAAAATCACCGCGGCCACAATCACCAGAAA
E270R-F	TTTCTGGTGATTGTGGCCCGGGTGATTTTGCTGGTGGCC
E270R-R	GGCCACCAGCAAAATCACCCGGGCCACAATCACCAGAAA
E282A-F	GCCACCATTCTGCTGTGCGCGAAATATACCCAGAAGAAG
E282A-R	CTTCTTCTGGGTATATTTCGCGCACAGCAGAATGGTGGC
E282R-F	GCCACCATTCTGCTGTGCCGGAAATATACCCAGAAGAAG
E282R-R	CTTCTTCTGGGTATATTTCCGGCACAGCAGAATGGTGGC
I169D-F	AAAAACAAACCTCTGGATAGCTATGTGGGCGAT

I169D-R	ATCGCCCACATAGCTATCCAGAGGTTTGTTTTT
P260D-F	CTGTCCTATCTGGTGGATCTGAAACCTTTTCTG
P260D-R	CAGAAAAGGTTTCAGATCCACCAGATAGGACAG
F264D-F	CTGGTGCCTCTGAAACCTGATCTGGTGATTGTGGCCGAG
F264D-R	CTCGGCCACAATCACCAGATCAGGTTTCAGAGGCACCAG
I267D-F	CTGAAACCTTTTCTGGTGGATGTGGCCGAGGTGATTTTG
I267D-R	CAAAATCACCTCGGCCACATCCACCAGAAAAGGTTTCAG
V268D-F	CCTTTTCTGGTGATTGACGCCGAGGTGATTTTG
V268D-R	CAAAATCACCTCGGCGTCAATCACCAGAAAAGG
V271D-F	CTATACCTCTCTGGAAATGACATCATGTTTTTGGACTC
V271D-R	GAGTCCAAAAACATGATGTCATTTCCAGAGAGGTATAG
L274D-F	GCCGAGGTGATTTTGGATGTGGCCACCATTCTG
L274D-R	CAGAATGGTGGCCACATCCAAAATCACCTCGGC
V275D-F	GCCGAGGTGATTTTGCTGGACGCCACCATTCTGCTGTGC
V275D-R	GCACAGCAGAATGGTGGCGTCCAGCAAAATCACCTCGGC
I278D-F	ATTTTGCTGGTGGCCACCGATCTGCTGTGCGAGAAATAT
I278D-R	ATATTTCTCGCACAGCAGATCGGTGGCCACCAGCAAAAT
L279D-F	CTGGTGGCCACCATTGATCTGTGCGAGAAATAT
L279D-R	ATATTTCTCGCACAGATCAATGGTGGCCACCAG

**MCT1**	**Sequences (5’-3’)**
MCT1-F	GGTCCGAAGCGCGCGGAATTCAAGCCAACCAAGGCAGGGAAA
MCT1-R	GCGACTAGTGAGCTCGTCGACGACTGGACTTTCCTCCTC

Y34A-F	TCCATCGGCTTCTCTGCTGCATTTCCCAAATCA
Y34A-R	TGATTTGGGAAATGCAGCAGAGAAGCCGATGGA
F278A-F	ATCATGTTTTTTGGACTCGCTGCACCTTTGGTGTTT
F278A-R	AAACACCAAAGGTGCAGCGAGTCCAAAAAACATGAT
R313A-F	GTTGACATGGTAGCCGCACCATCTATGGGACTT
R313A-R	GTTGACATGGTAGCCGCACCATCTATGGGACTT
**qPCR related**	**Sequences (5’-3’)**
MCT1-F	TTTCTTTGCGGCTTCCGTTG
MCT1-R	CTCTGGGGTCCAACAAGGTC
MCT2-F	GACACGTCAGGGGCCATAAAT
MCT2-R	GGTTCCCCAGATCACCTTGT
GAPDH-F	CCCACTCCTCCACCTTTGACG
GAPDH-R	CACCACCCTGTTGCTGTAGCCA
