# Supplementary figures and images for: Structure-guided screening identifies Tucatinib as dual inhibitor for MCT1/2
Source: EMBO Rep. 2025 Dec 11;27(3):677–703. doi: 10.1038/s44319-025-00661-9 (PMC12894981; doi:10.1038/s44319-025-00661-9)

**Fig. 1 G**

MCT2 (WT)

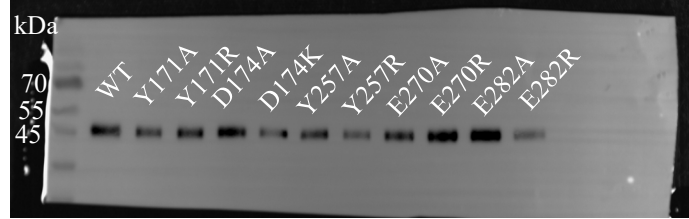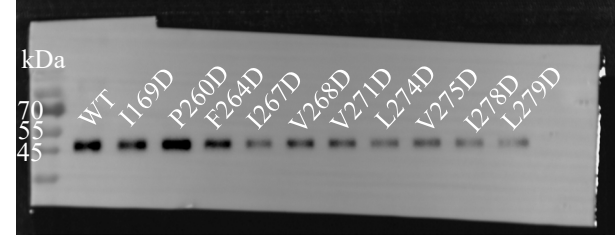

embigin (variants)

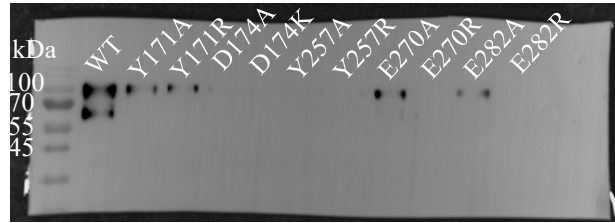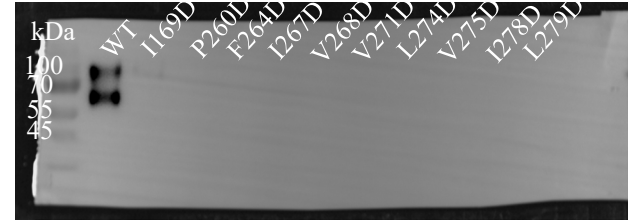

MCT2 (WT)

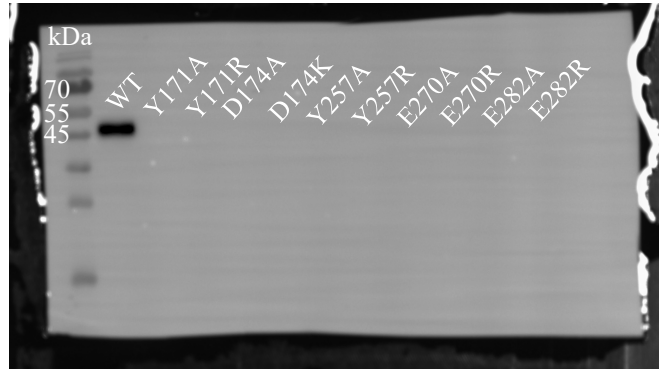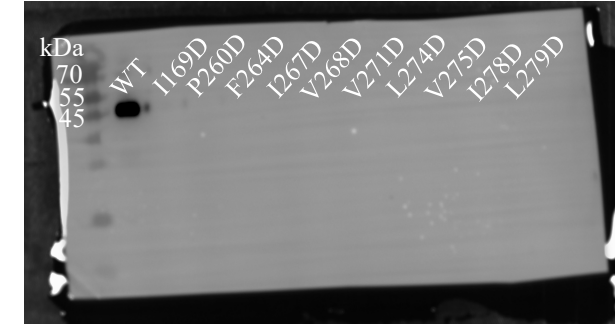

embigin (variants)

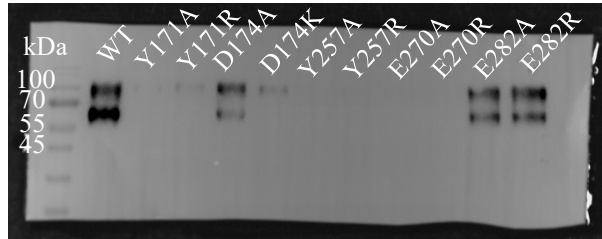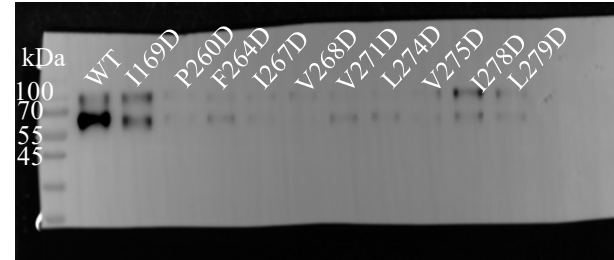

Supplement: Supplementary file 3 — Source data Fig. 1 [file 44319_2025_661_MOESM3_ESM.zip › Figure 1/Figure 1G.pdf]

**Fig. 1 H**

MCT2 (variants)

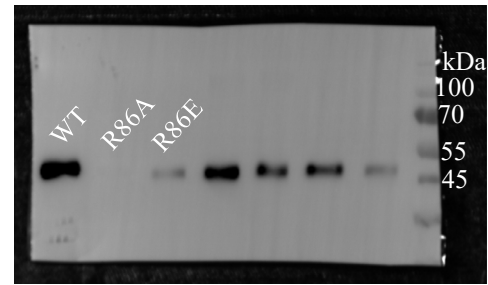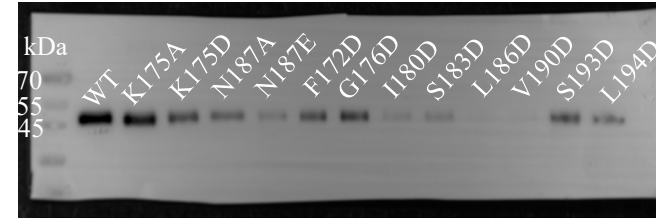

embigin (WT)

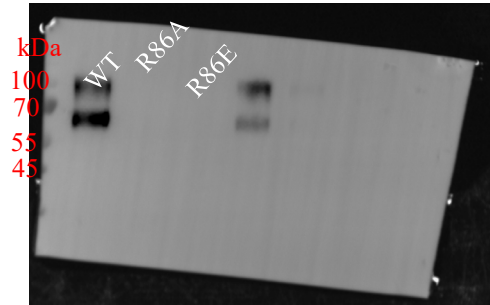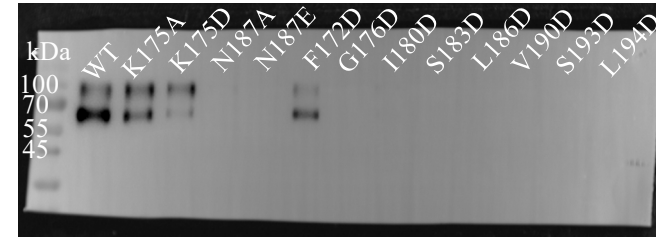

MCT2 (variants)

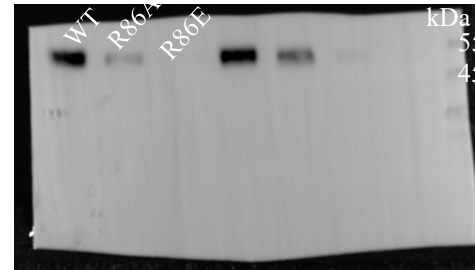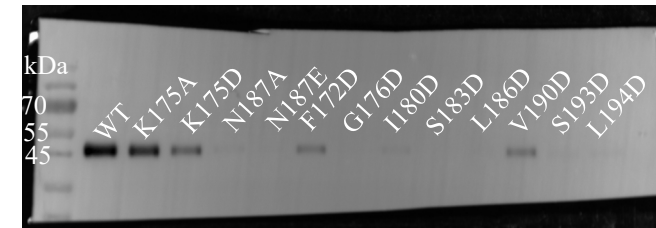

embigin (WT)

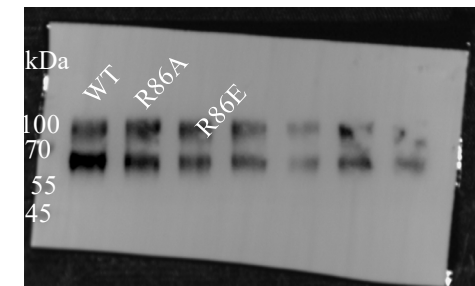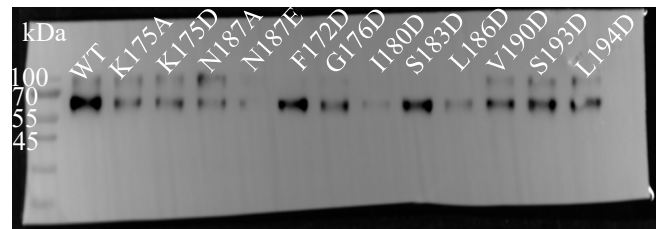

Supplement: Supplementary file 3 — Source data Fig. 1 [file 44319_2025_661_MOESM3_ESM.zip › Figure 1/Figure 1H.pdf]

**Fig. 5 B**

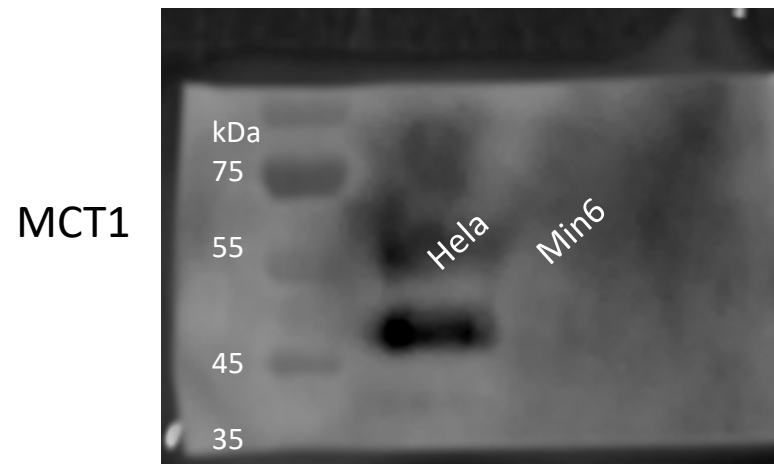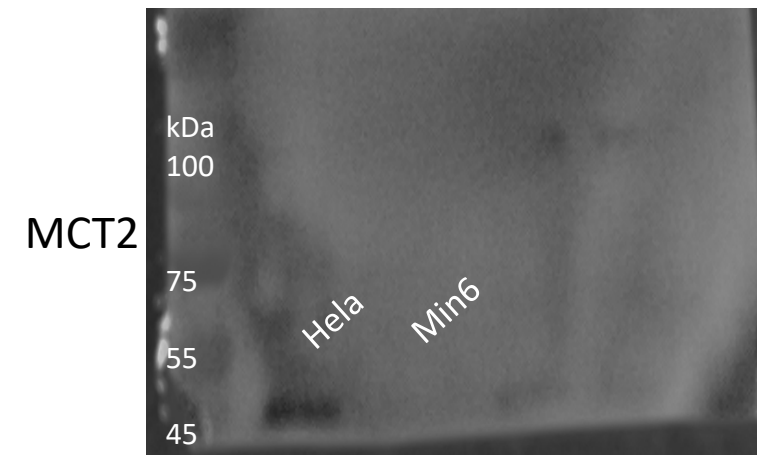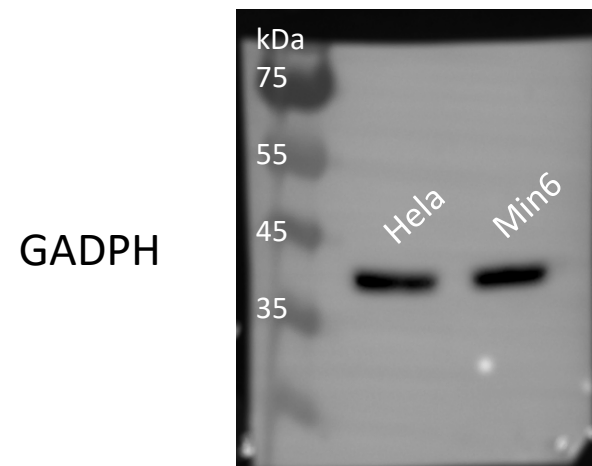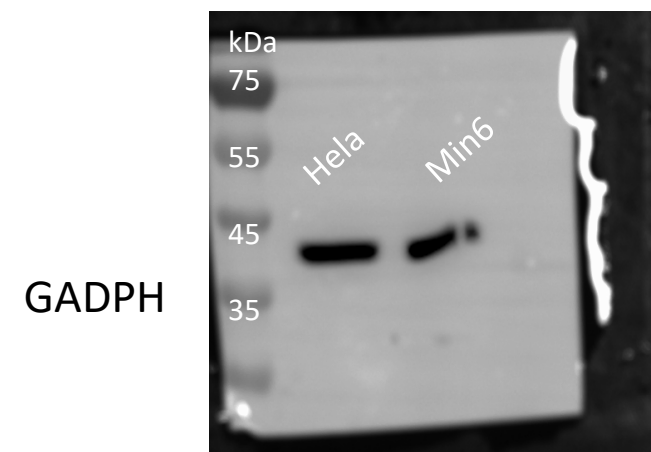

Supplement: Supplementary file 7 — Source data Fig. 5 [file 44319_2025_661_MOESM7_ESM.zip › Figure 5/Figure 5B.pdf]

**Fig. 5 I**

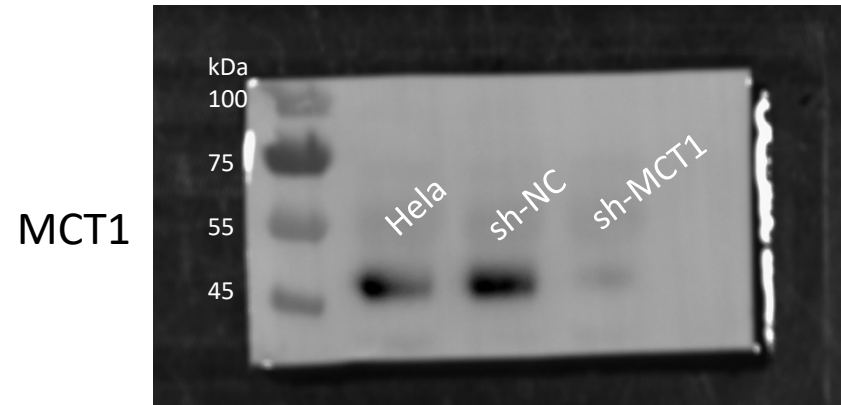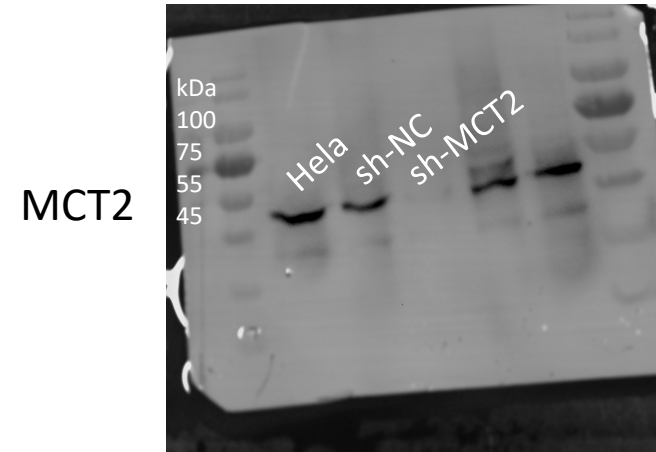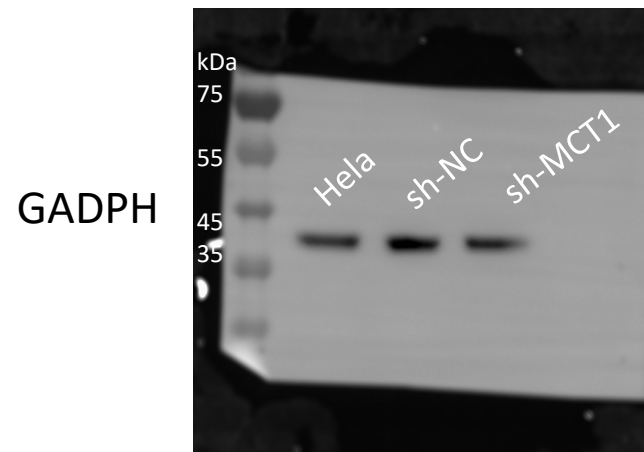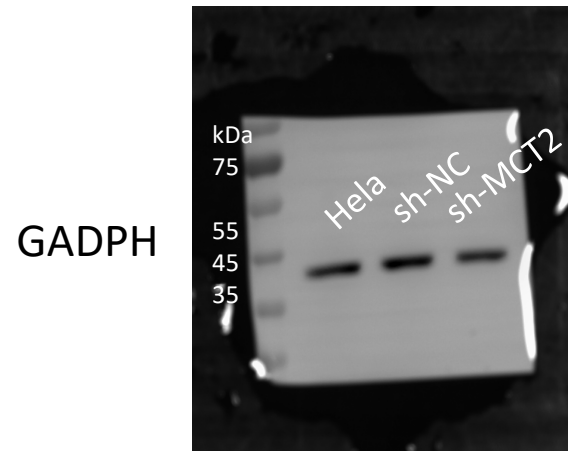

Supplement: Supplementary file 7 — Source data Fig. 5 [file 44319_2025_661_MOESM7_ESM.zip › Figure 5/Figure 5I.pdf]

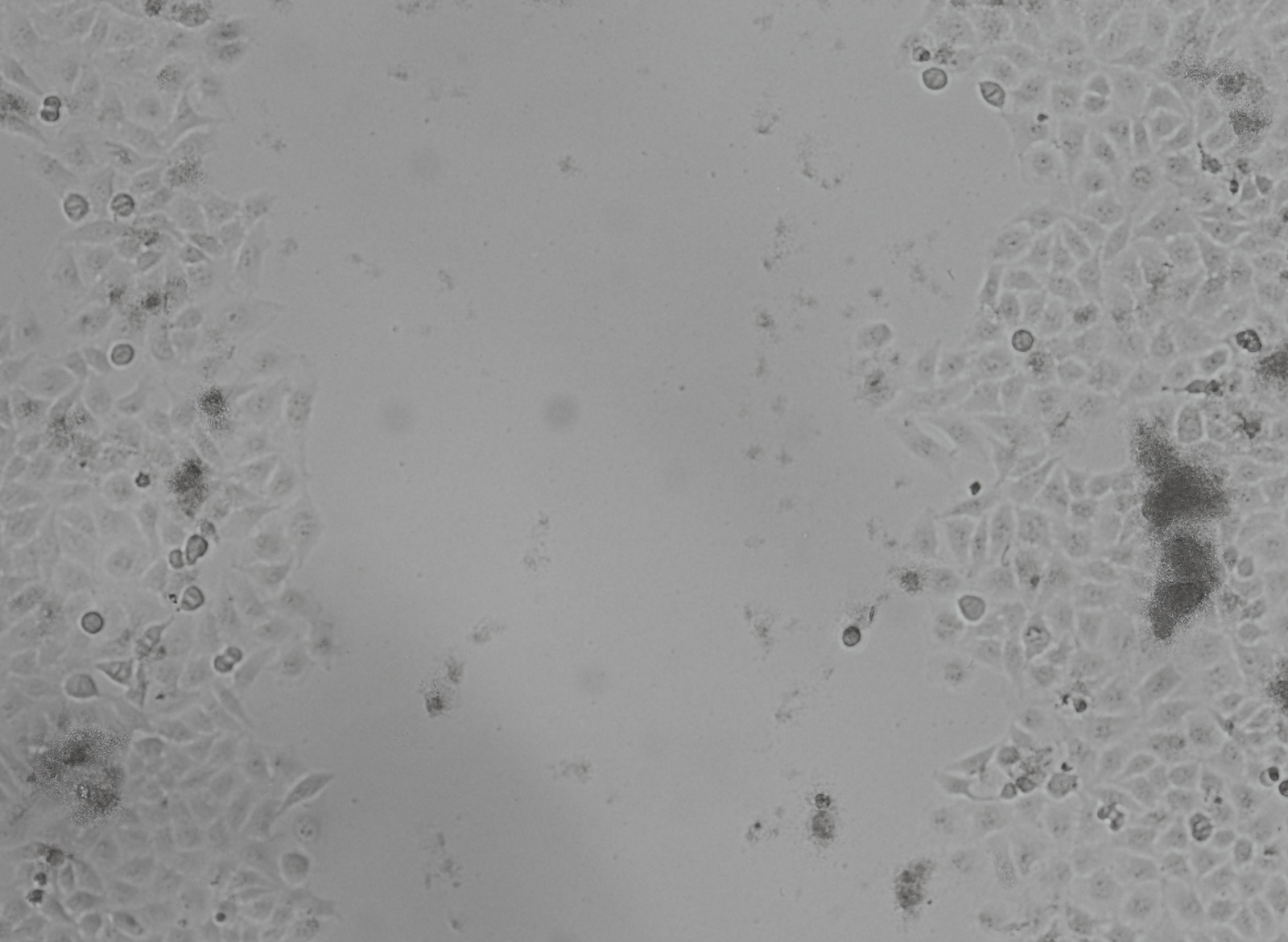

Supplement: Supplementary file 8 — Source data Fig. 6 [file 44319_2025_661_MOESM8_ESM.zip › Figure 6/Figure 6A/0h-12.5.png]

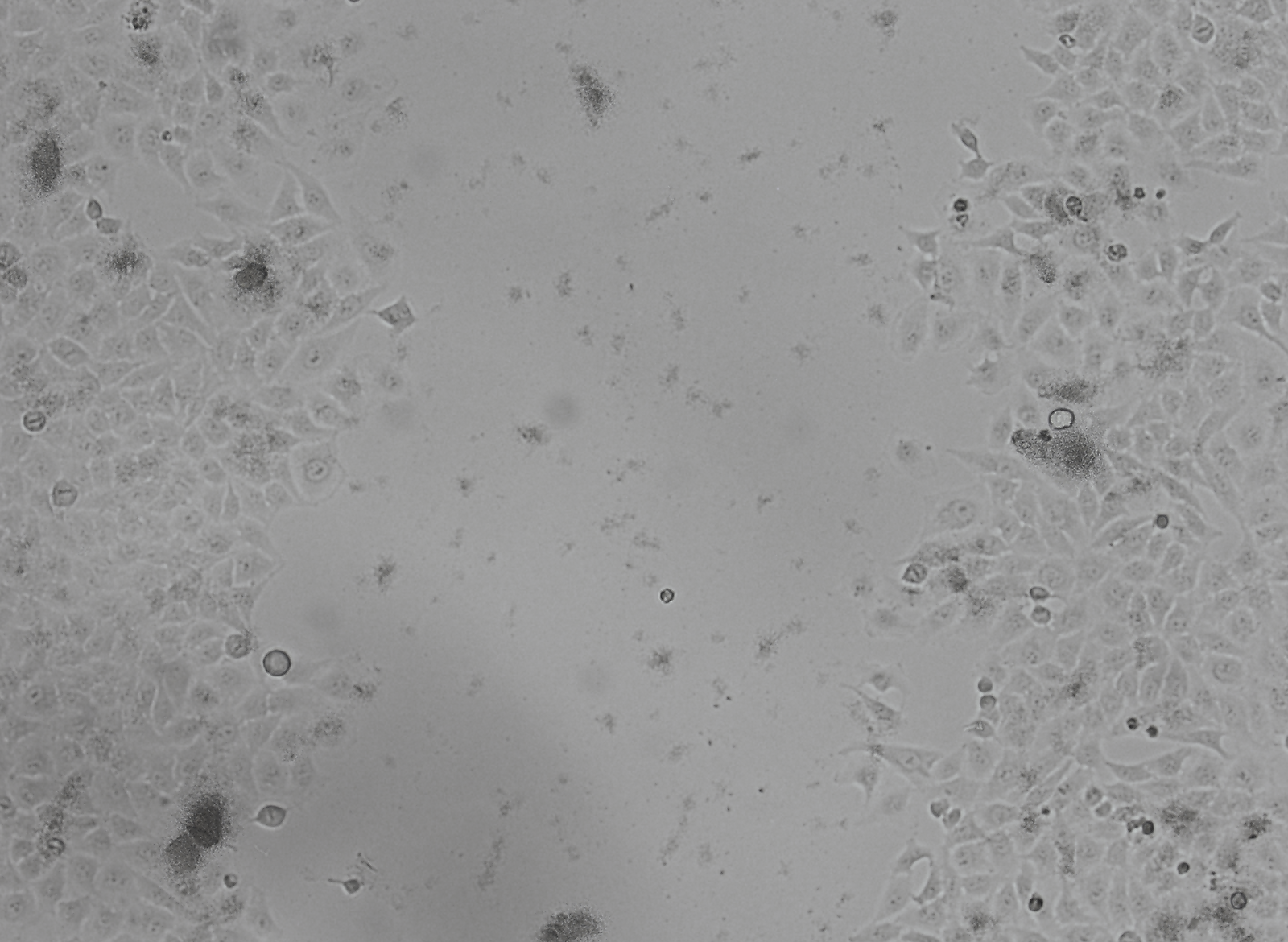

Supplement: Supplementary file 8 — Source data Fig. 6 [file 44319_2025_661_MOESM8_ESM.zip › Figure 6/Figure 6A/0h-25.png]

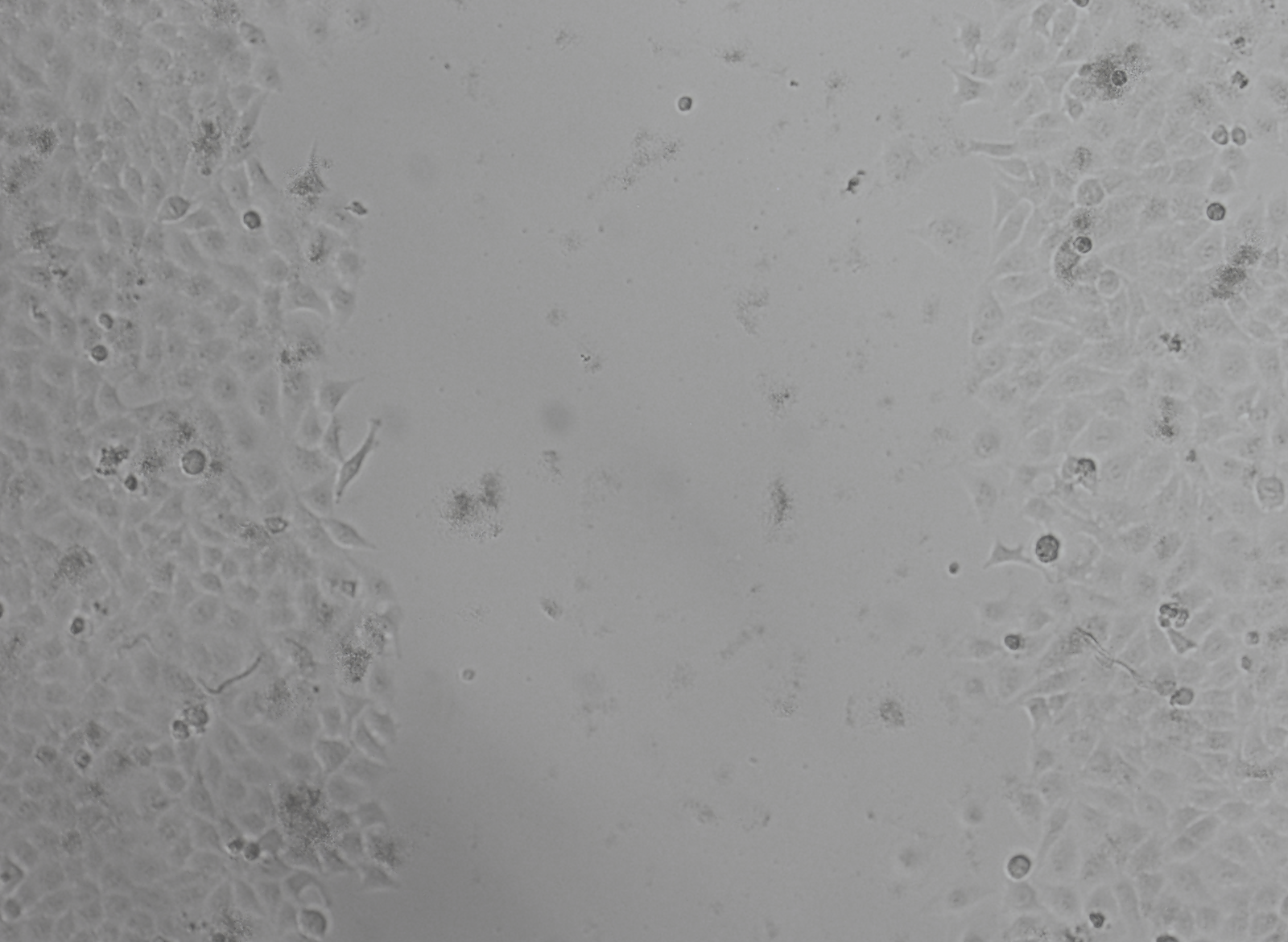

Supplement: Supplementary file 8 — Source data Fig. 6 [file 44319_2025_661_MOESM8_ESM.zip › Figure 6/Figure 6A/0h-6.25.png]

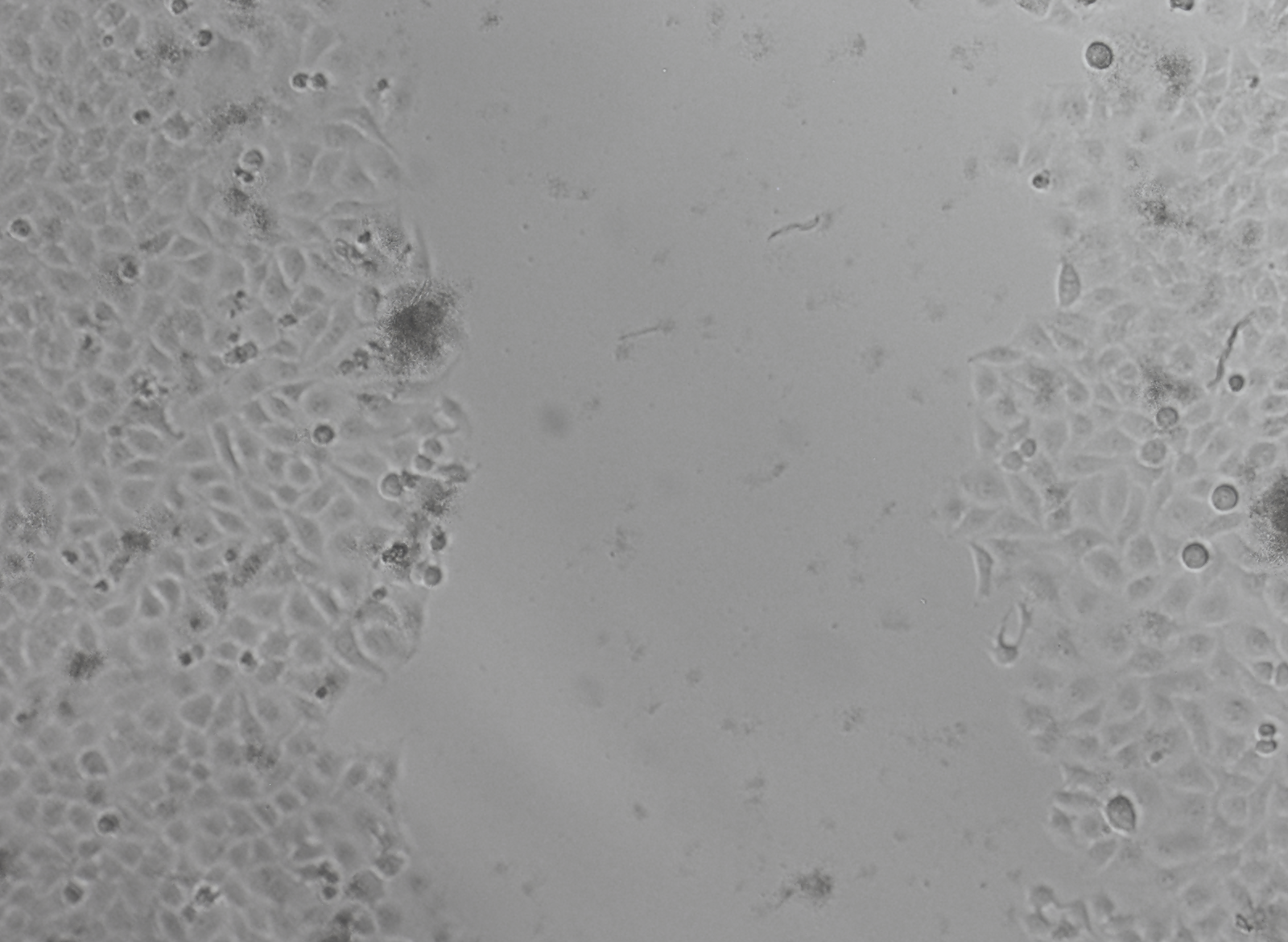

Supplement: Supplementary file 8 — Source data Fig. 6 [file 44319_2025_661_MOESM8_ESM.zip › Figure 6/Figure 6A/0h-Control.png]

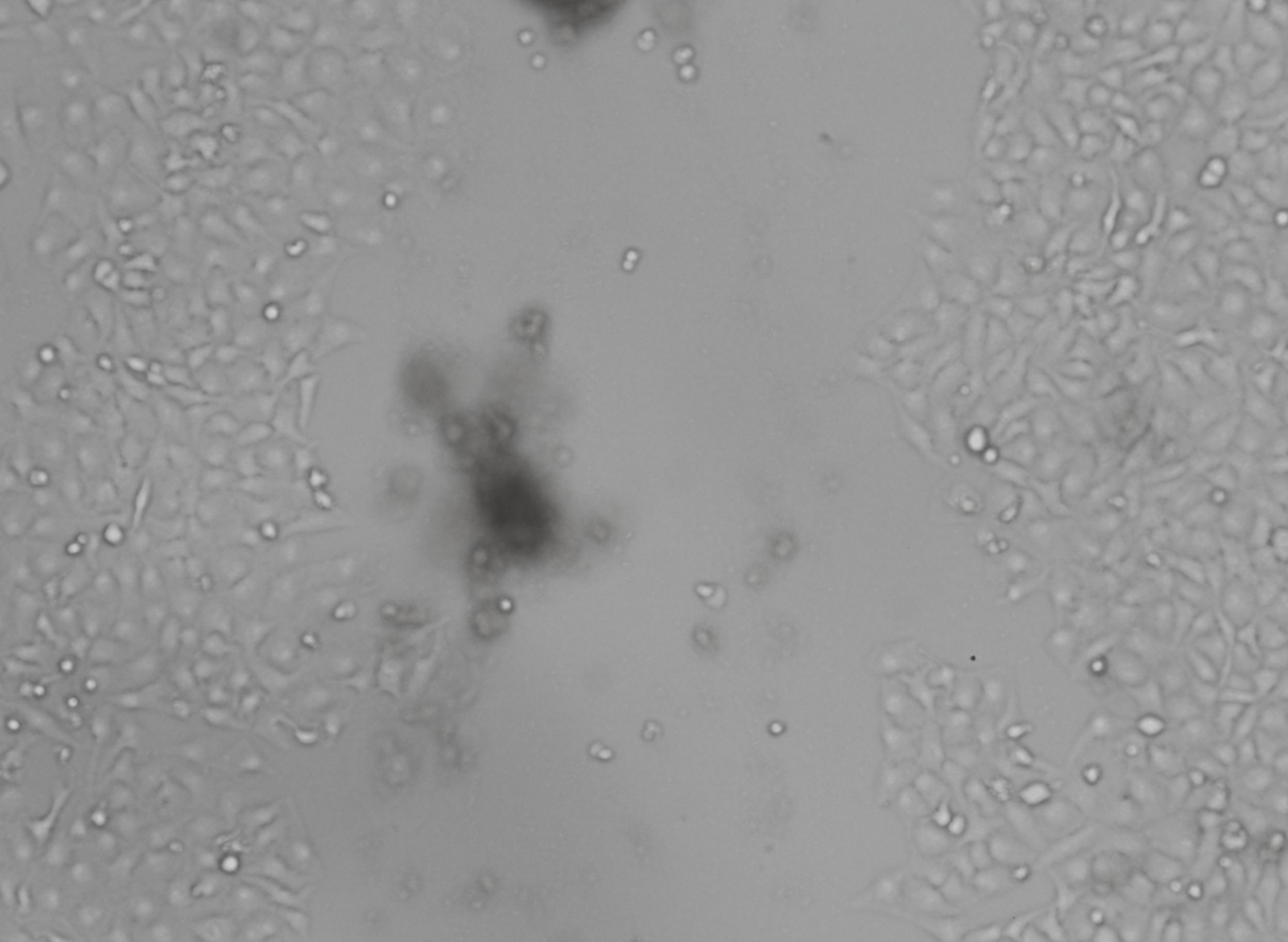

Supplement: Supplementary file 8 — Source data Fig. 6 [file 44319_2025_661_MOESM8_ESM.zip › Figure 6/Figure 6A/24h-12.5.png]

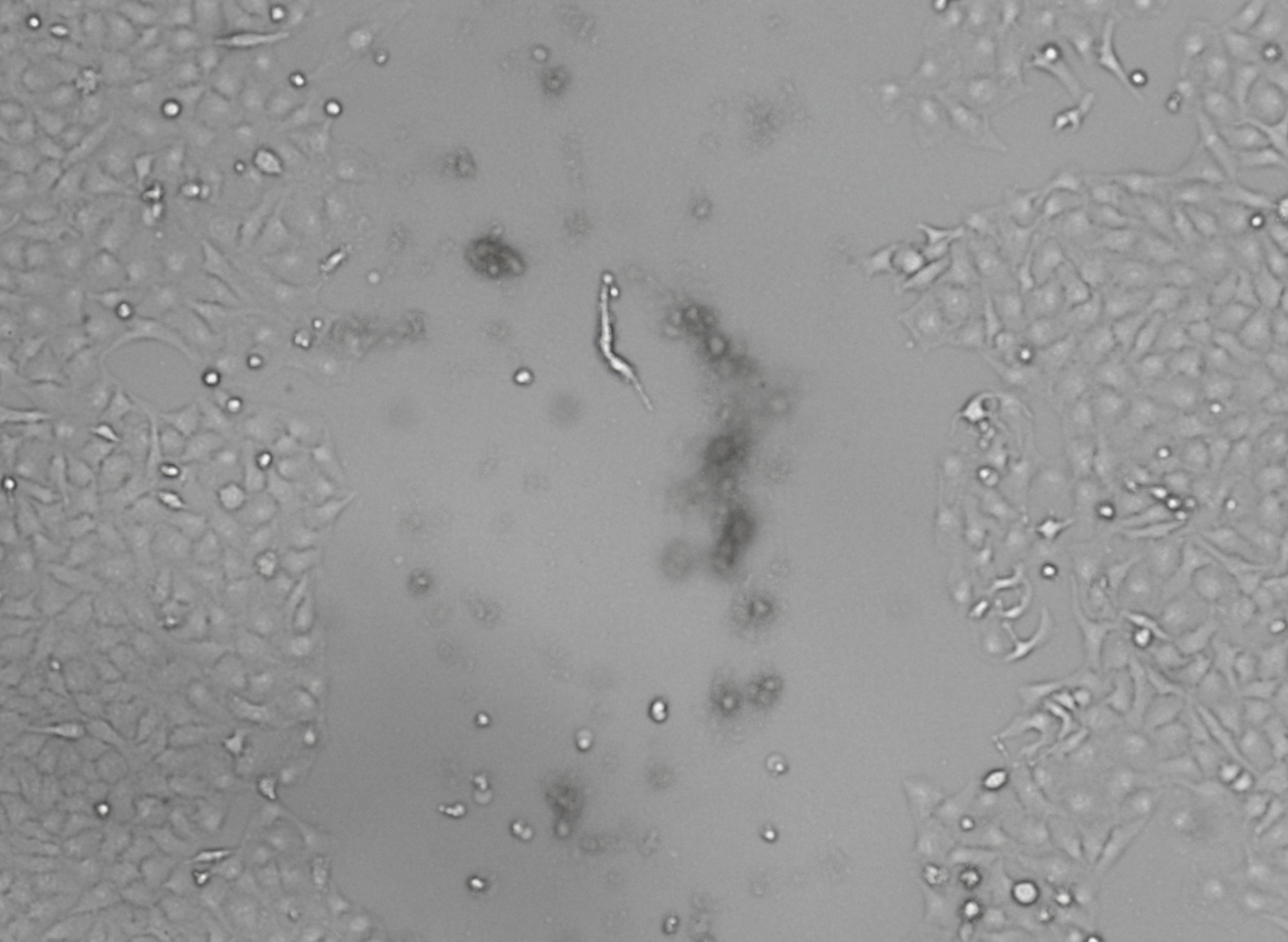

Supplement: Supplementary file 8 — Source data Fig. 6 [file 44319_2025_661_MOESM8_ESM.zip › Figure 6/Figure 6A/24h-25.png]

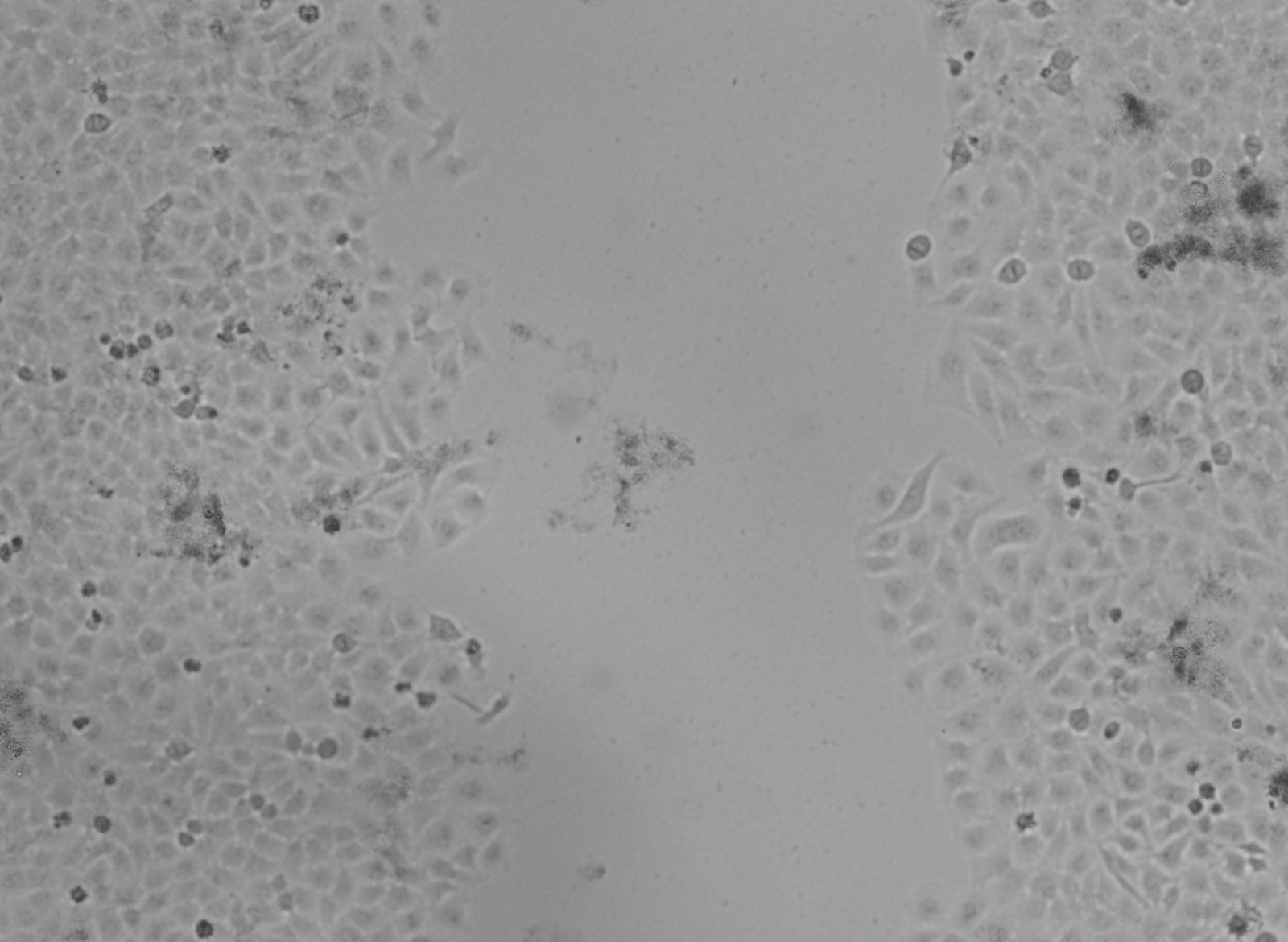

Supplement: Supplementary file 8 — Source data Fig. 6 [file 44319_2025_661_MOESM8_ESM.zip › Figure 6/Figure 6A/24h-6.25.png]

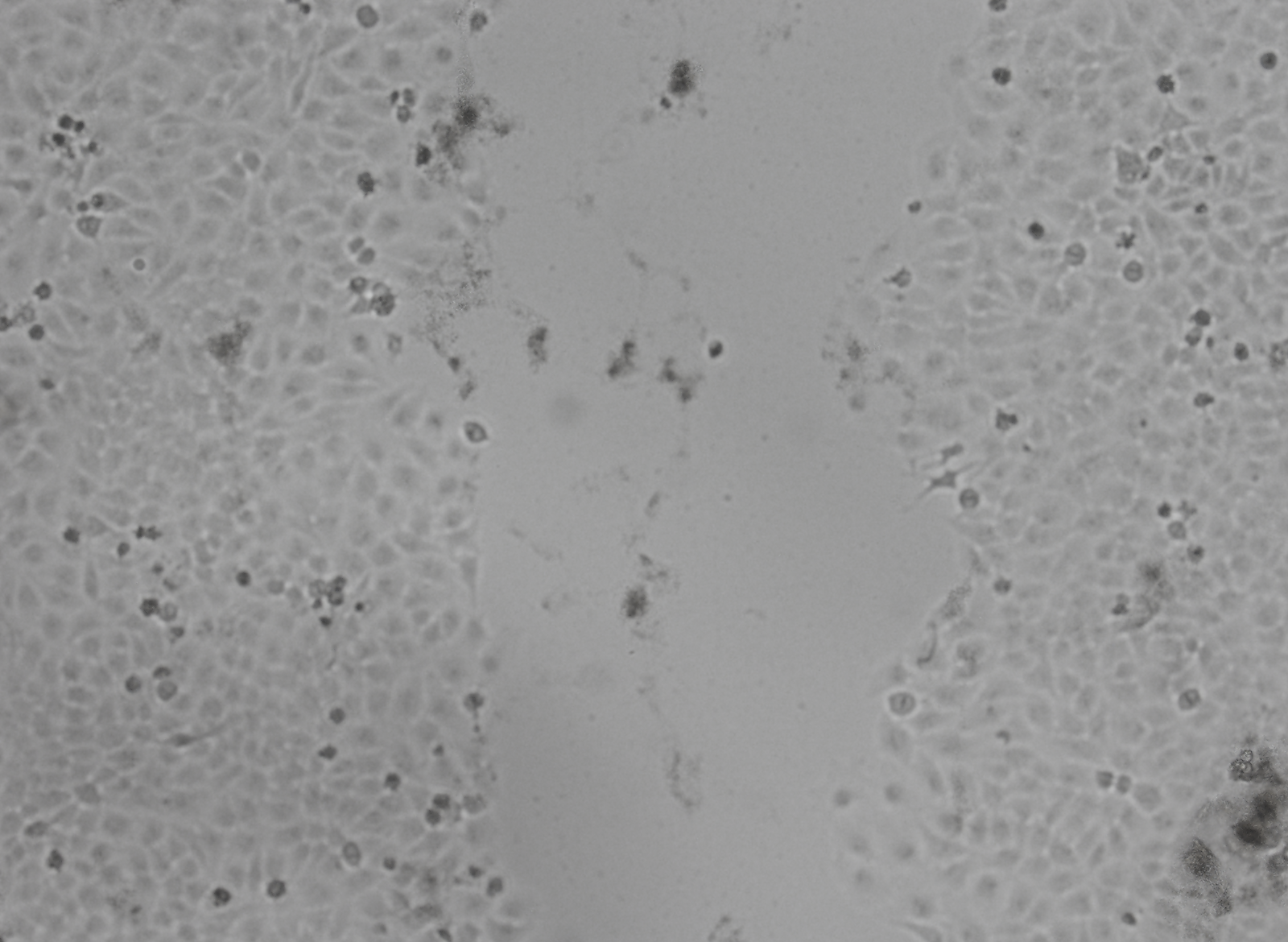

Supplement: Supplementary file 8 — Source data Fig. 6 [file 44319_2025_661_MOESM8_ESM.zip › Figure 6/Figure 6A/24h-Control.png]

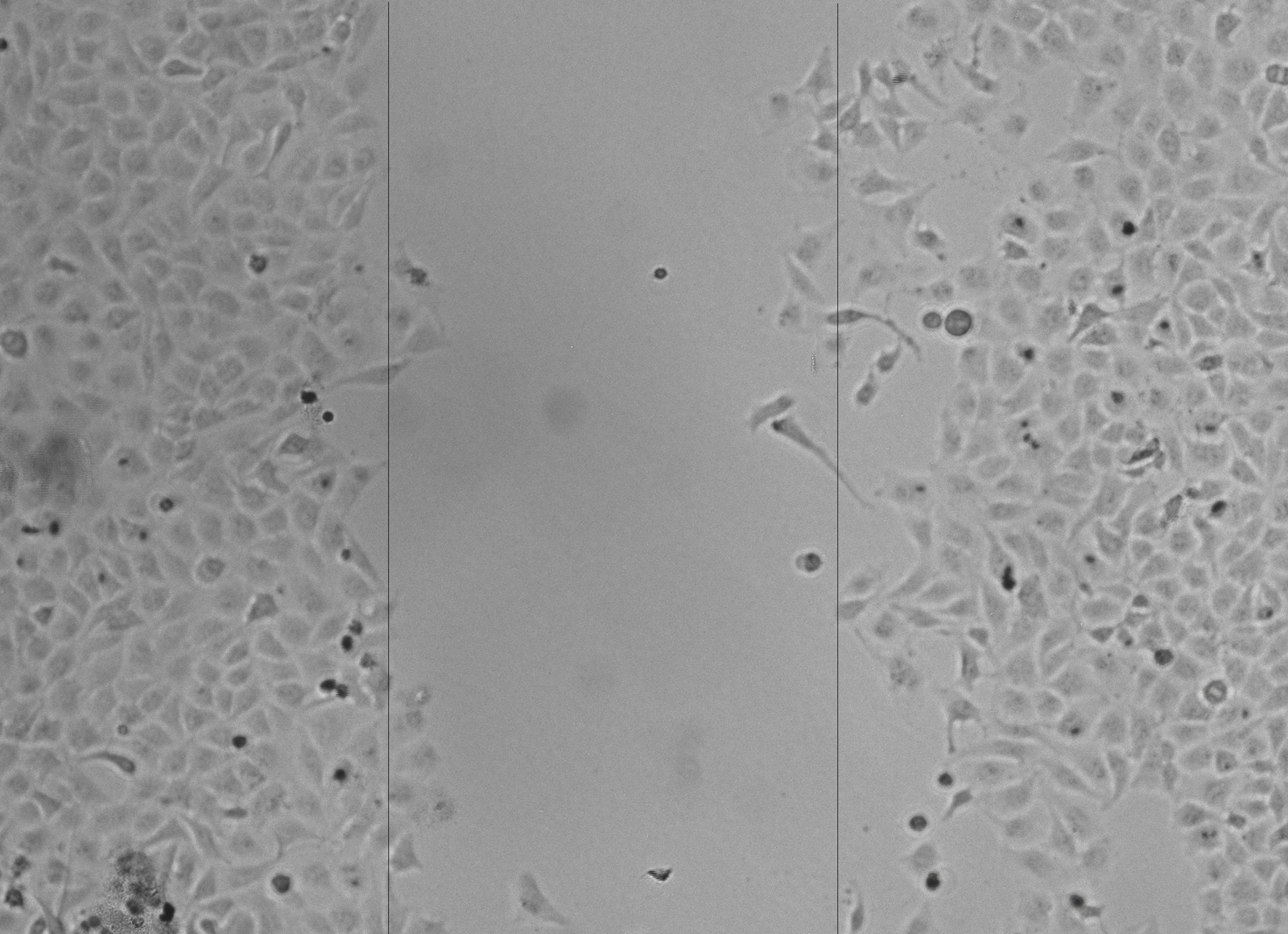

Supplement: Supplementary file 8 — Source data Fig. 6 [file 44319_2025_661_MOESM8_ESM.zip › Figure 6/Figure 6A/48-25.png]

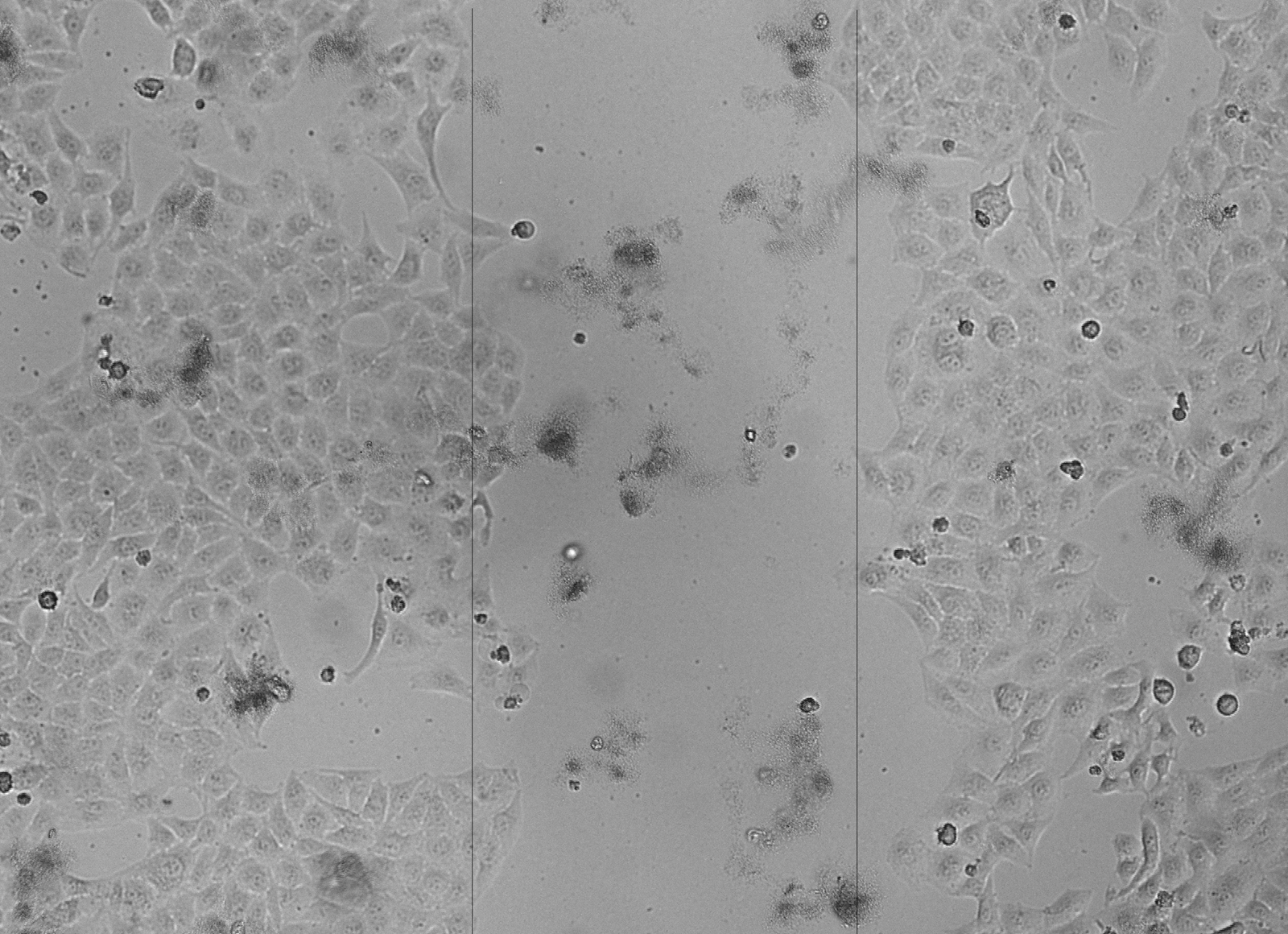

Supplement: Supplementary file 8 — Source data Fig. 6 [file 44319_2025_661_MOESM8_ESM.zip › Figure 6/Figure 6A/48-6.25.png]

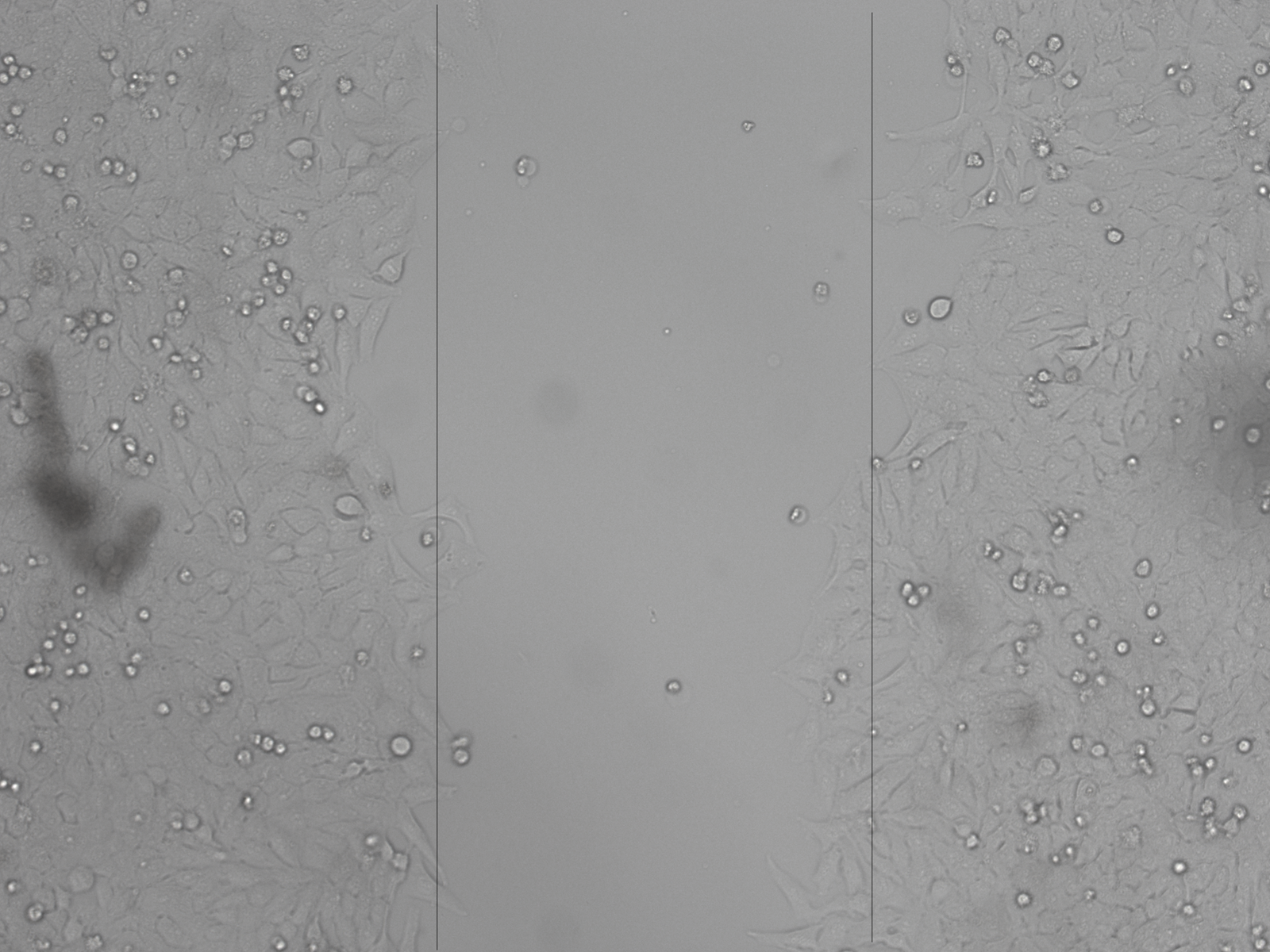

Supplement: Supplementary file 8 — Source data Fig. 6 [file 44319_2025_661_MOESM8_ESM.zip › Figure 6/Figure 6A/48h-12.5.png]

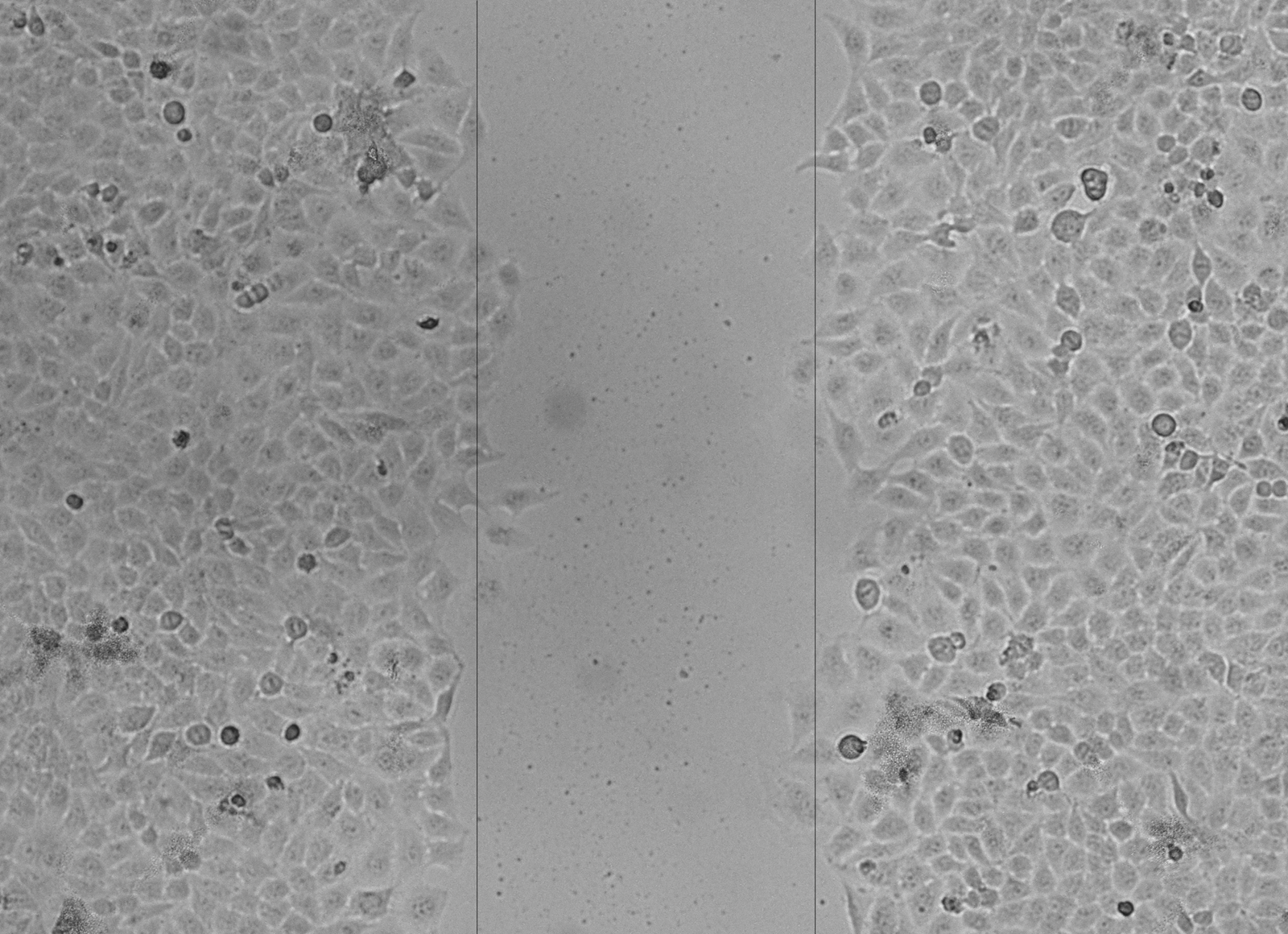

Supplement: Supplementary file 8 — Source data Fig. 6 [file 44319_2025_661_MOESM8_ESM.zip › Figure 6/Figure 6A/48h-Control.png]

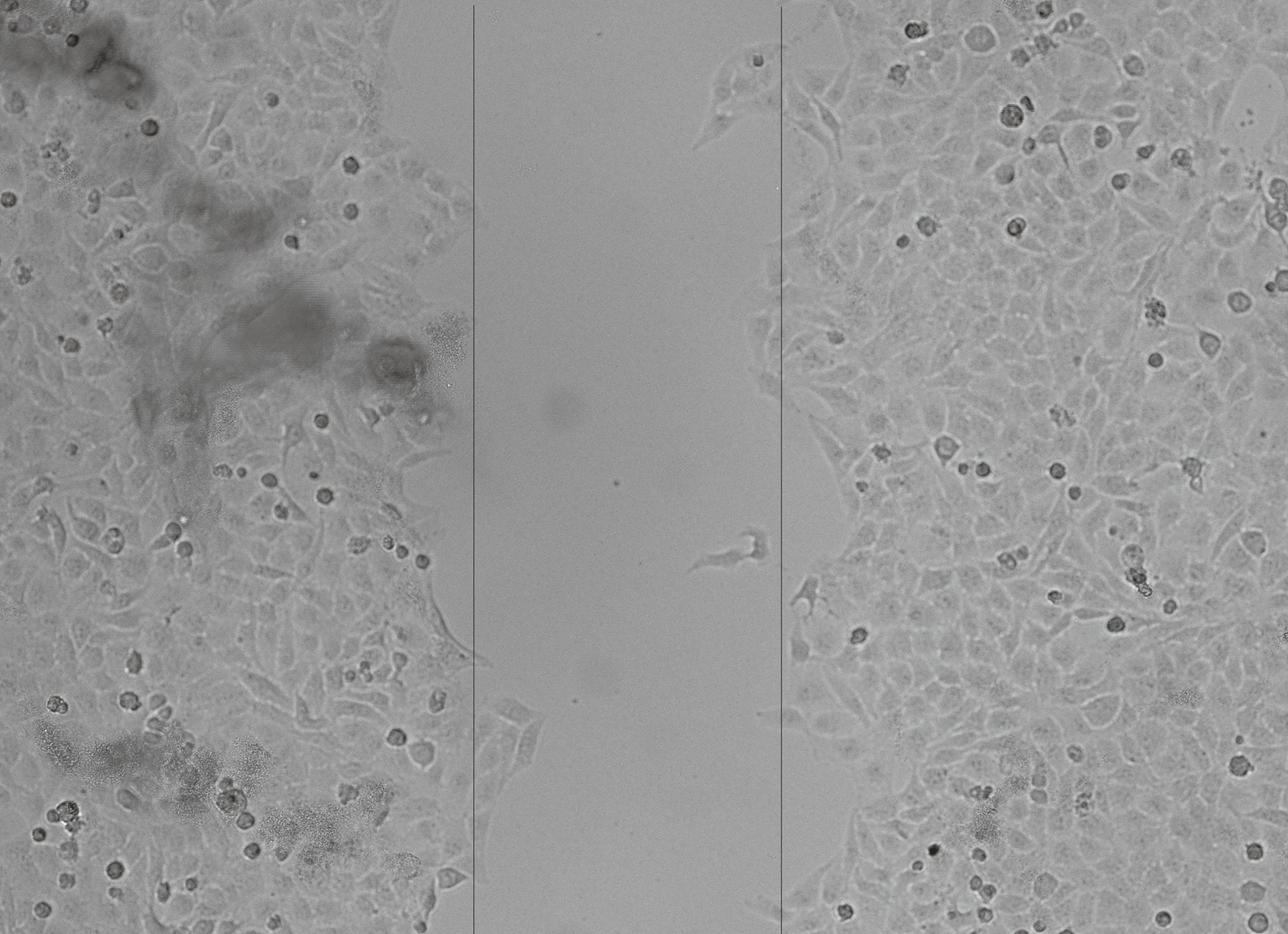

Supplement: Supplementary file 8 — Source data Fig. 6 [file 44319_2025_661_MOESM8_ESM.zip › Figure 6/Figure 6A/72-12.5.png]

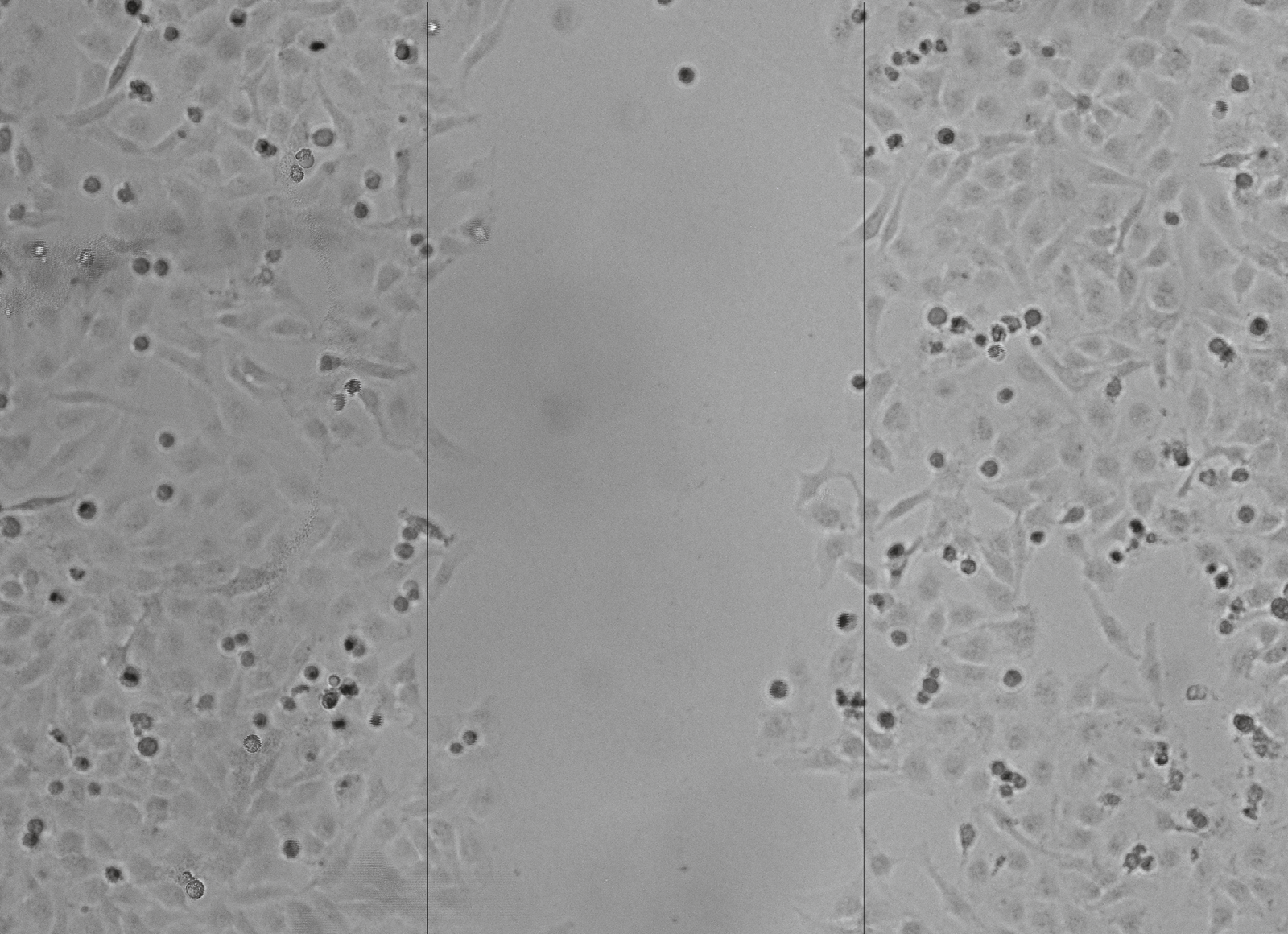

Supplement: Supplementary file 8 — Source data Fig. 6 [file 44319_2025_661_MOESM8_ESM.zip › Figure 6/Figure 6A/72-25.png]

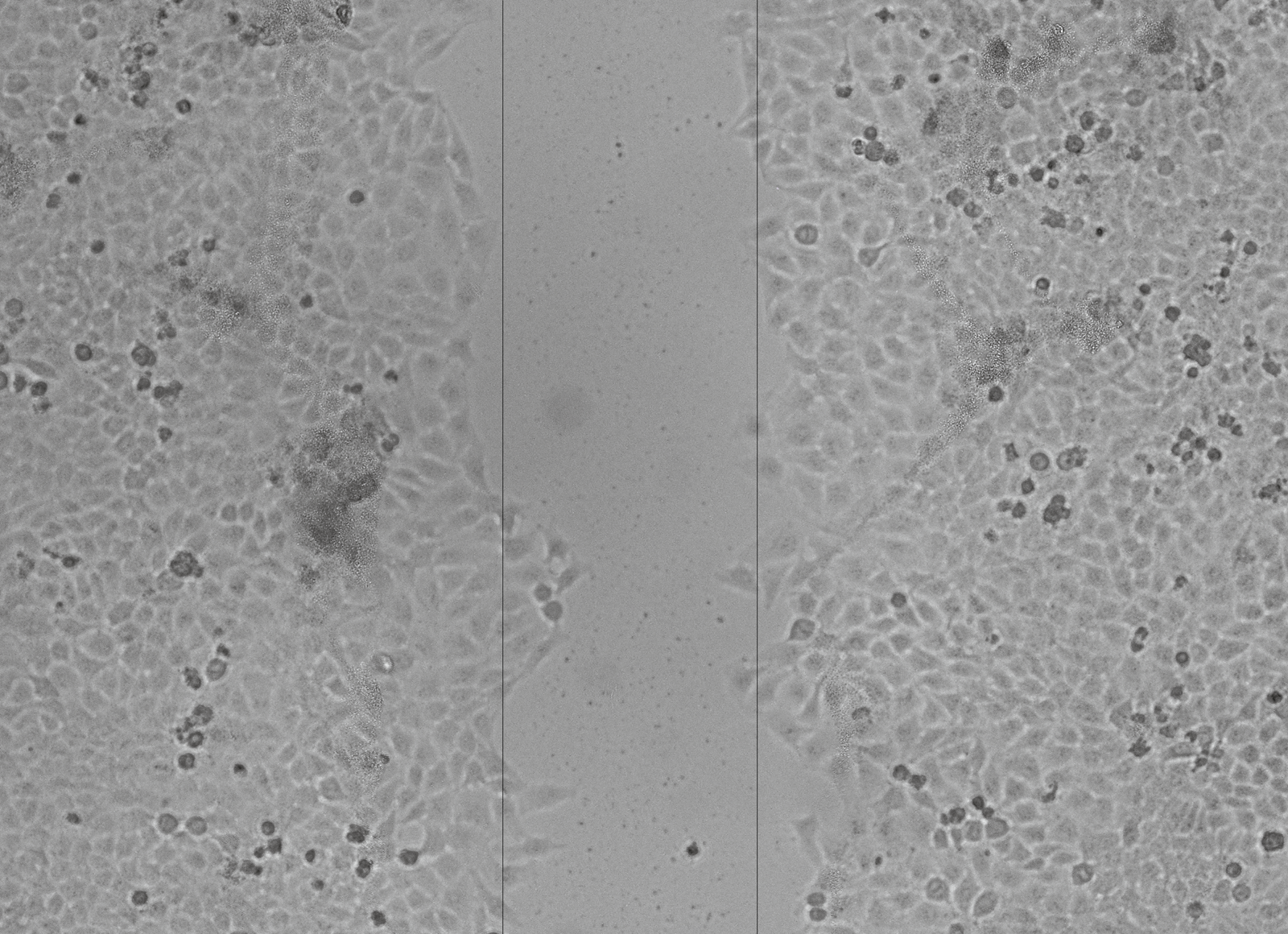

Supplement: Supplementary file 8 — Source data Fig. 6 [file 44319_2025_661_MOESM8_ESM.zip › Figure 6/Figure 6A/72-6.25.png]

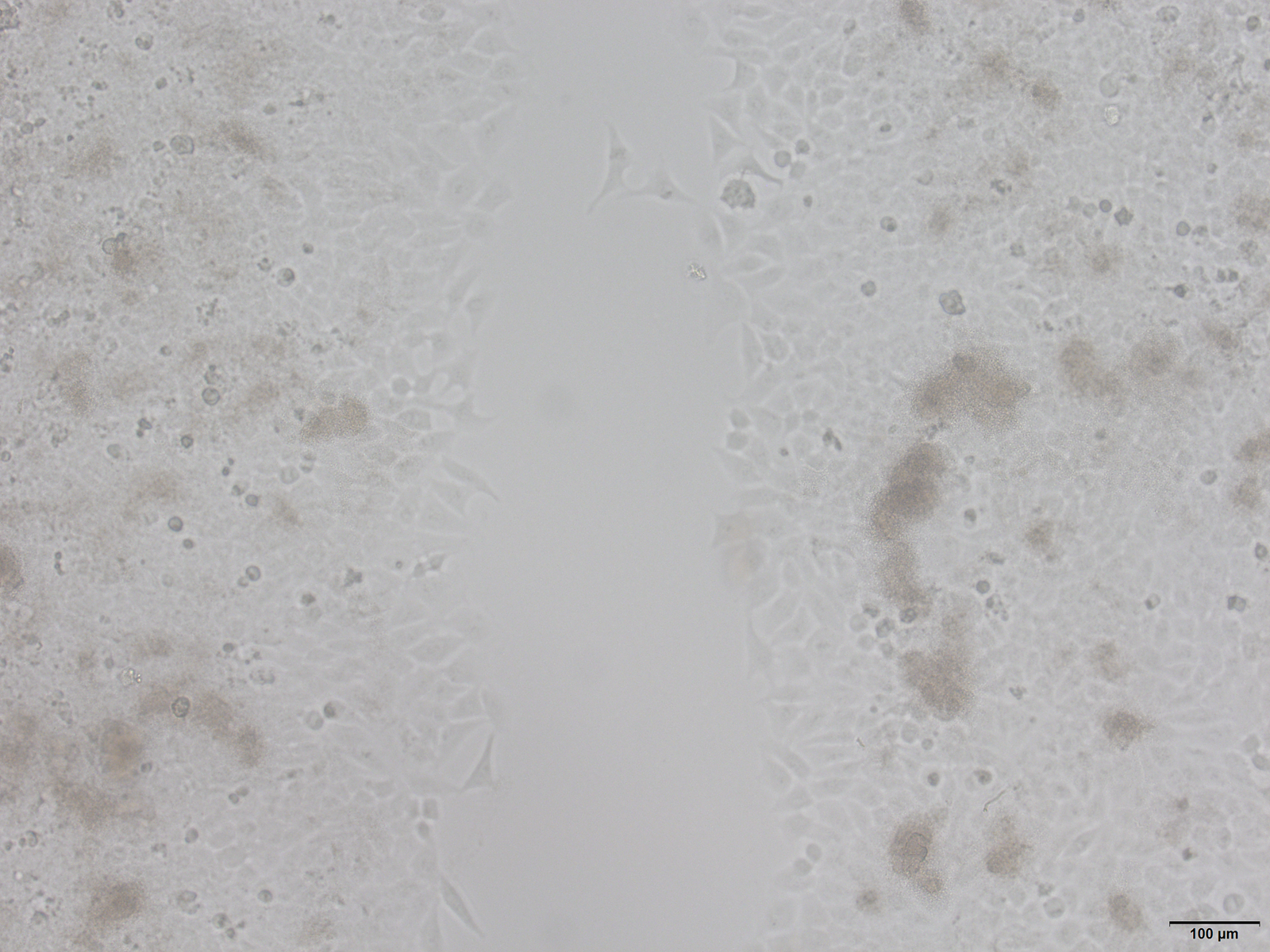

Supplement: Supplementary file 8 — Source data Fig. 6 [file 44319_2025_661_MOESM8_ESM.zip › Figure 6/Figure 6A/72h-Control.png]

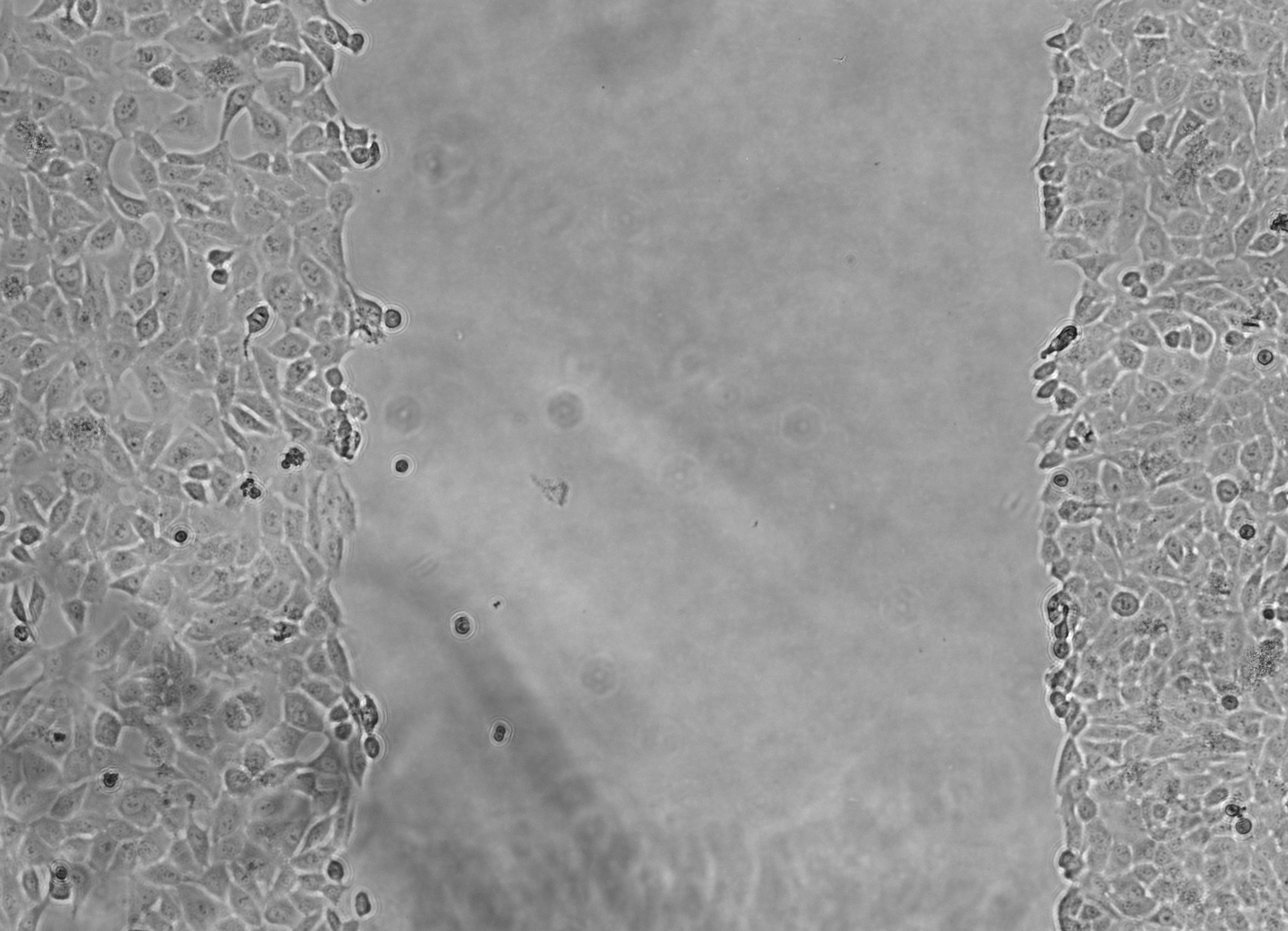

Supplement: Supplementary file 8 — Source data Fig. 6 [file 44319_2025_661_MOESM8_ESM.zip › Figure 6/Figure 6C/0h-12.5.png]

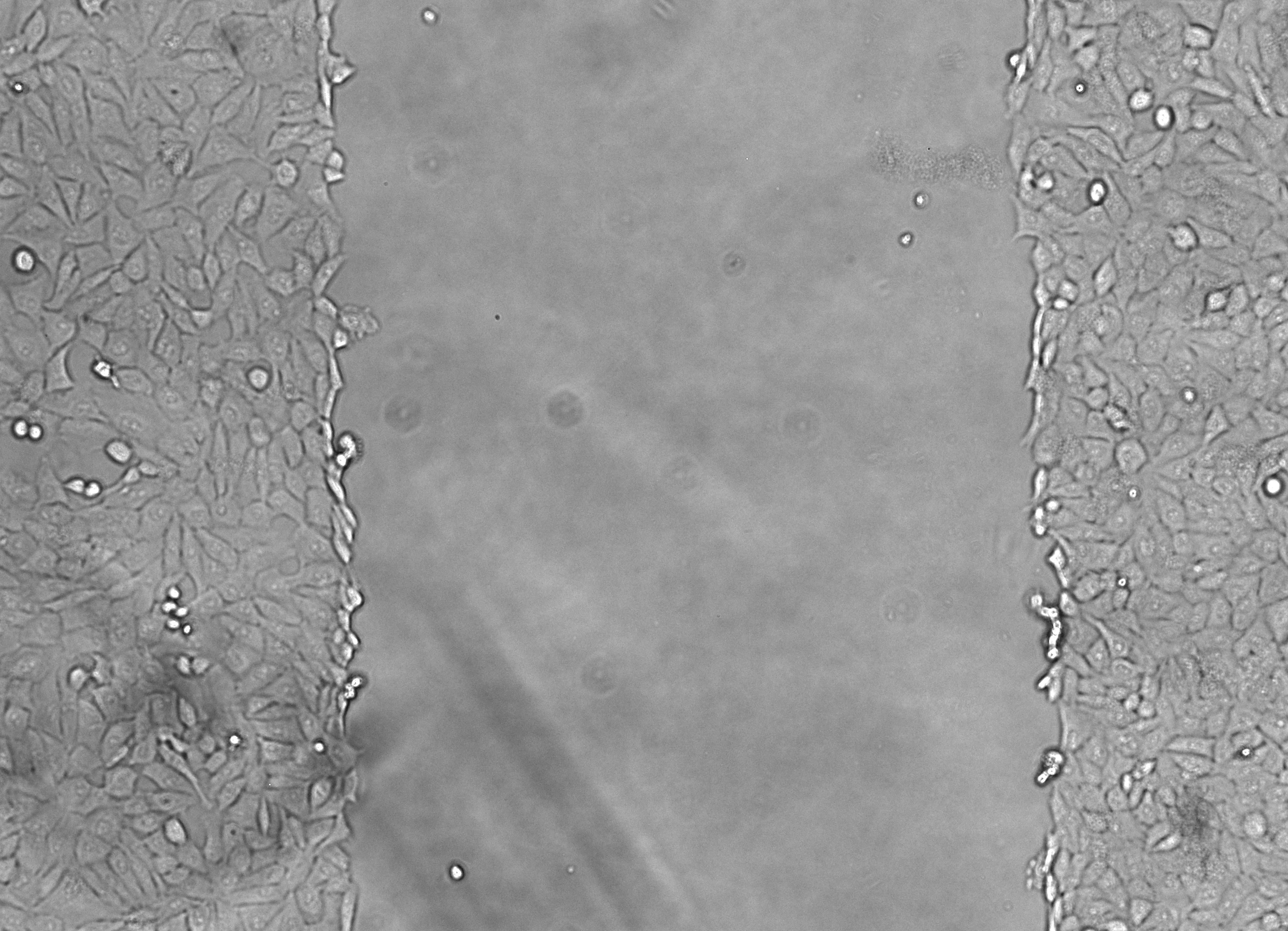

Supplement: Supplementary file 8 — Source data Fig. 6 [file 44319_2025_661_MOESM8_ESM.zip › Figure 6/Figure 6C/0h-25.png]

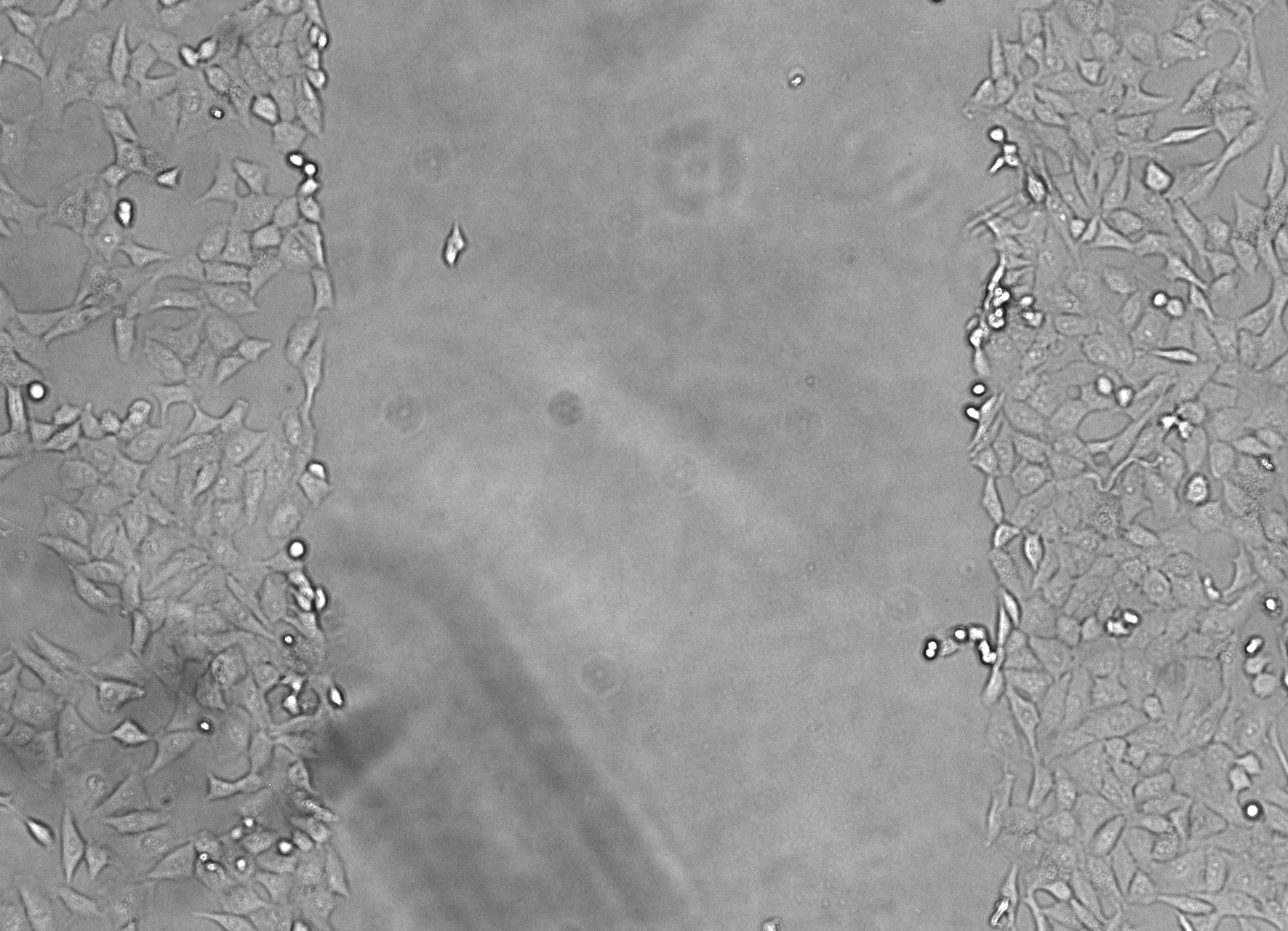

Supplement: Supplementary file 8 — Source data Fig. 6 [file 44319_2025_661_MOESM8_ESM.zip › Figure 6/Figure 6C/0h-50.png]

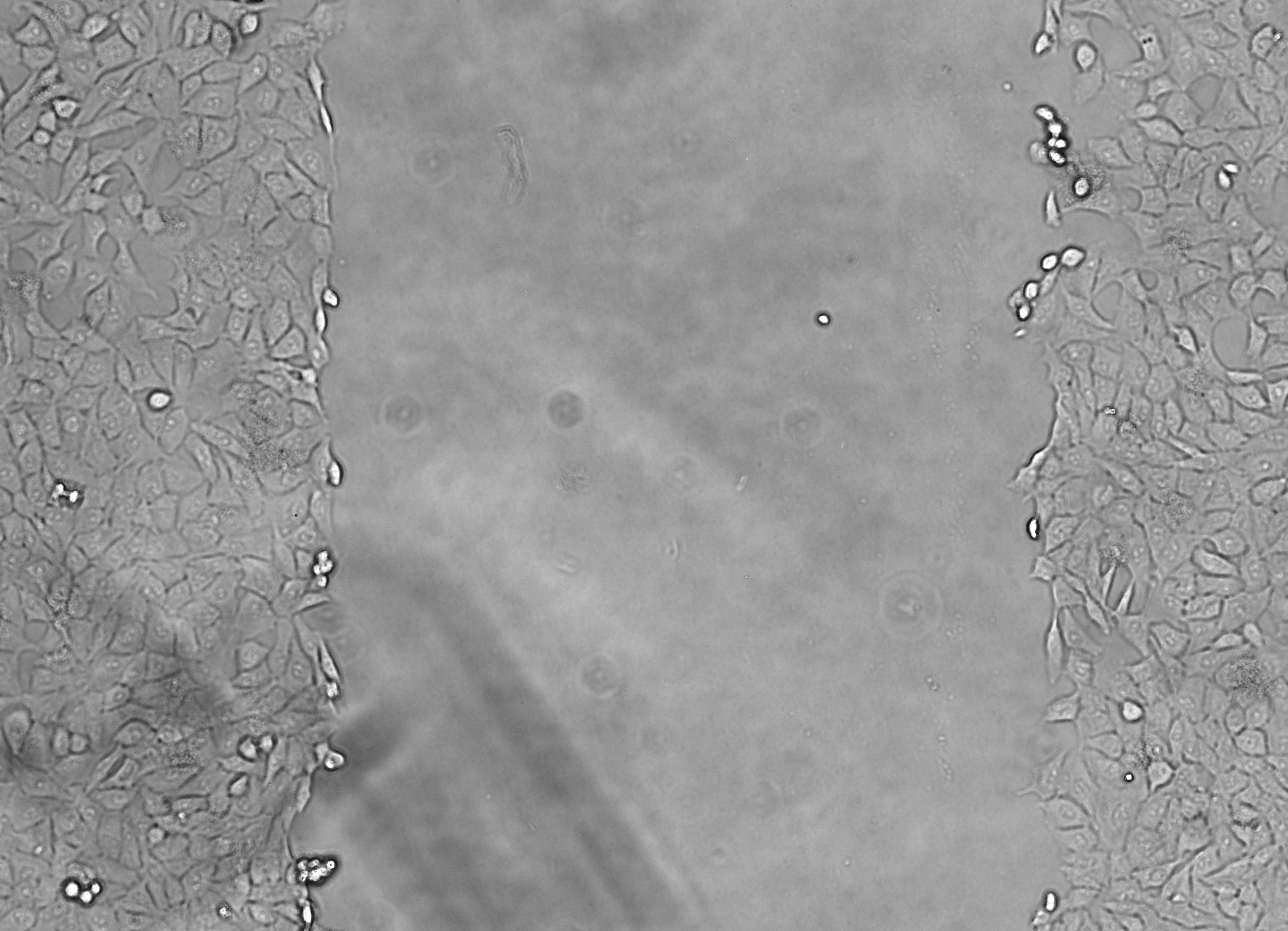

Supplement: Supplementary file 8 — Source data Fig. 6 [file 44319_2025_661_MOESM8_ESM.zip › Figure 6/Figure 6C/0h-Control.png]

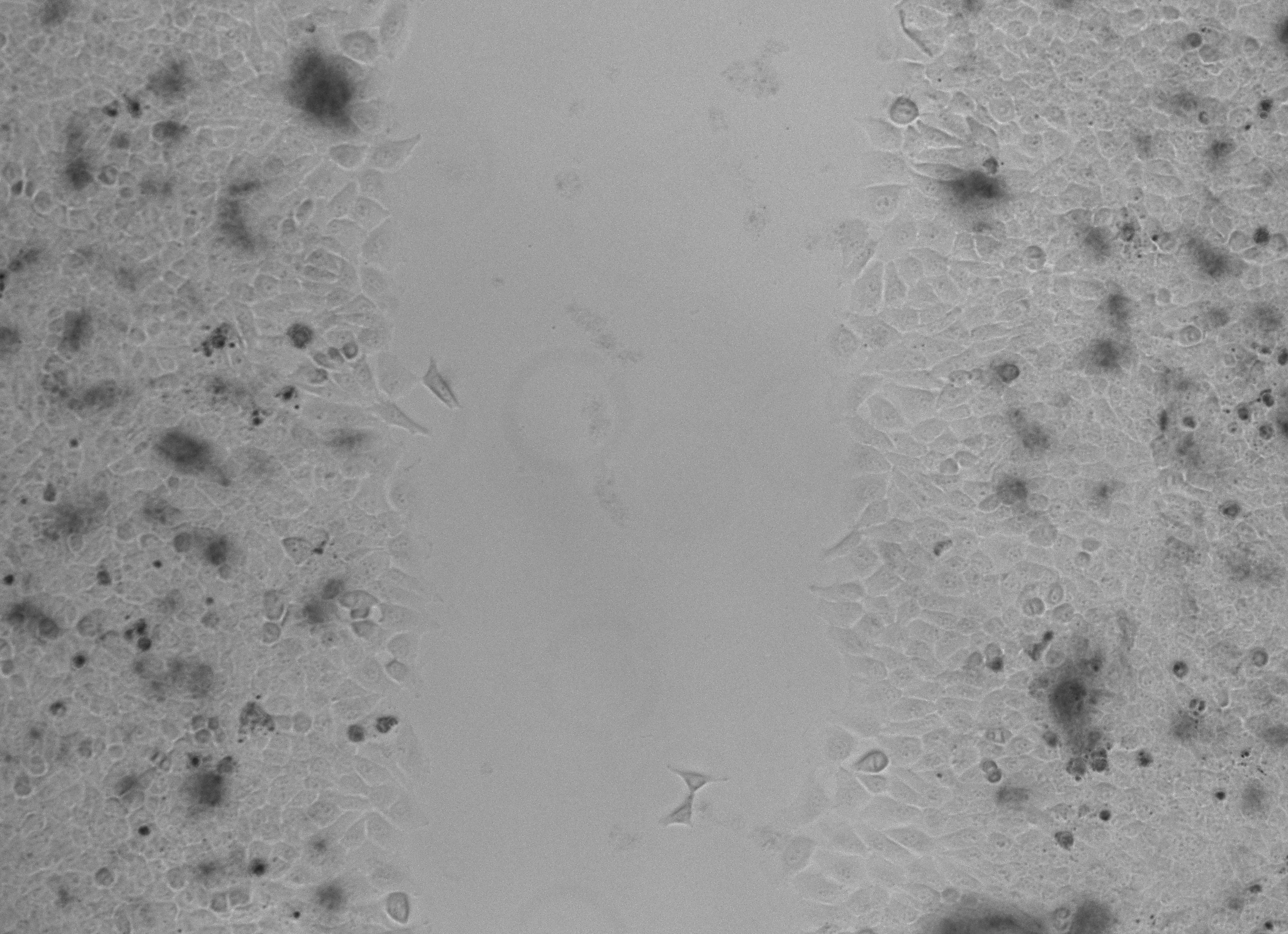

Supplement: Supplementary file 8 — Source data Fig. 6 [file 44319_2025_661_MOESM8_ESM.zip › Figure 6/Figure 6C/24h-12.5.png]

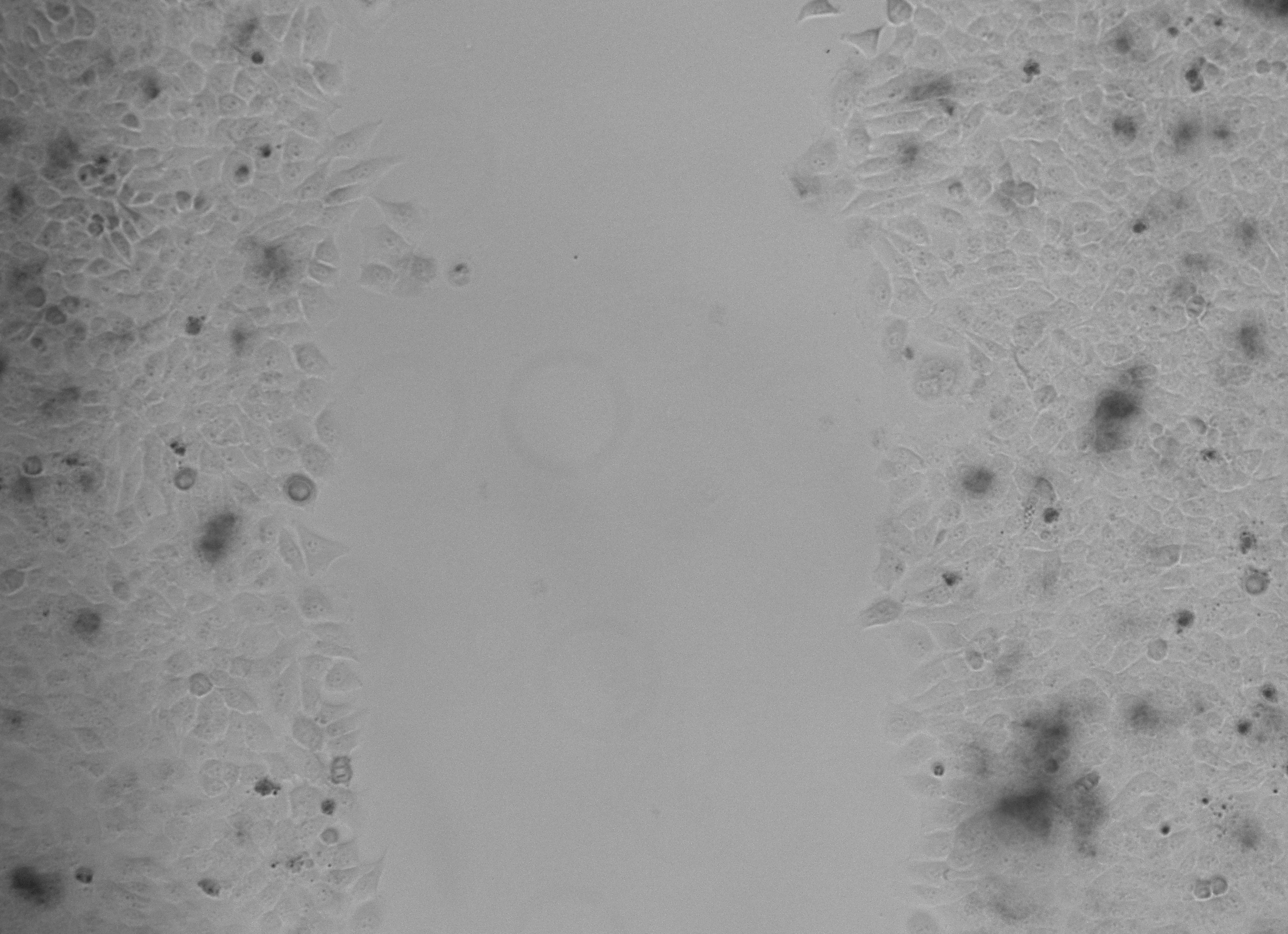

Supplement: Supplementary file 8 — Source data Fig. 6 [file 44319_2025_661_MOESM8_ESM.zip › Figure 6/Figure 6C/24h-25.png]

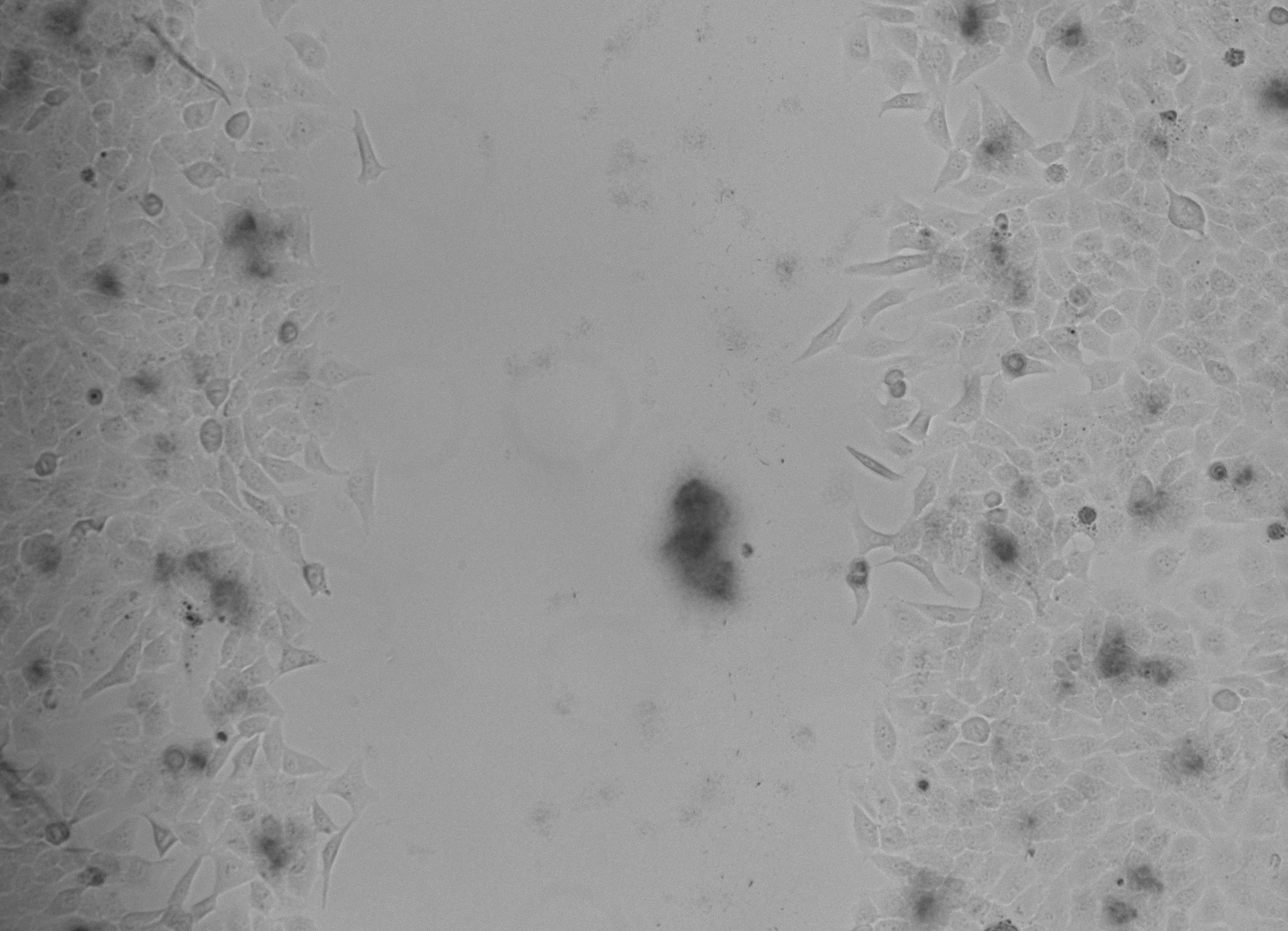

Supplement: Supplementary file 8 — Source data Fig. 6 [file 44319_2025_661_MOESM8_ESM.zip › Figure 6/Figure 6C/24h-50.png]

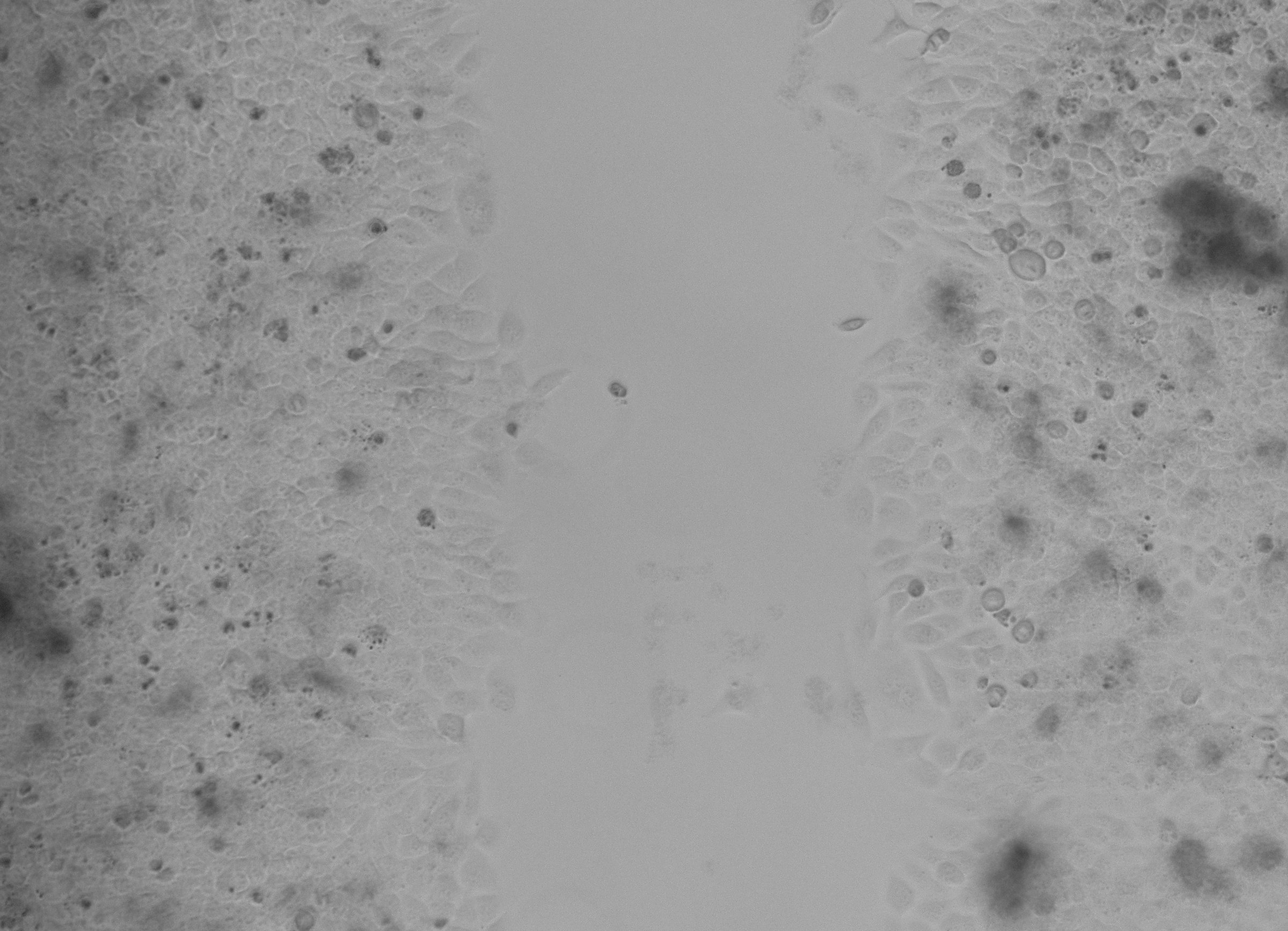

Supplement: Supplementary file 8 — Source data Fig. 6 [file 44319_2025_661_MOESM8_ESM.zip › Figure 6/Figure 6C/24h-Control.png]

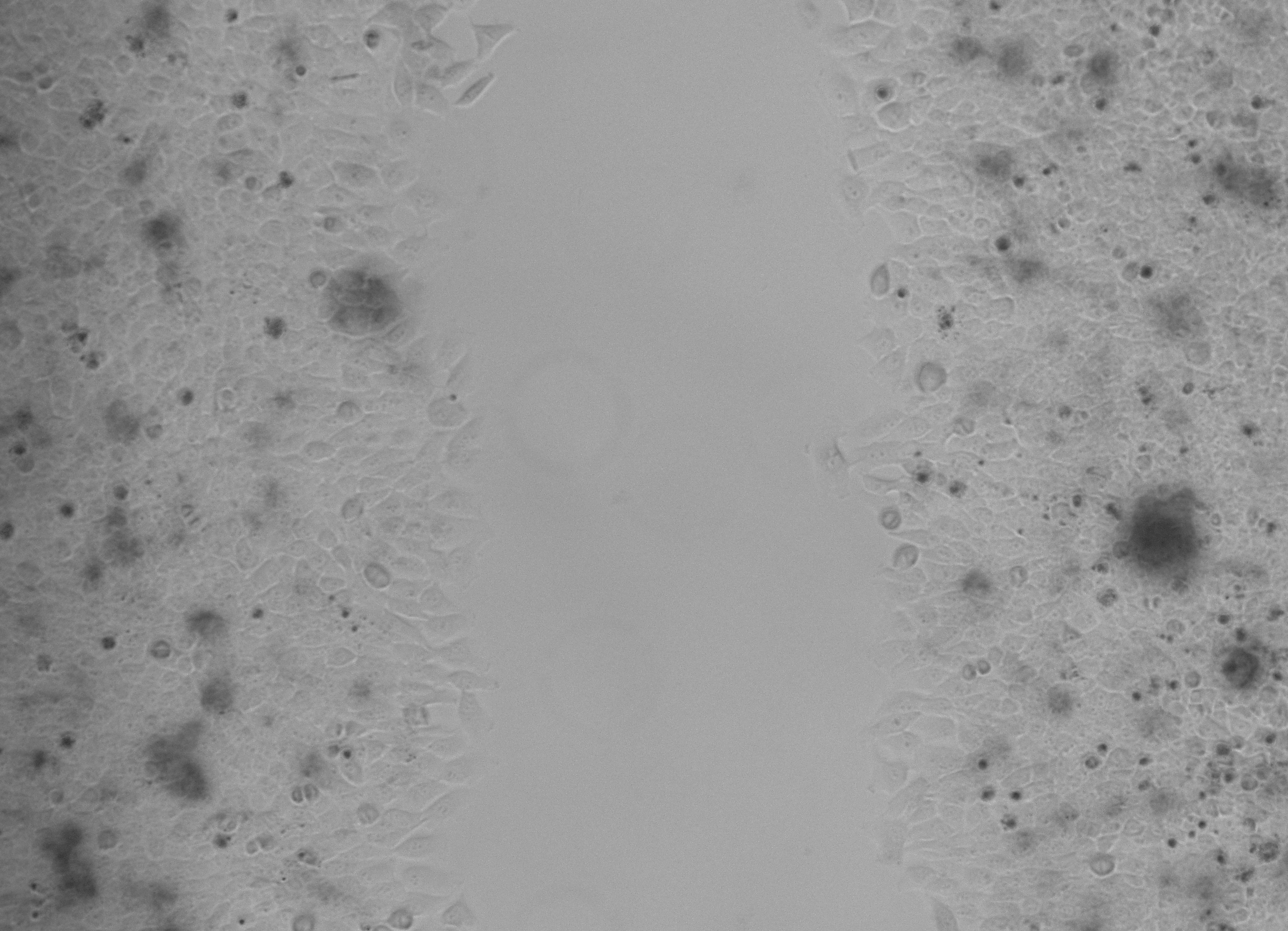

Supplement: Supplementary file 8 — Source data Fig. 6 [file 44319_2025_661_MOESM8_ESM.zip › Figure 6/Figure 6C/48h-12.5.png]

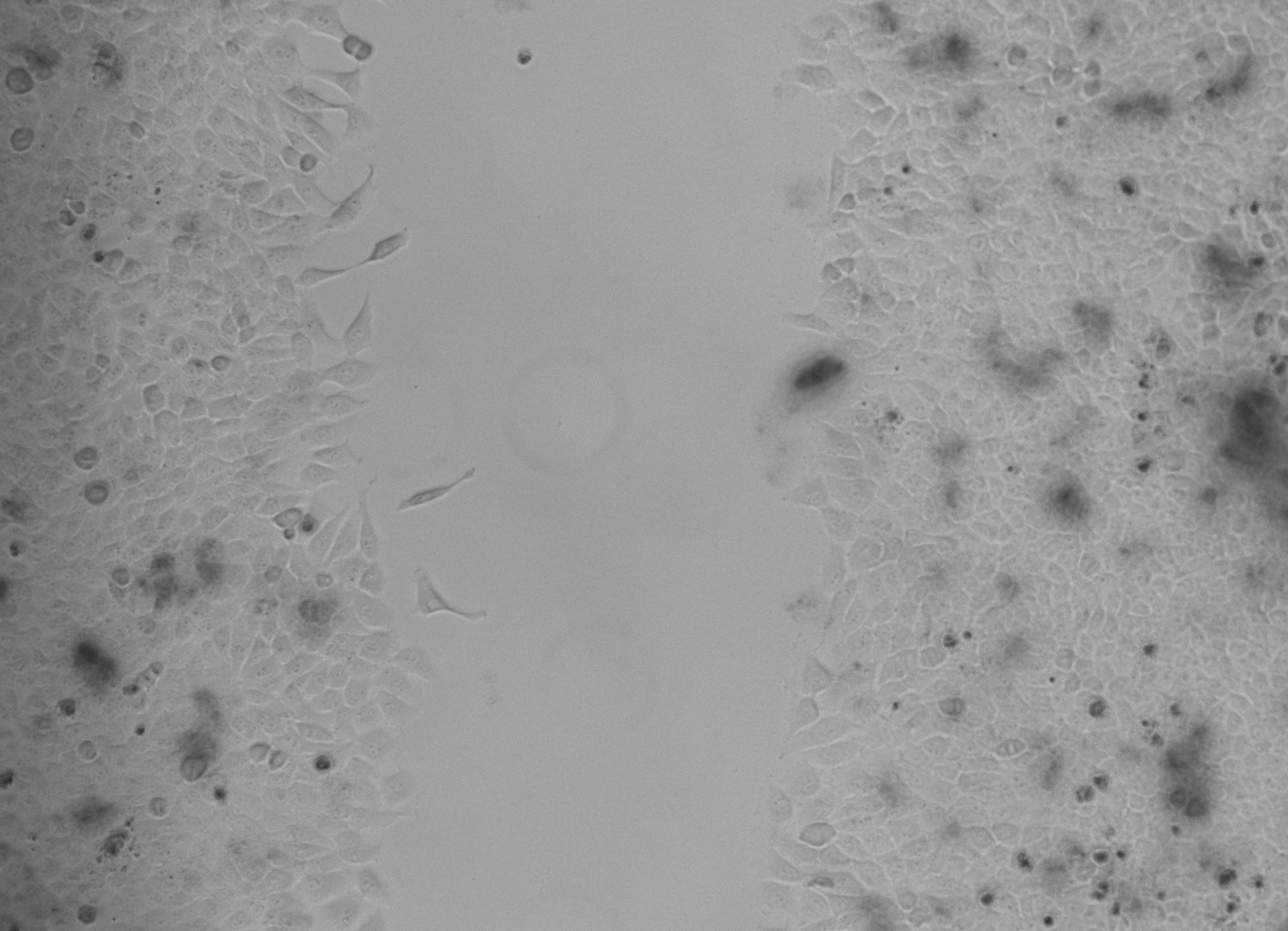

Supplement: Supplementary file 8 — Source data Fig. 6 [file 44319_2025_661_MOESM8_ESM.zip › Figure 6/Figure 6C/48h-25.png]

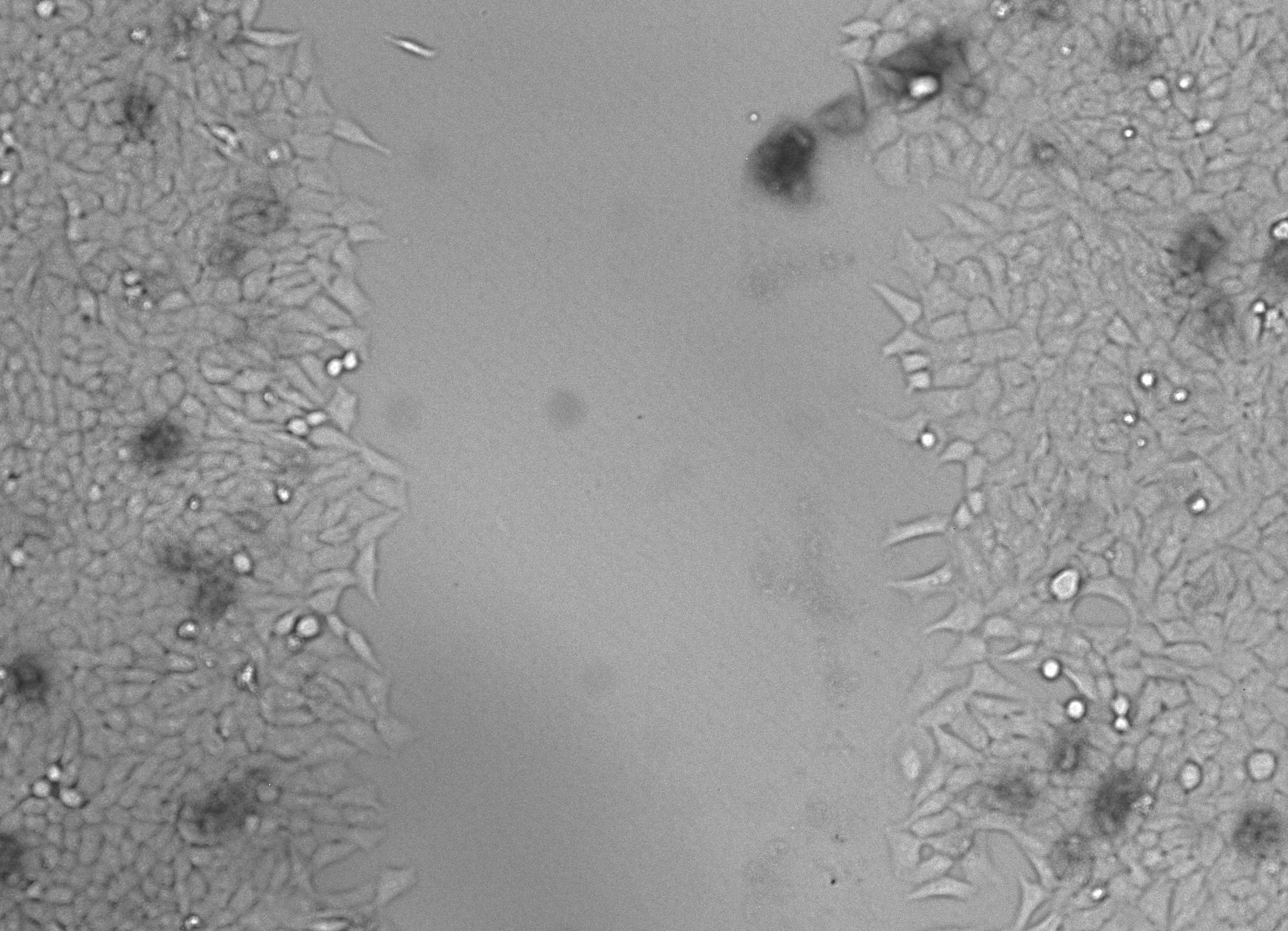

Supplement: Supplementary file 8 — Source data Fig. 6 [file 44319_2025_661_MOESM8_ESM.zip › Figure 6/Figure 6C/48h-50.png]

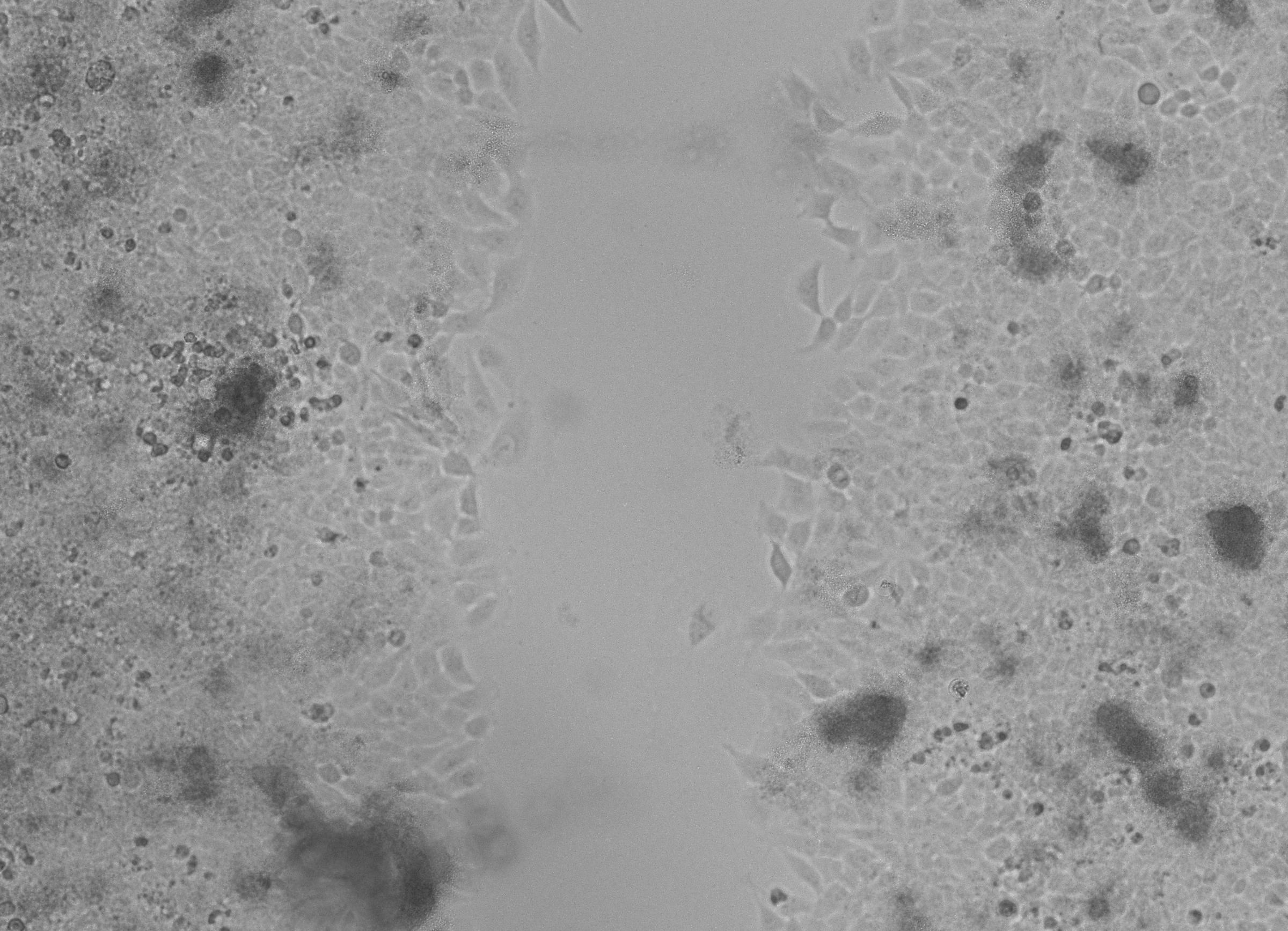

Supplement: Supplementary file 8 — Source data Fig. 6 [file 44319_2025_661_MOESM8_ESM.zip › Figure 6/Figure 6C/48h-Control.png]

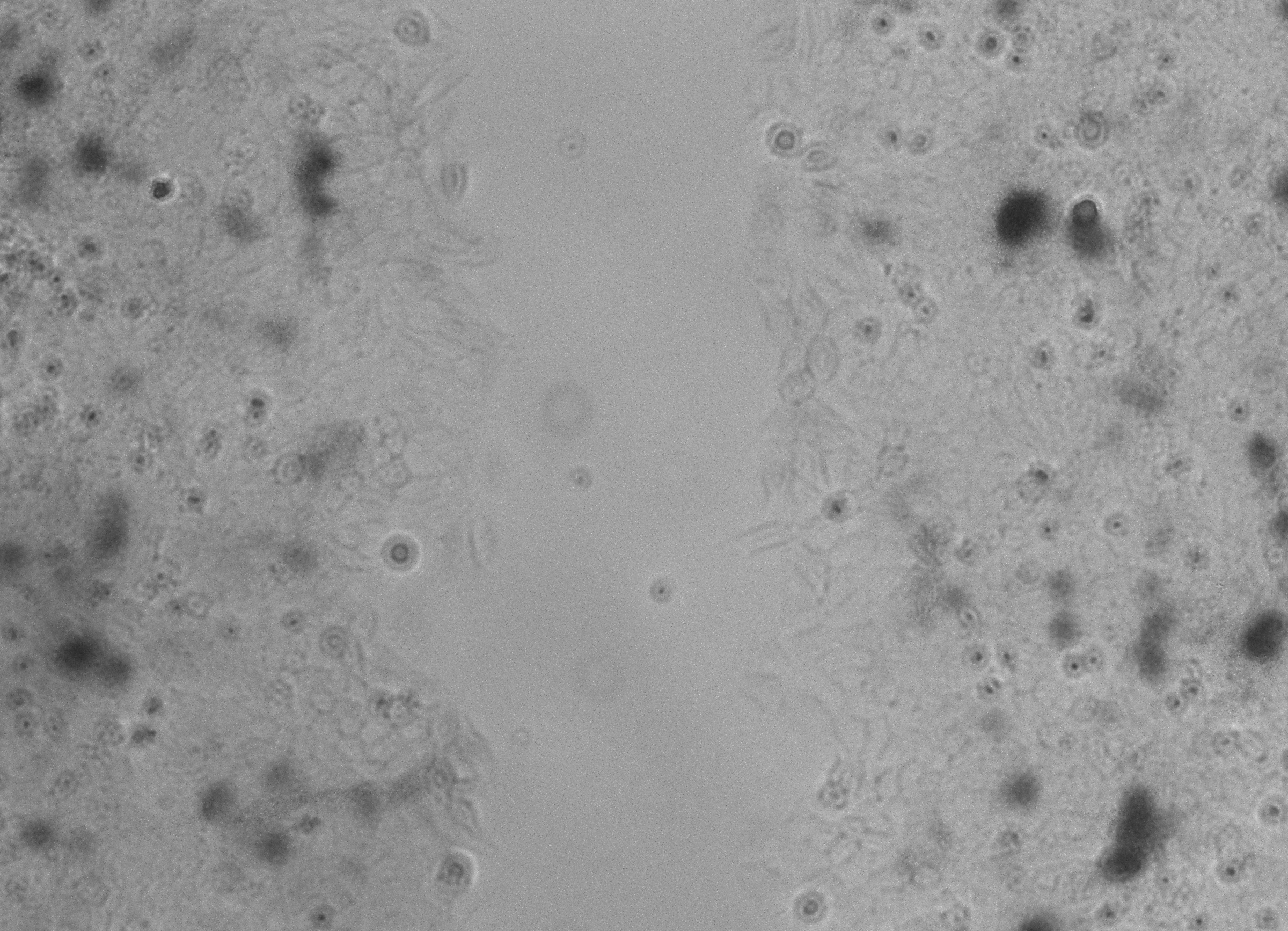

Supplement: Supplementary file 8 — Source data Fig. 6 [file 44319_2025_661_MOESM8_ESM.zip › Figure 6/Figure 6C/72h-12.5.png]

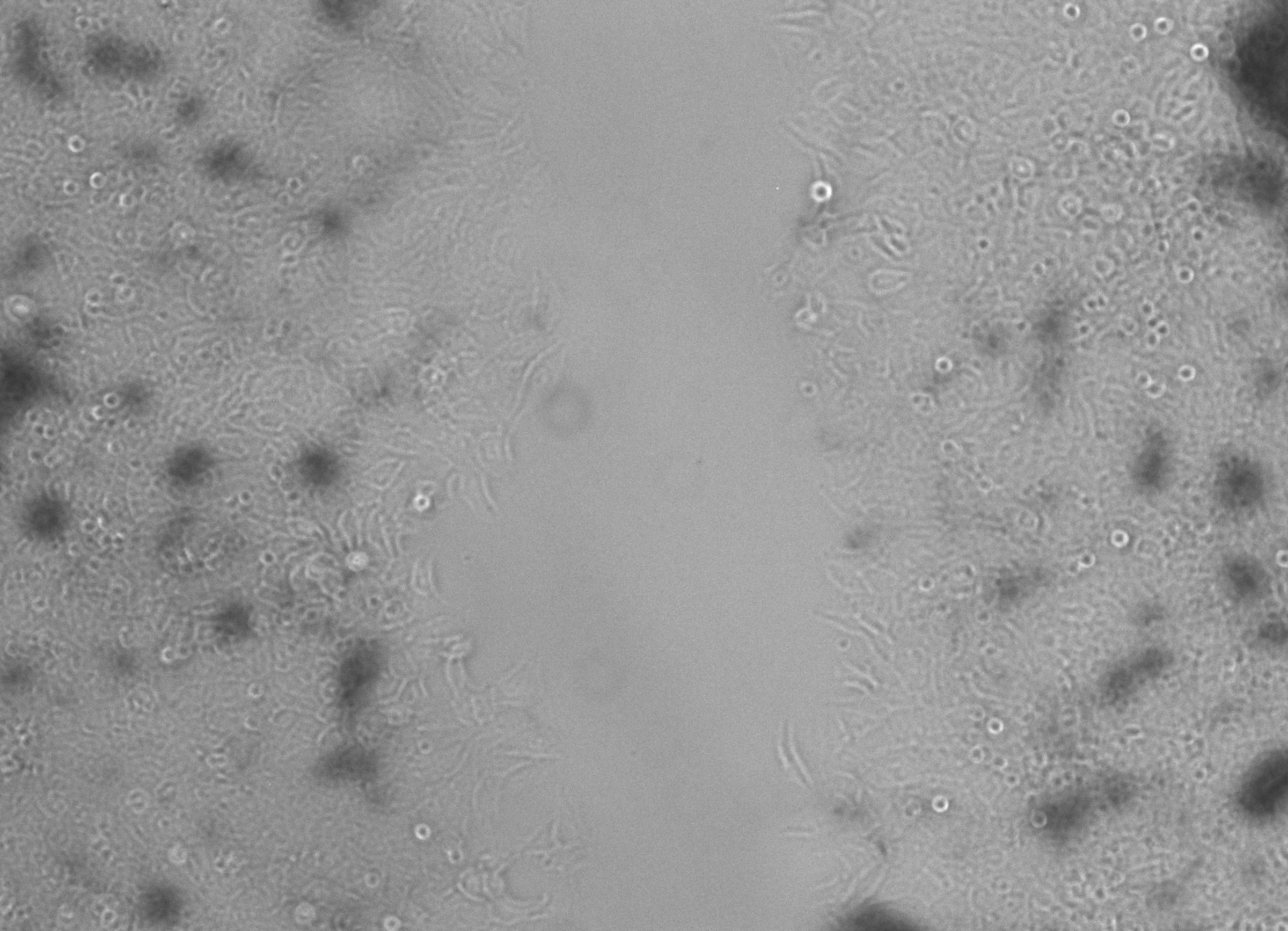

Supplement: Supplementary file 8 — Source data Fig. 6 [file 44319_2025_661_MOESM8_ESM.zip › Figure 6/Figure 6C/72h-25.png]

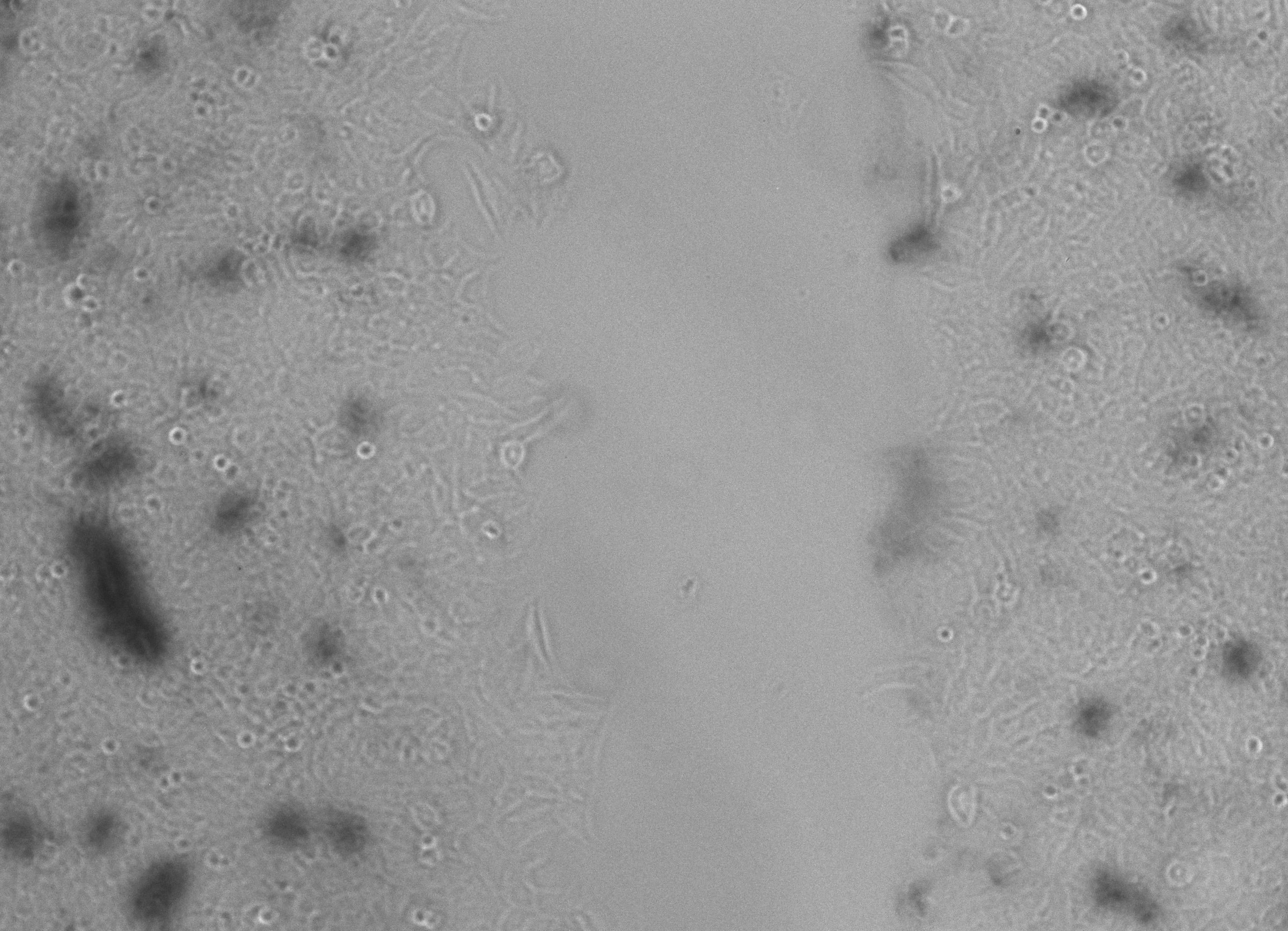

Supplement: Supplementary file 8 — Source data Fig. 6 [file 44319_2025_661_MOESM8_ESM.zip › Figure 6/Figure 6C/72h-50.png]

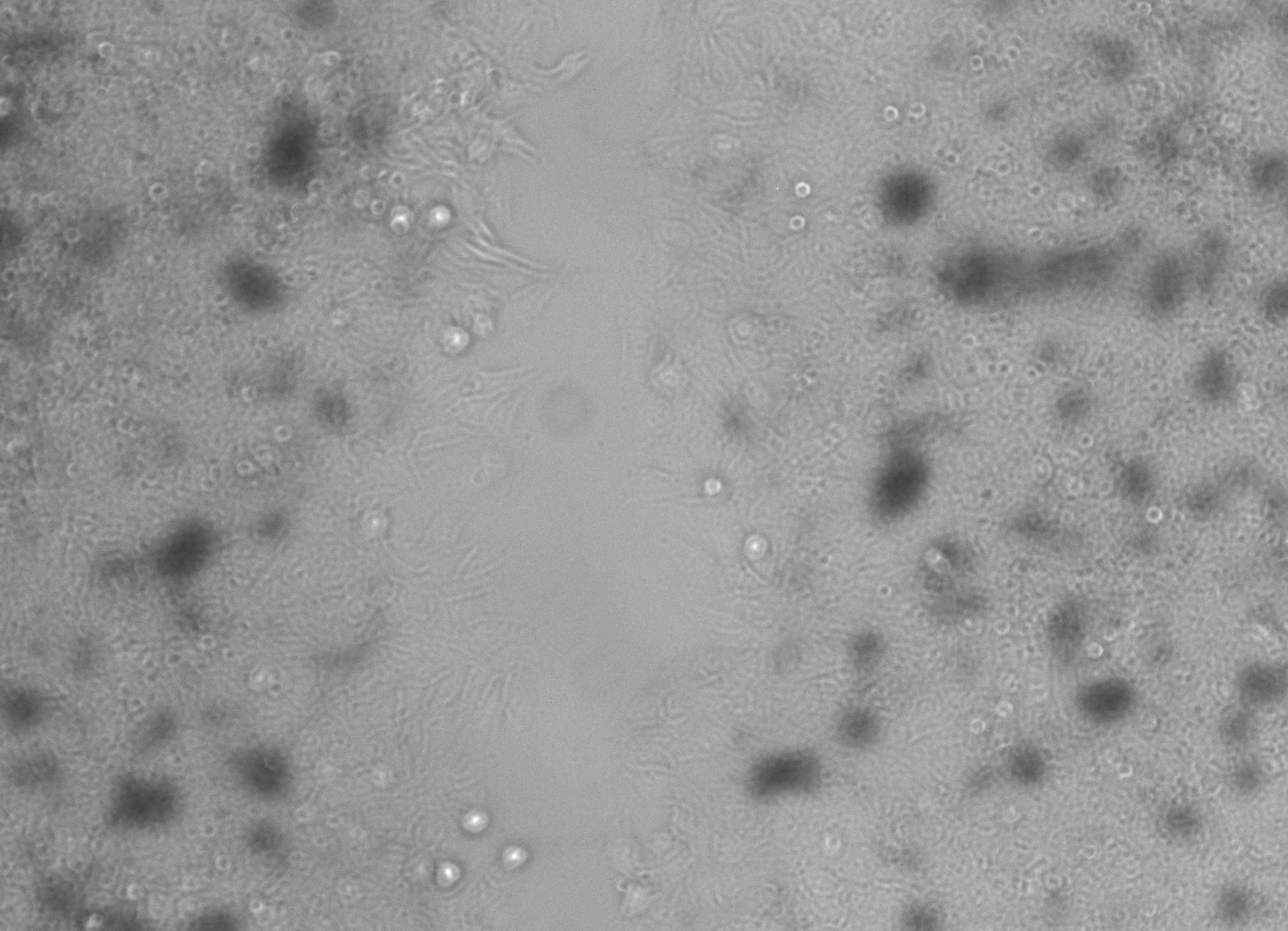

Supplement: Supplementary file 8 — Source data Fig. 6 [file 44319_2025_661_MOESM8_ESM.zip › Figure 6/Figure 6C/72h-Control.png]

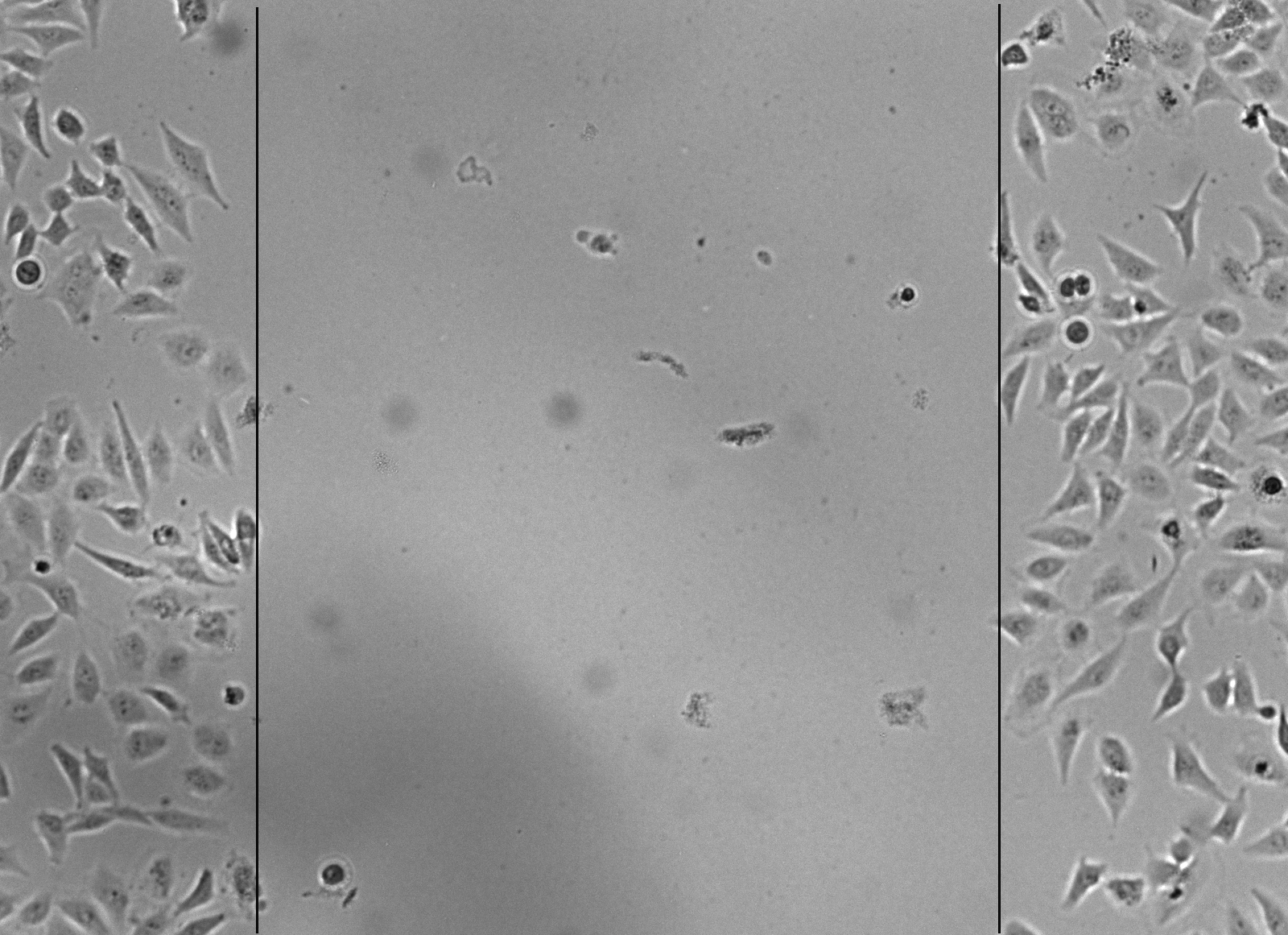

Supplement: Supplementary file 8 — Source data Fig. 6 [file 44319_2025_661_MOESM8_ESM.zip › Figure 6/Figure 6E/sh-MCT1+Tuc-0h.png]

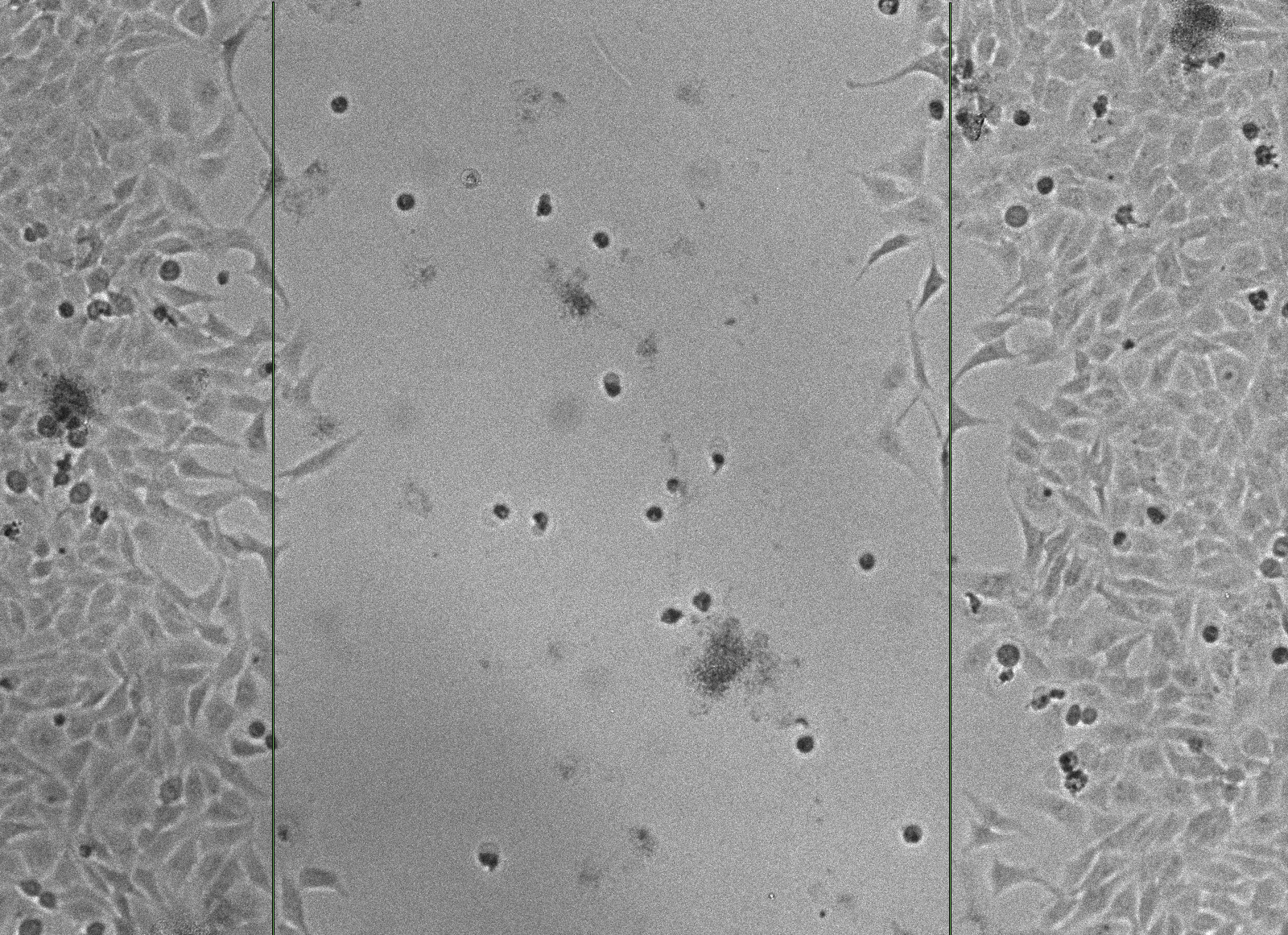

Supplement: Supplementary file 8 — Source data Fig. 6 [file 44319_2025_661_MOESM8_ESM.zip › Figure 6/Figure 6E/sh-MCT1+Tuc-24h.png]

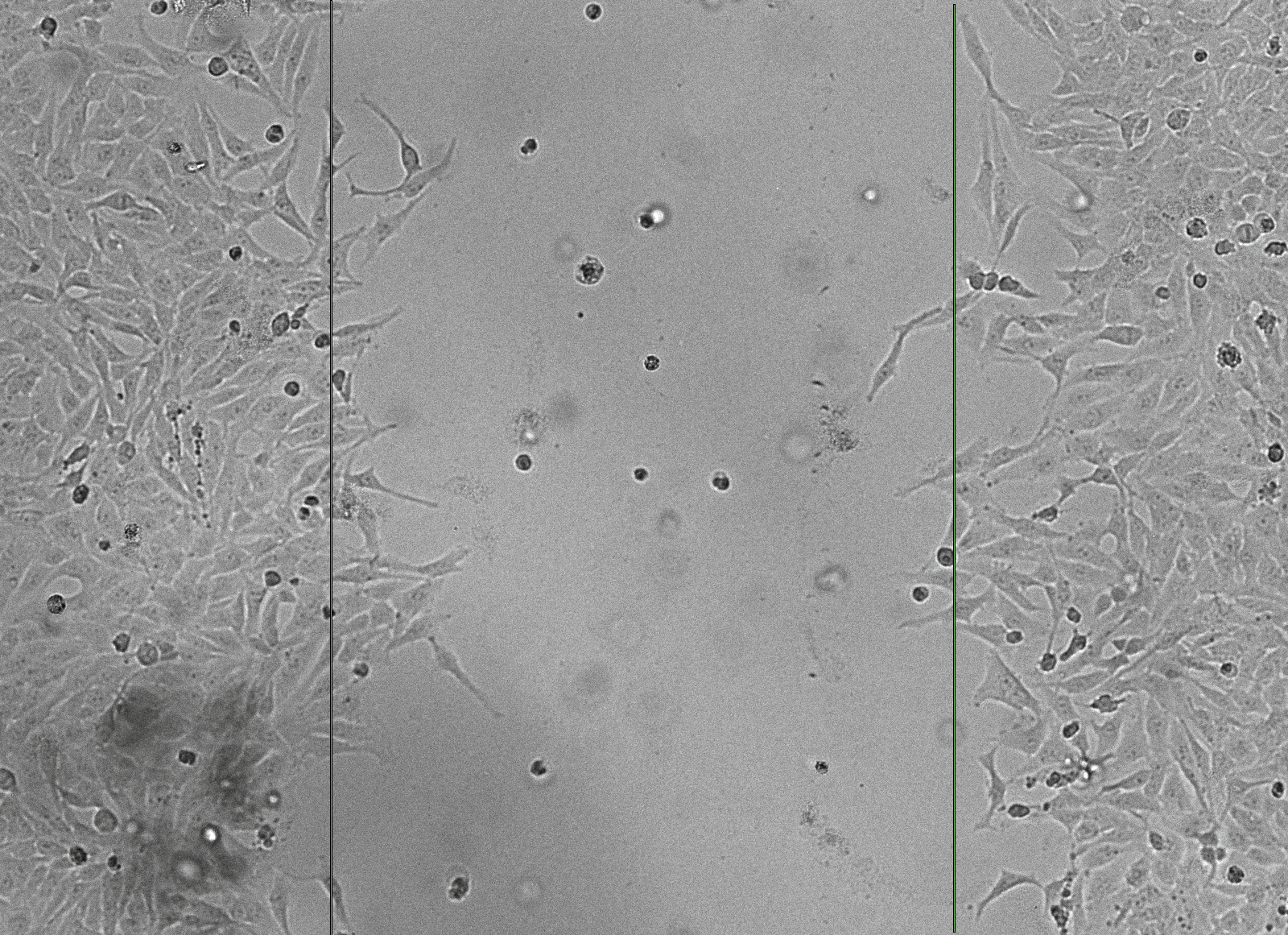

Supplement: Supplementary file 8 — Source data Fig. 6 [file 44319_2025_661_MOESM8_ESM.zip › Figure 6/Figure 6E/sh-MCT1+Tuc-48h.png]

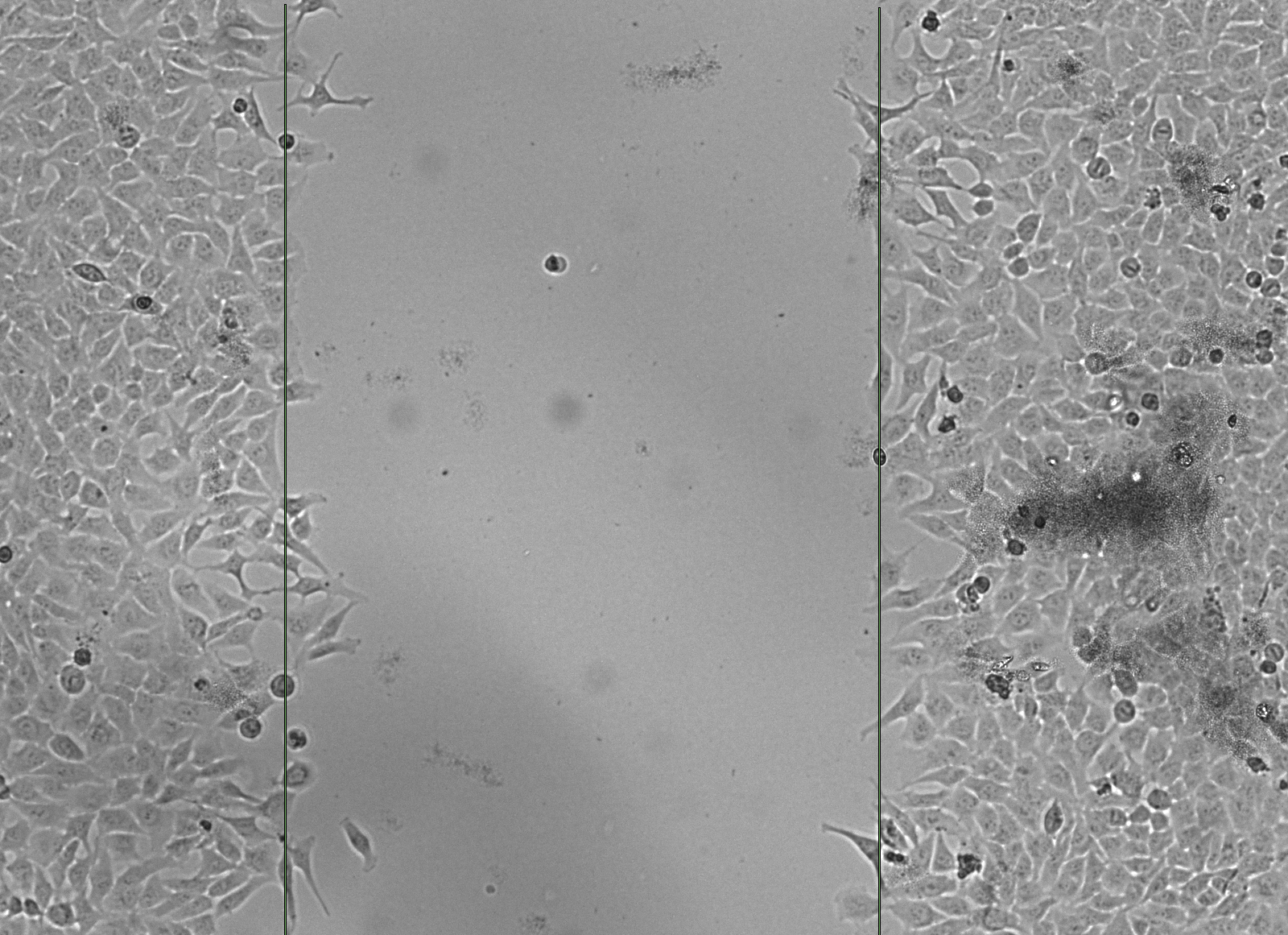

Supplement: Supplementary file 8 — Source data Fig. 6 [file 44319_2025_661_MOESM8_ESM.zip › Figure 6/Figure 6E/sh-MCT1+Tuc-72h.png]

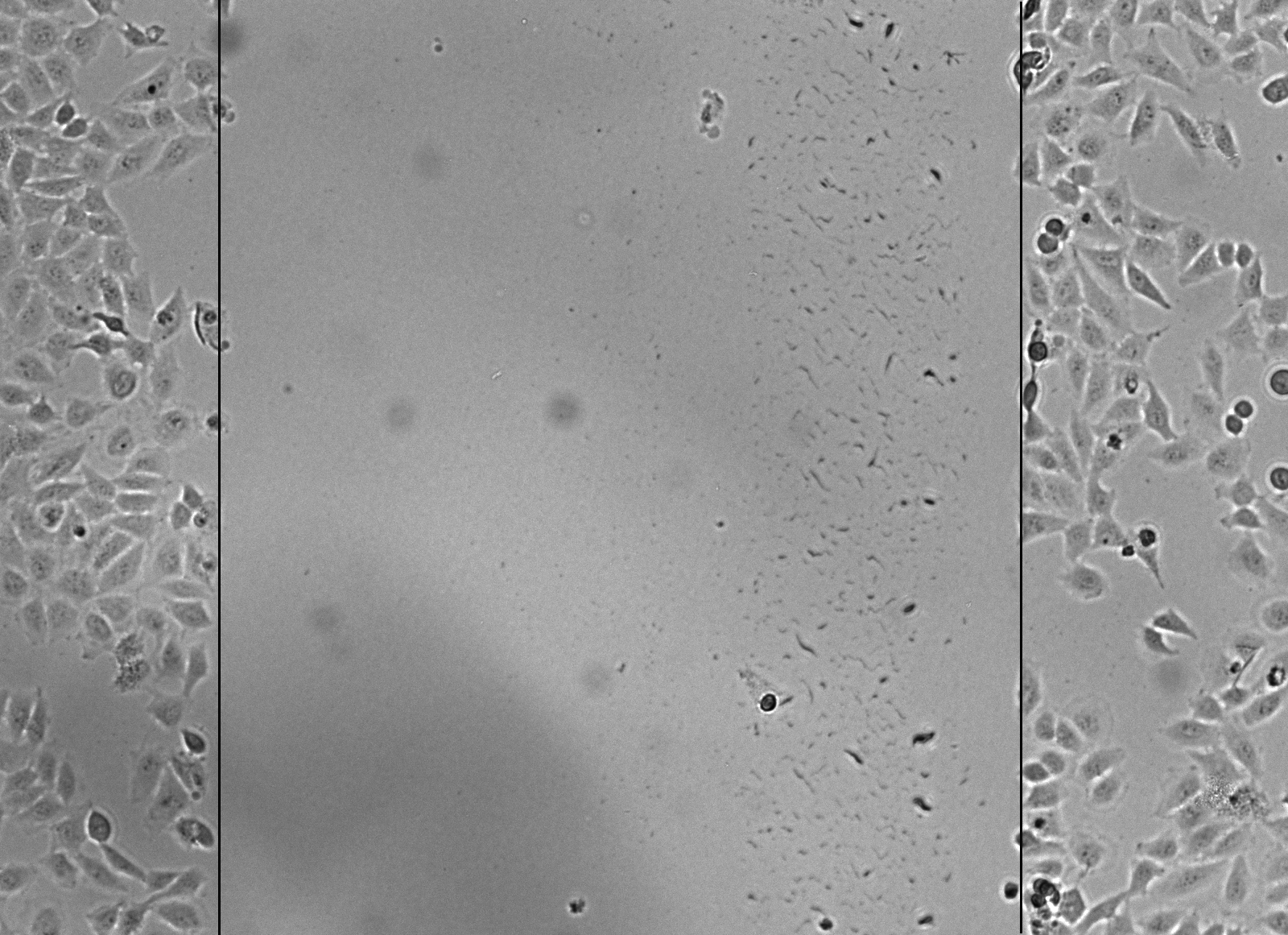

Supplement: Supplementary file 8 — Source data Fig. 6 [file 44319_2025_661_MOESM8_ESM.zip › Figure 6/Figure 6E/sh-MCT1-0h.png]

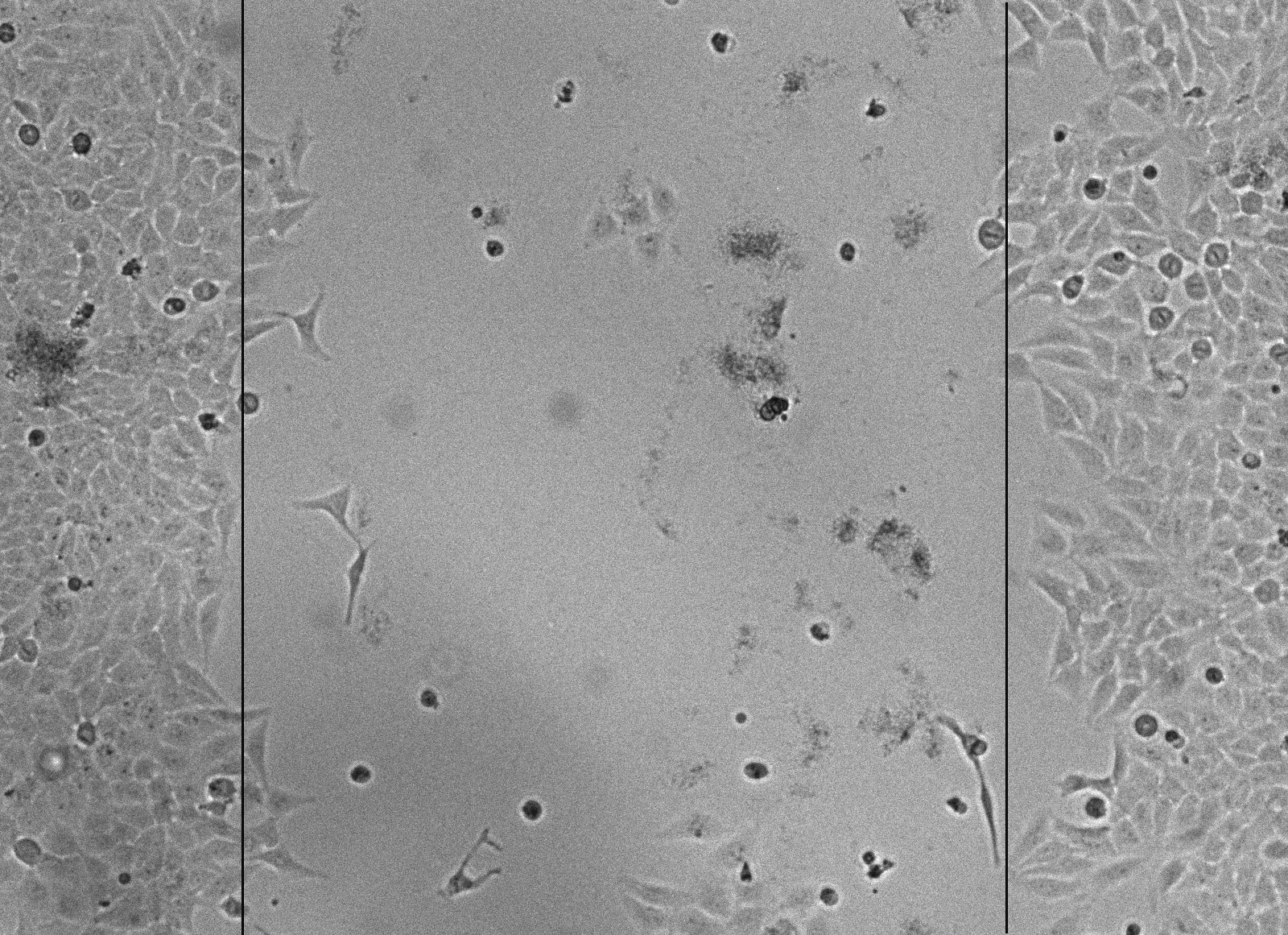

Supplement: Supplementary file 8 — Source data Fig. 6 [file 44319_2025_661_MOESM8_ESM.zip › Figure 6/Figure 6E/sh-MCT1-24h.png]

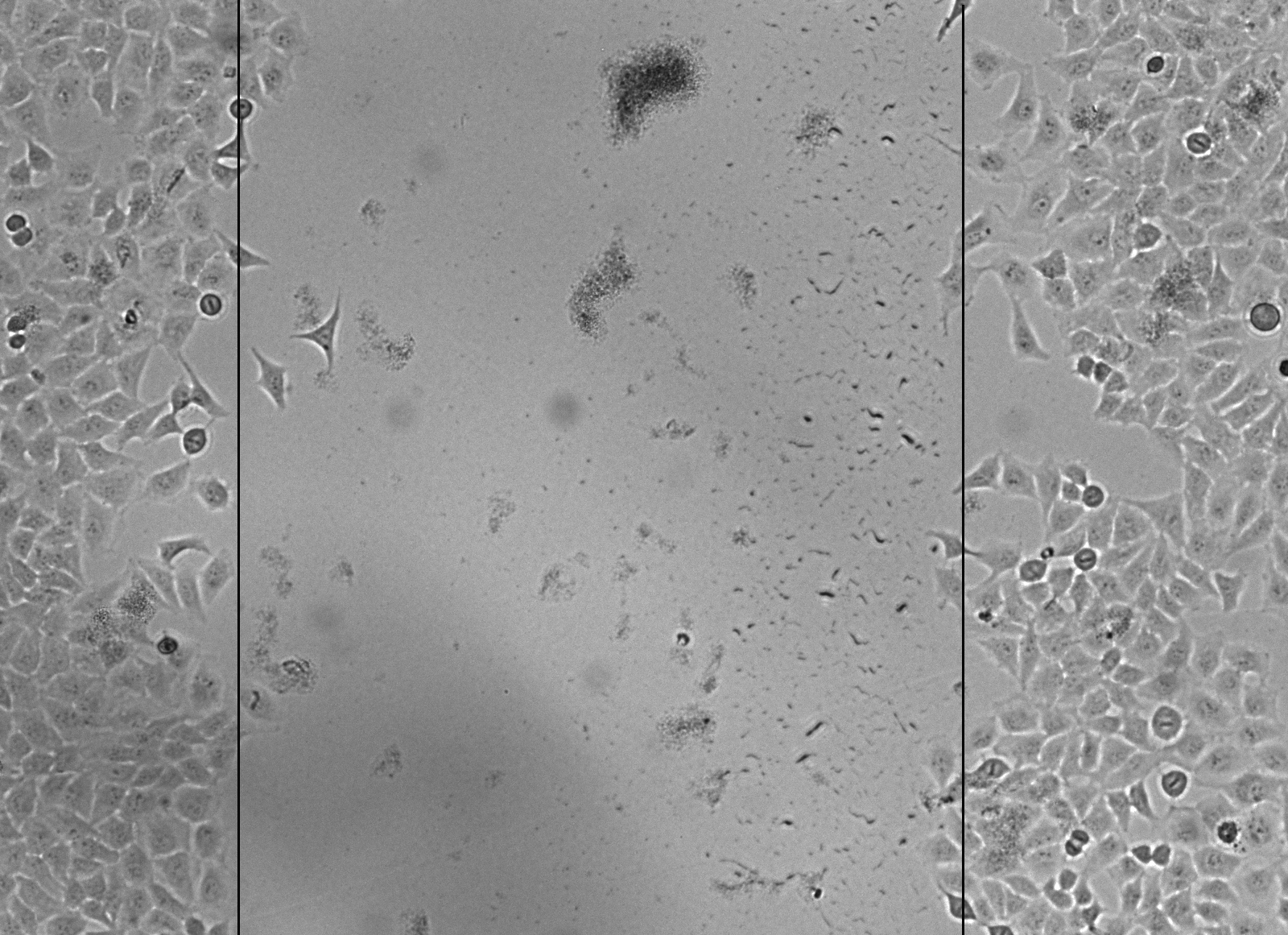

Supplement: Supplementary file 8 — Source data Fig. 6 [file 44319_2025_661_MOESM8_ESM.zip › Figure 6/Figure 6E/sh-MCT1-48h.png]

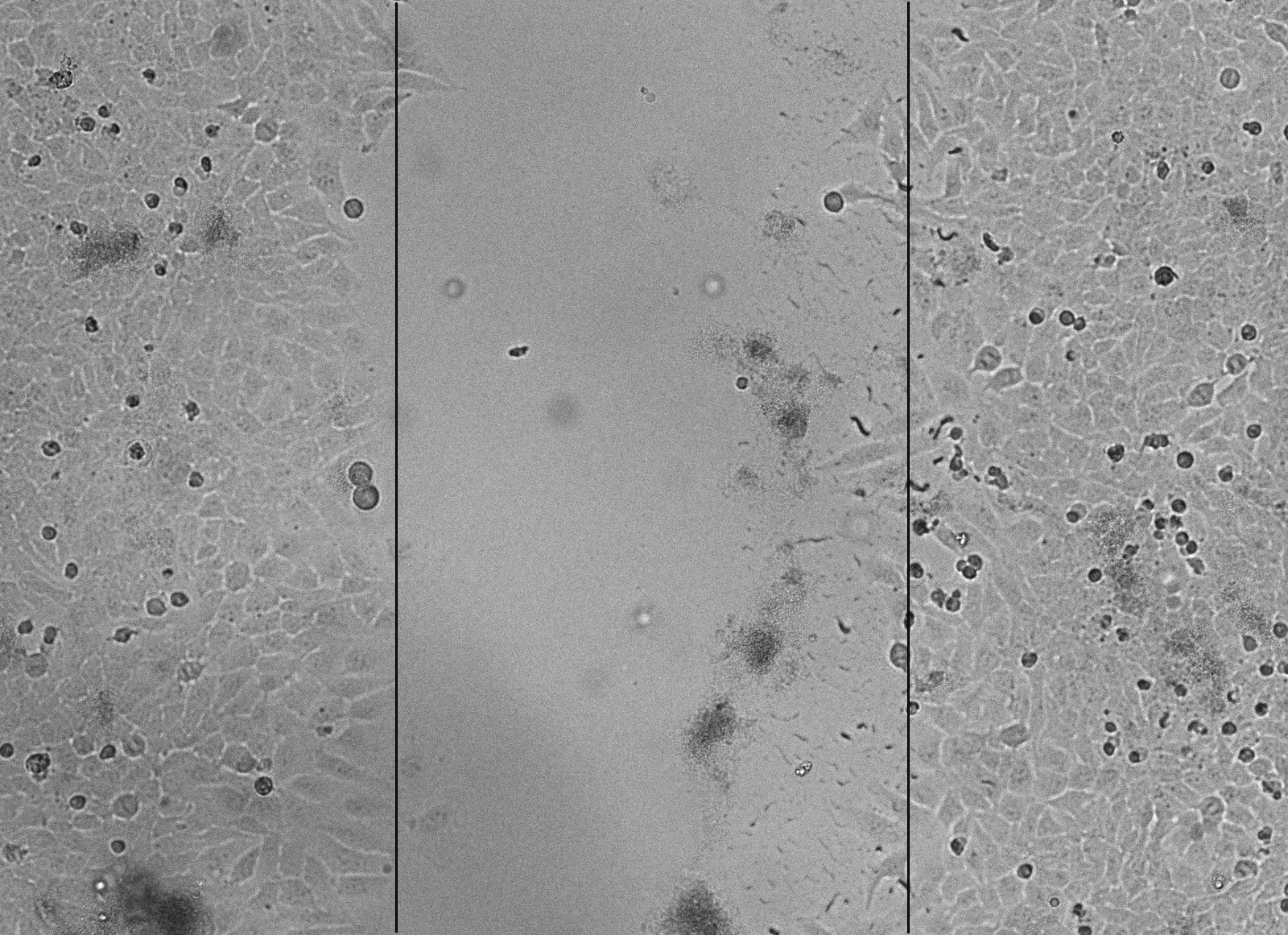

Supplement: Supplementary file 8 — Source data Fig. 6 [file 44319_2025_661_MOESM8_ESM.zip › Figure 6/Figure 6E/sh-MCT1-72h.png]

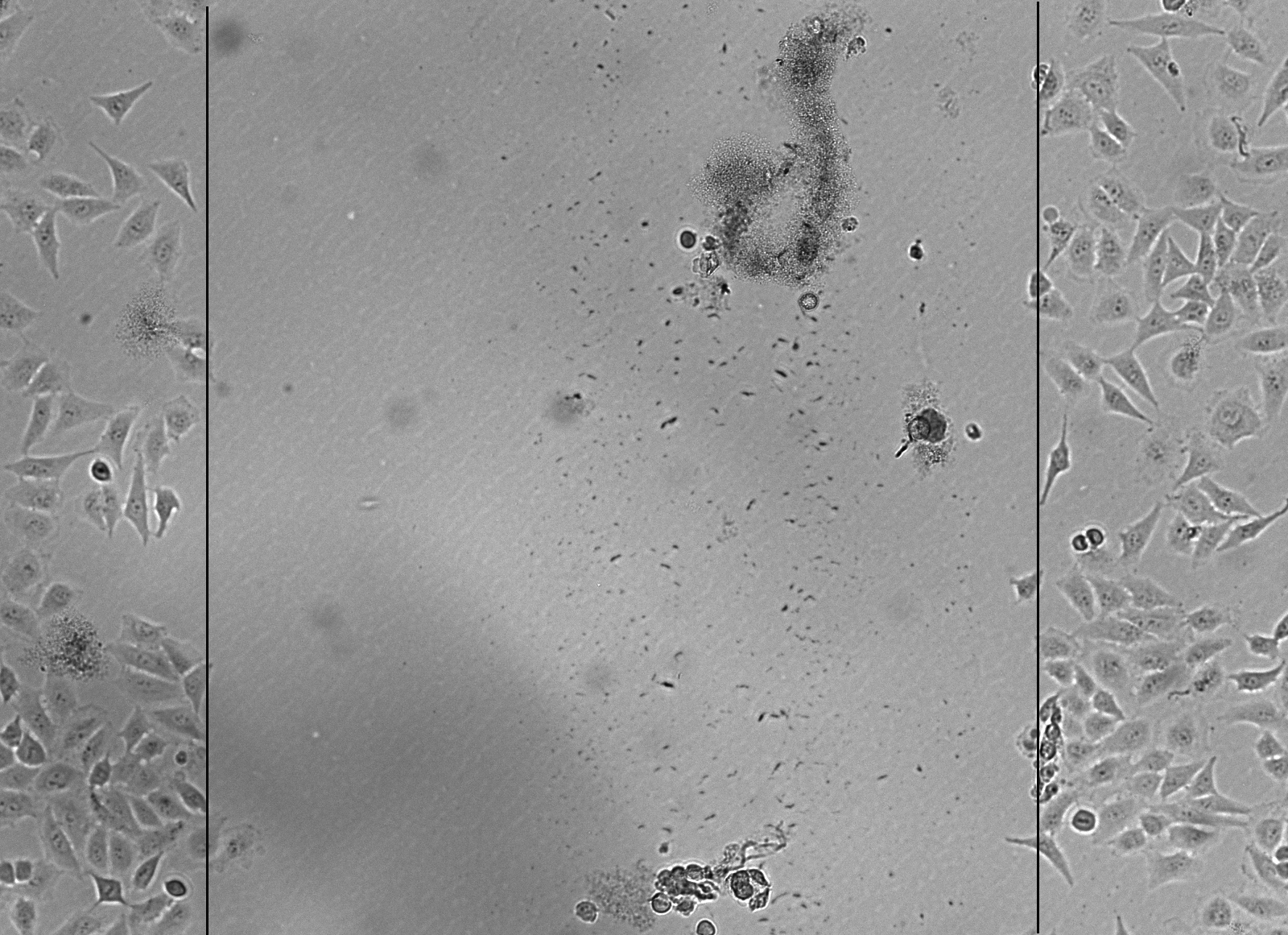

Supplement: Supplementary file 8 — Source data Fig. 6 [file 44319_2025_661_MOESM8_ESM.zip › Figure 6/Figure 6E/sh-NC+Tuc-0h.png]

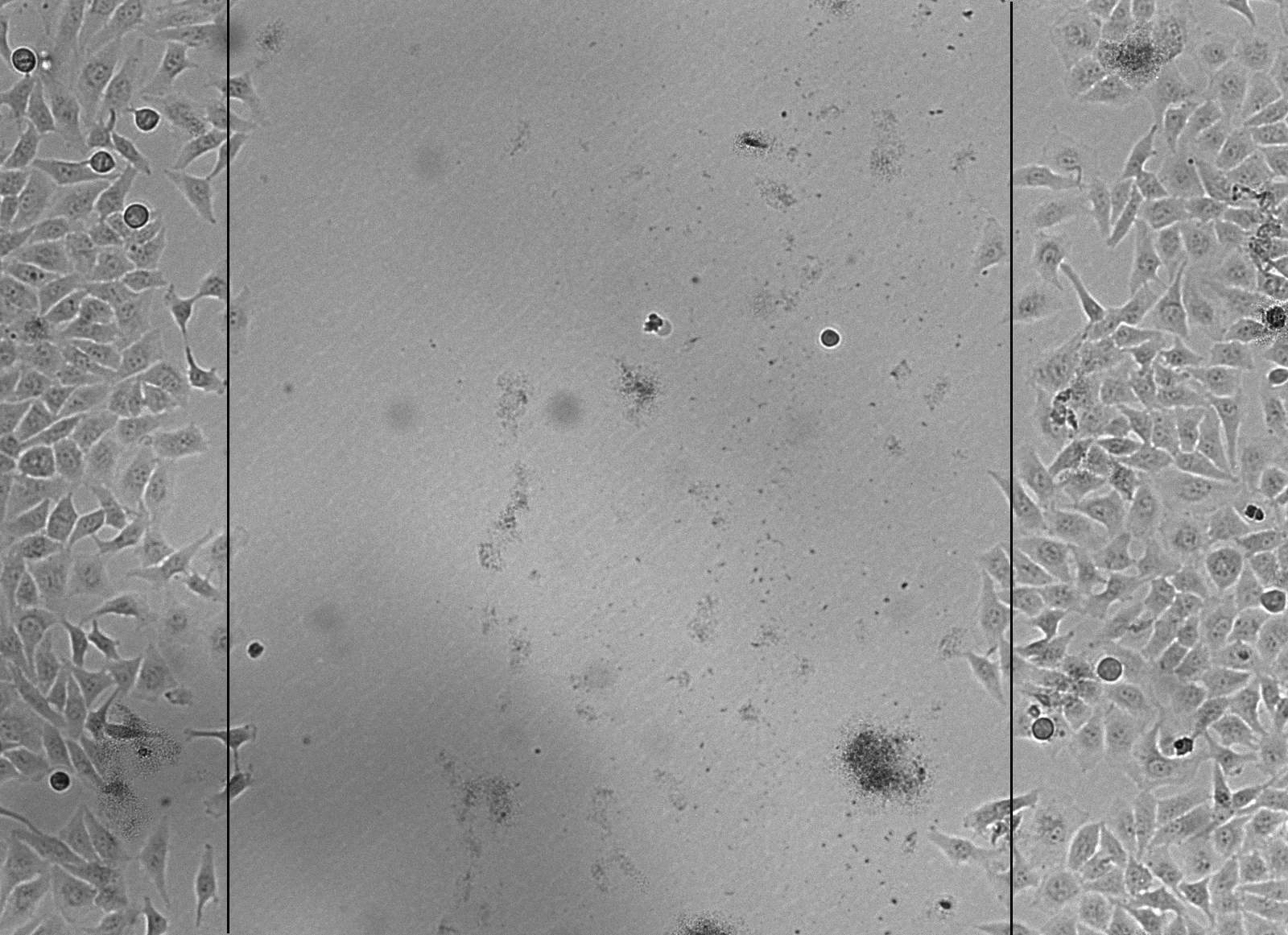

Supplement: Supplementary file 8 — Source data Fig. 6 [file 44319_2025_661_MOESM8_ESM.zip › Figure 6/Figure 6E/sh-NC+Tuc-24h.png]

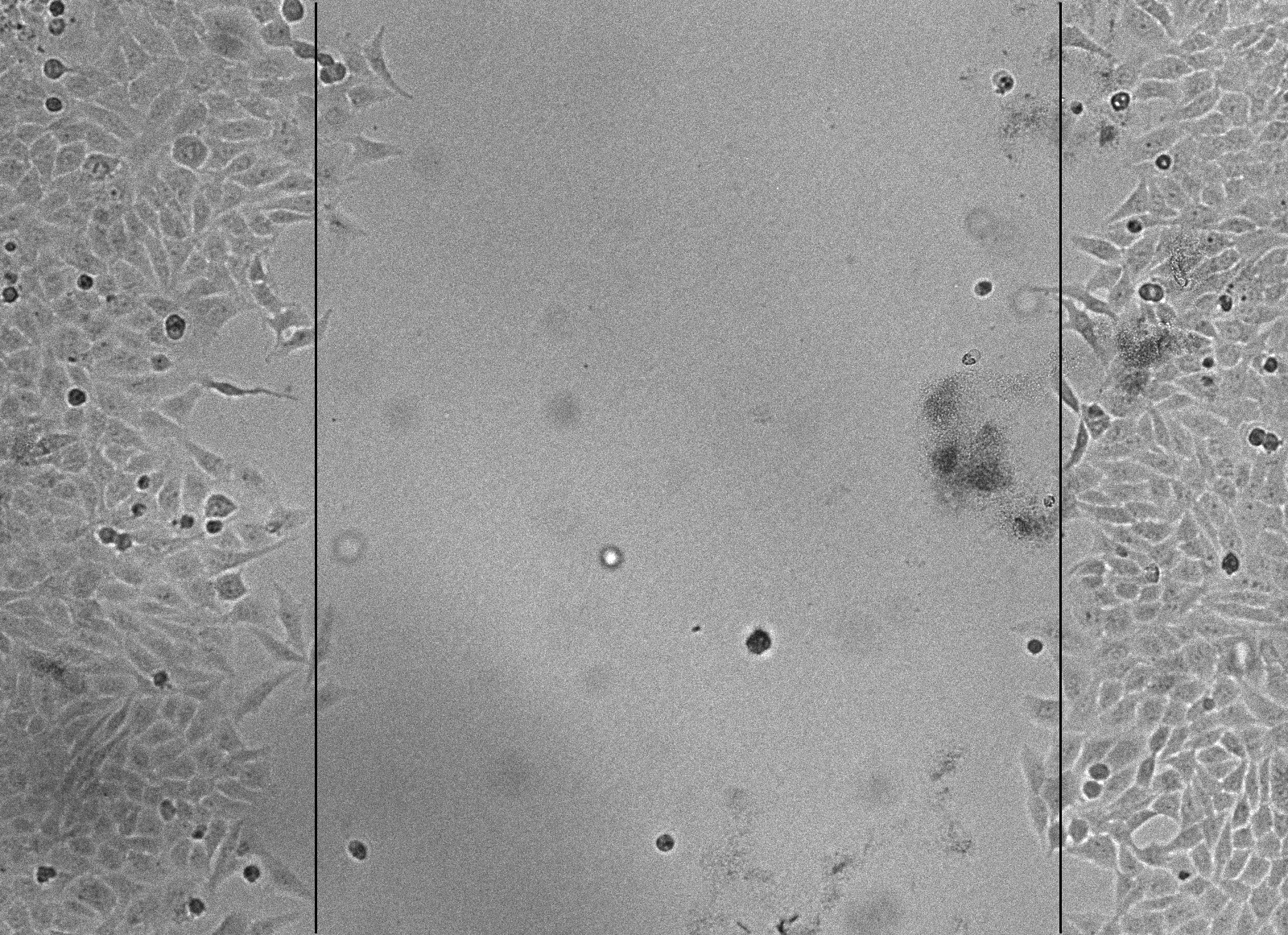

Supplement: Supplementary file 8 — Source data Fig. 6 [file 44319_2025_661_MOESM8_ESM.zip › Figure 6/Figure 6E/sh-NC+Tuc-48h.png]

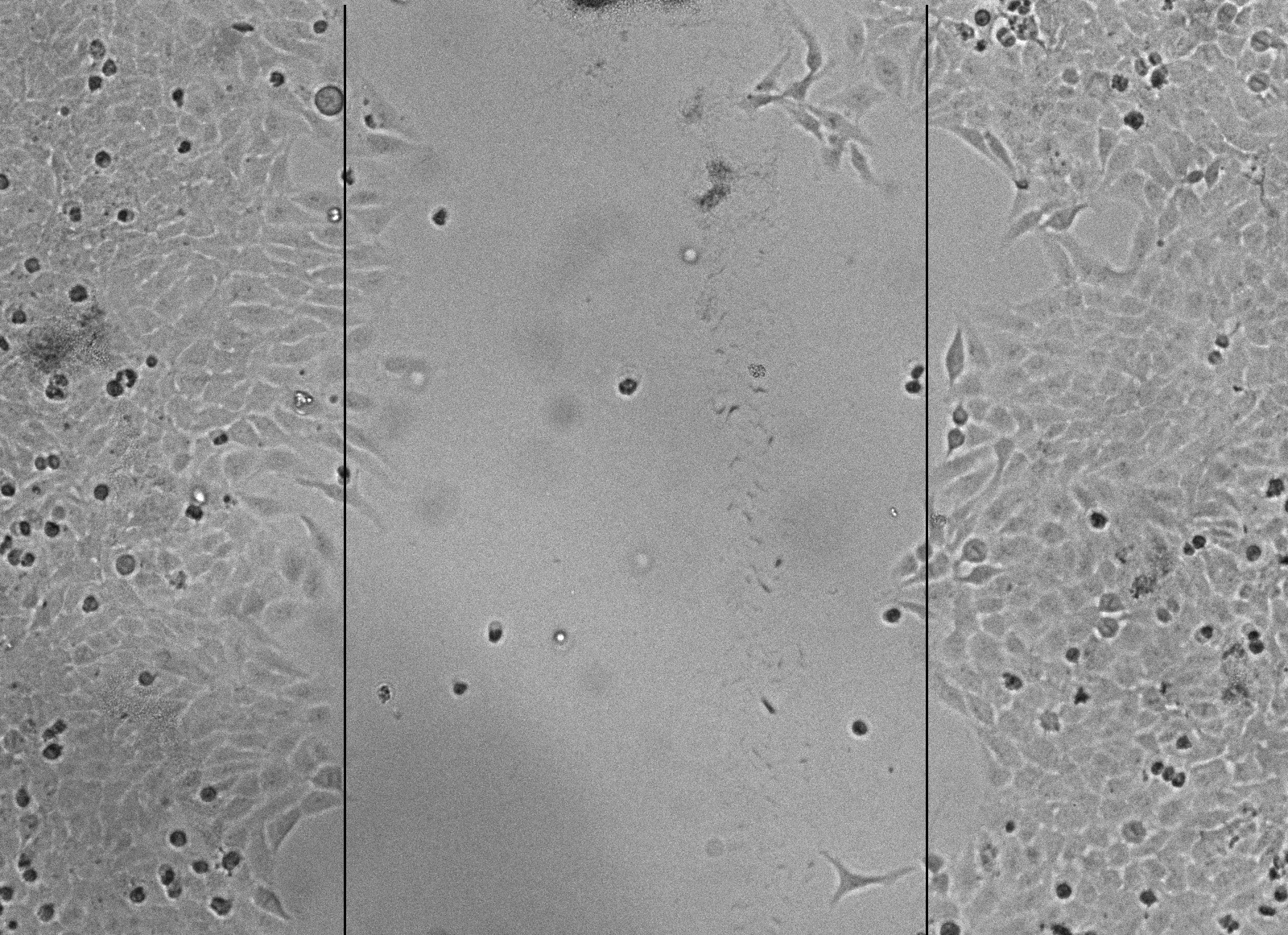

Supplement: Supplementary file 8 — Source data Fig. 6 [file 44319_2025_661_MOESM8_ESM.zip › Figure 6/Figure 6E/sh-NC+Tuc-72h.png]

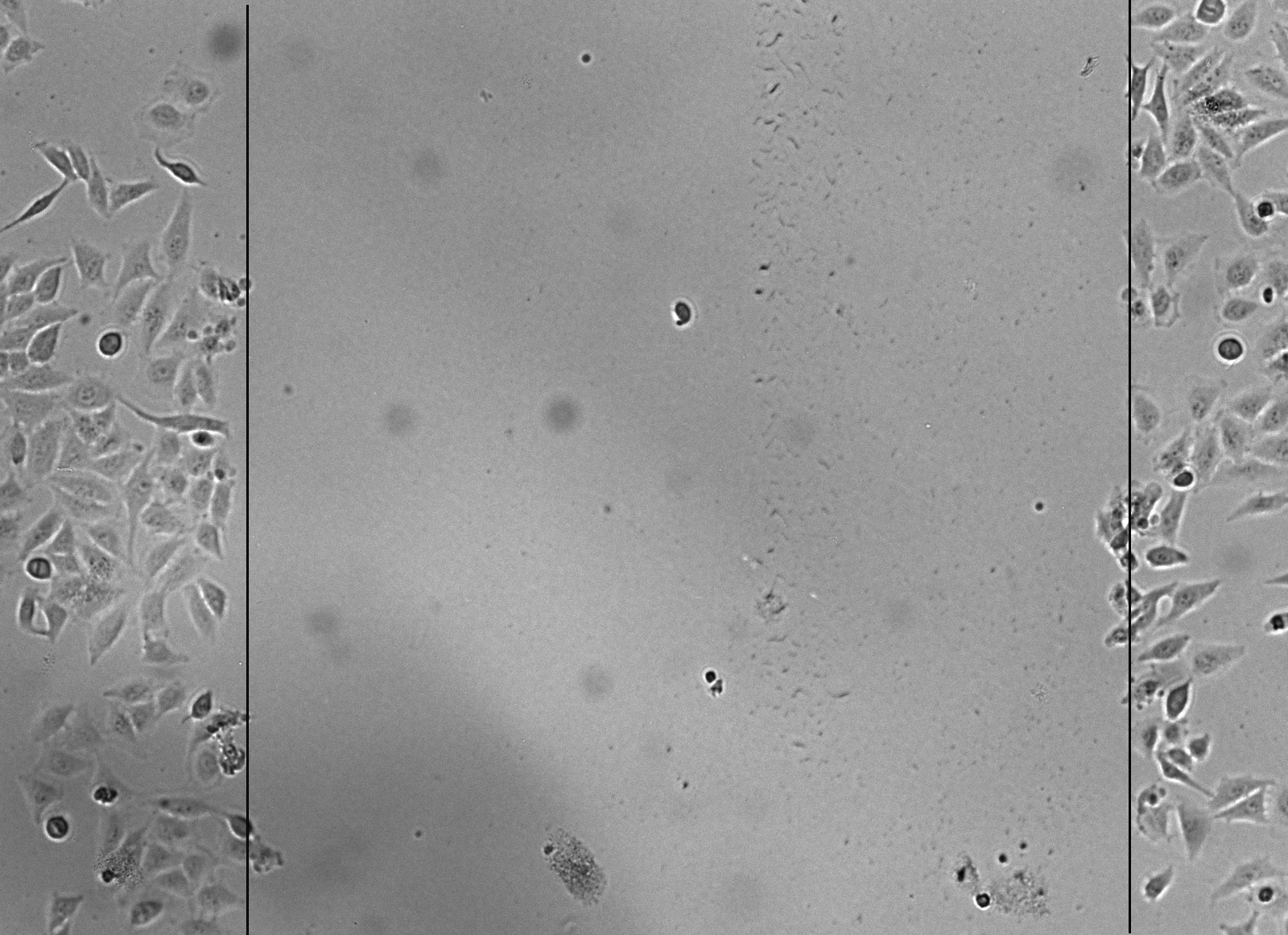

Supplement: Supplementary file 8 — Source data Fig. 6 [file 44319_2025_661_MOESM8_ESM.zip › Figure 6/Figure 6E/sh-NC-0h.png]

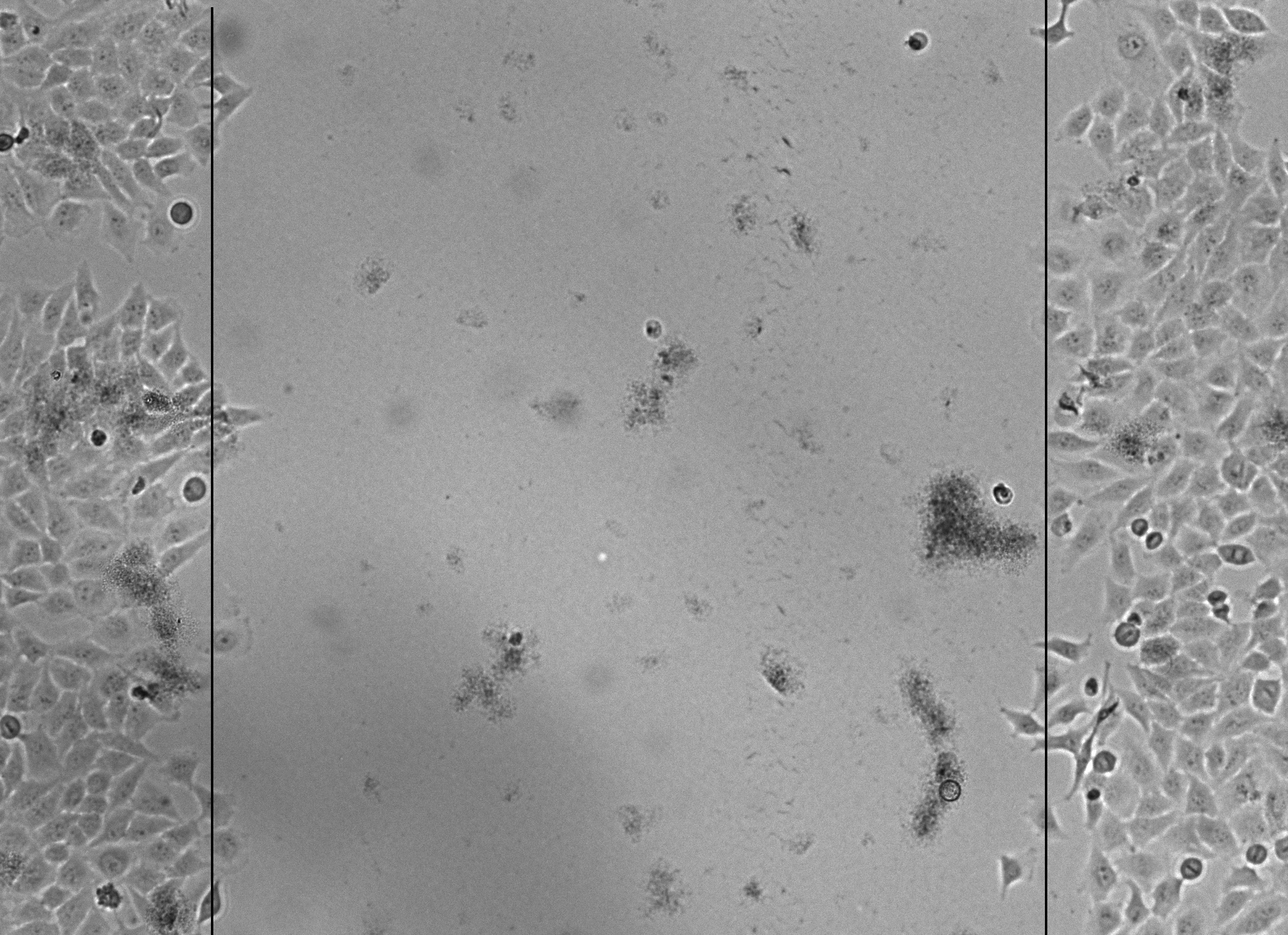

Supplement: Supplementary file 8 — Source data Fig. 6 [file 44319_2025_661_MOESM8_ESM.zip › Figure 6/Figure 6E/sh-NC-24h.png]

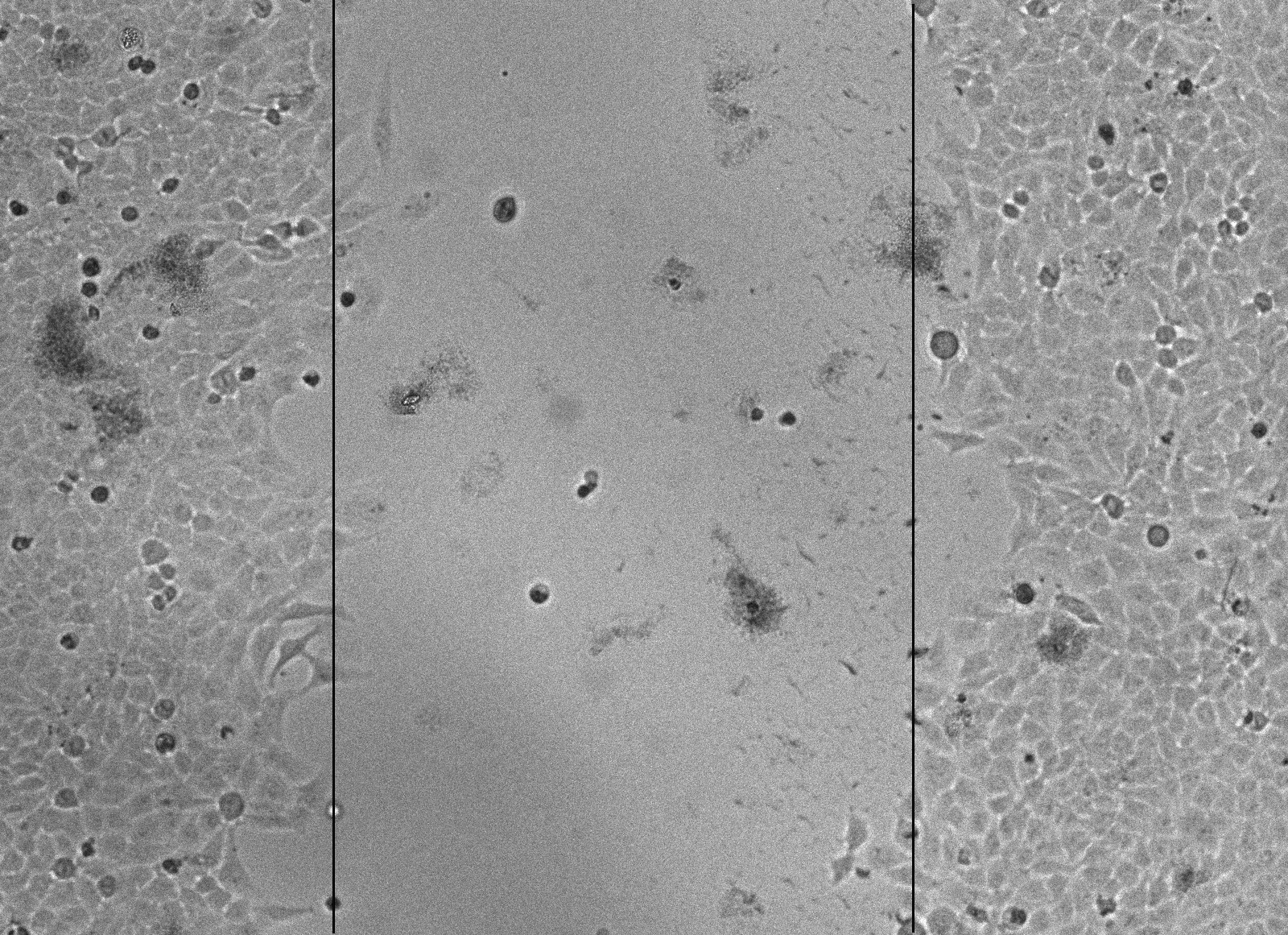

Supplement: Supplementary file 8 — Source data Fig. 6 [file 44319_2025_661_MOESM8_ESM.zip › Figure 6/Figure 6E/sh-NC-48h.png]

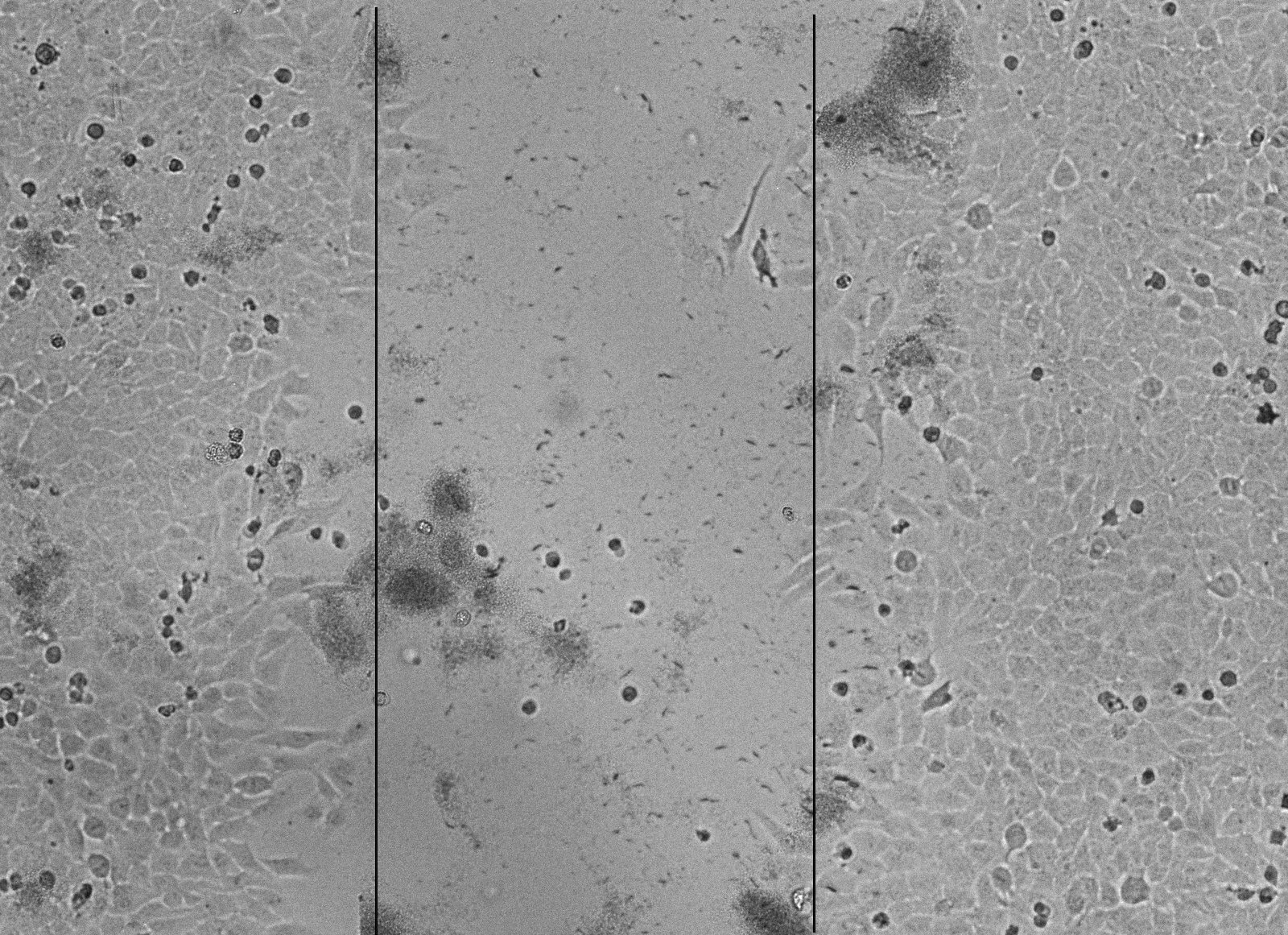

Supplement: Supplementary file 8 — Source data Fig. 6 [file 44319_2025_661_MOESM8_ESM.zip › Figure 6/Figure 6E/sh-NC-72h.png]

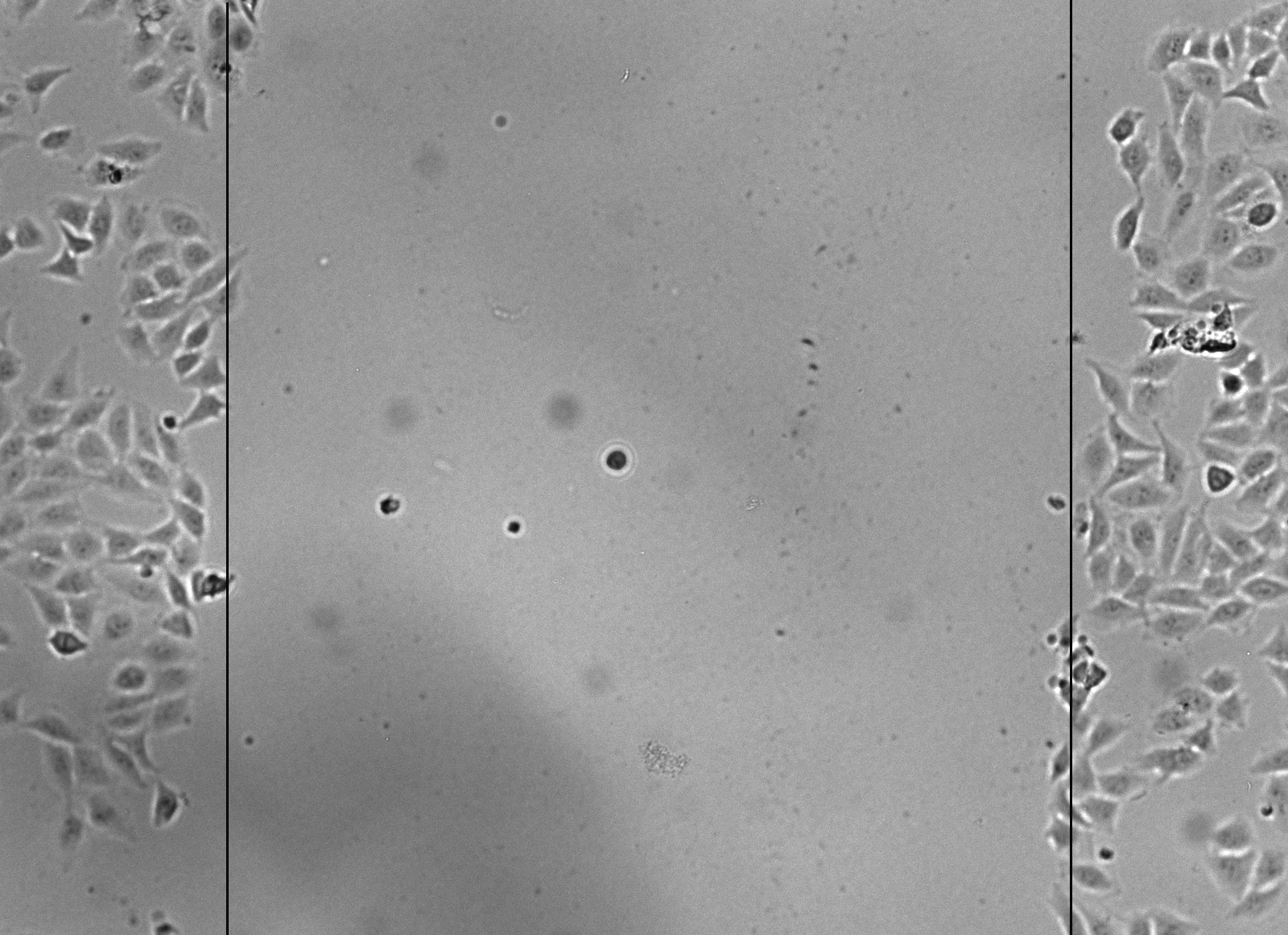

Supplement: Supplementary file 8 — Source data Fig. 6 [file 44319_2025_661_MOESM8_ESM.zip › Figure 6/Figure 6G/sh-MCT2+Tuc-0h.png]

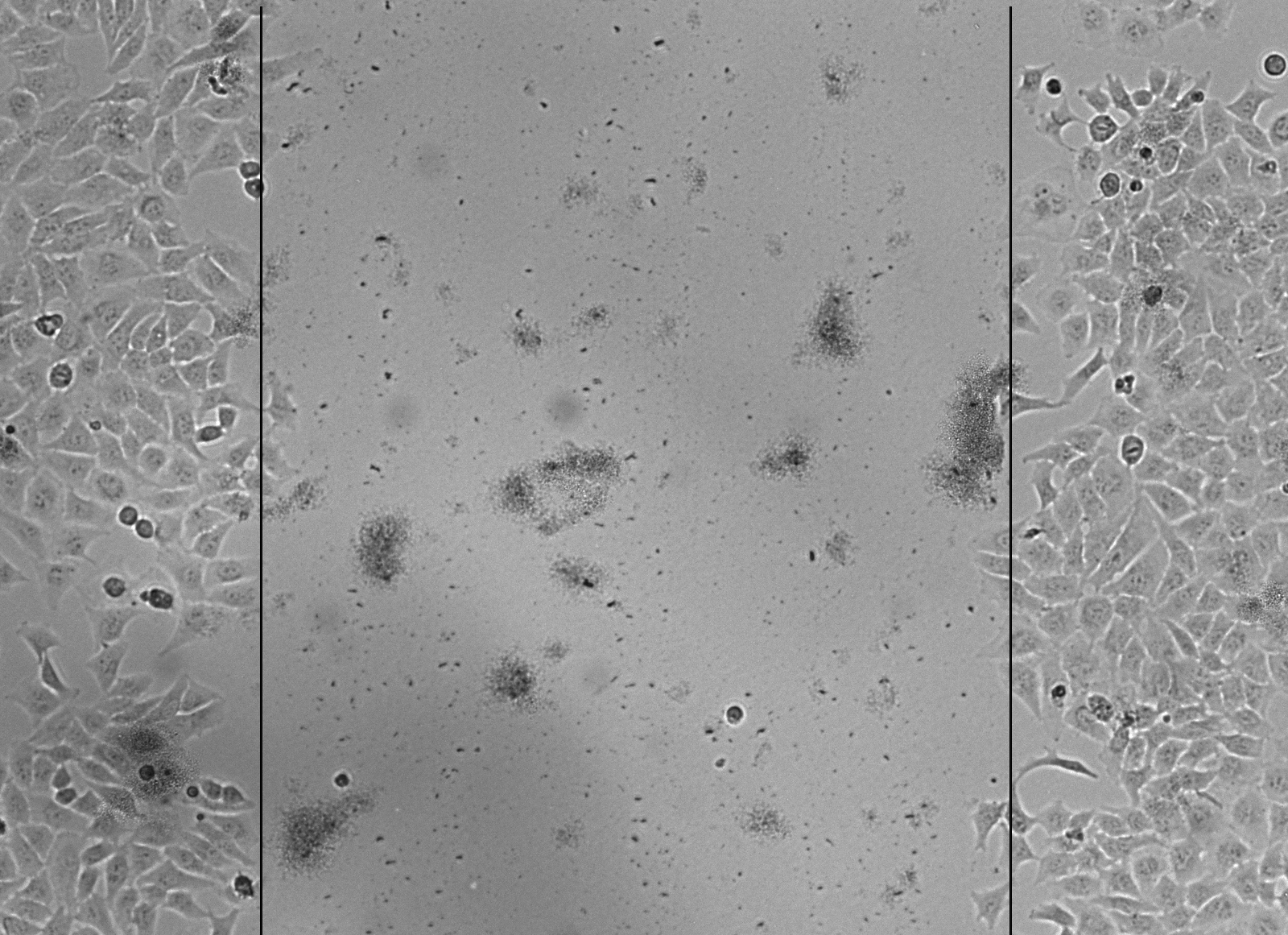

Supplement: Supplementary file 8 — Source data Fig. 6 [file 44319_2025_661_MOESM8_ESM.zip › Figure 6/Figure 6G/sh-MCT2+Tuc-24h.png]

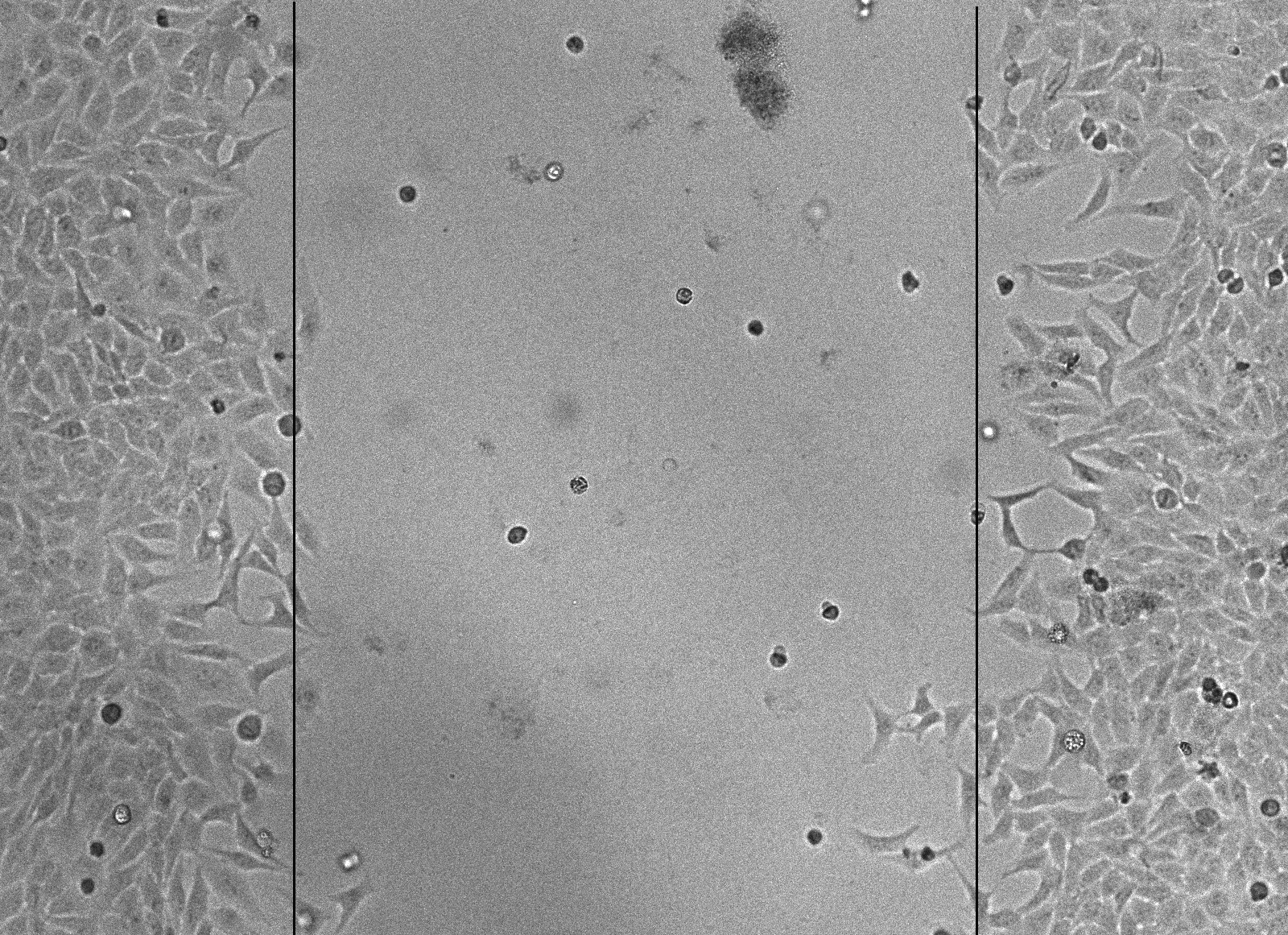

Supplement: Supplementary file 8 — Source data Fig. 6 [file 44319_2025_661_MOESM8_ESM.zip › Figure 6/Figure 6G/sh-MCT2+Tuc-48h.png]

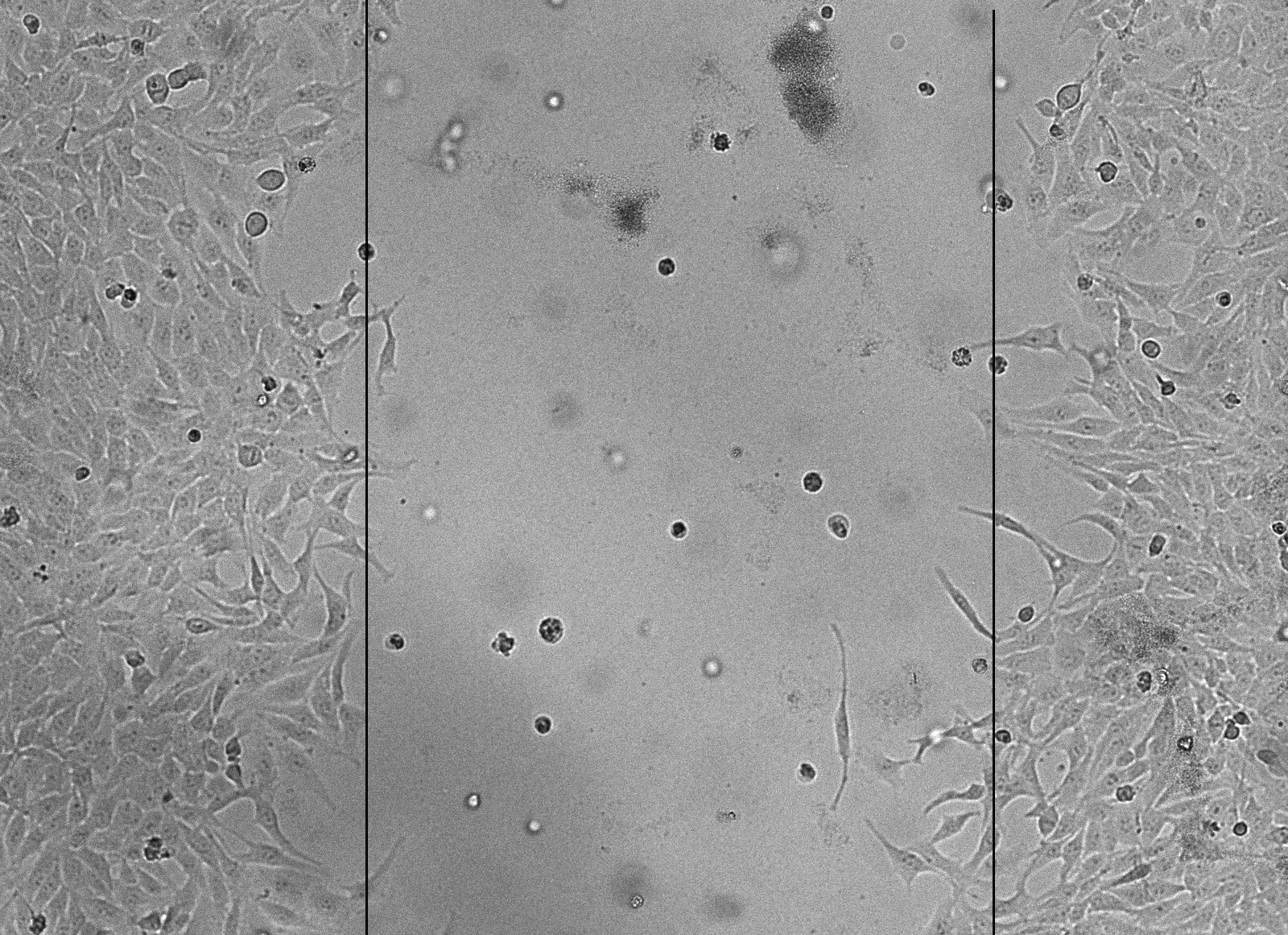

Supplement: Supplementary file 8 — Source data Fig. 6 [file 44319_2025_661_MOESM8_ESM.zip › Figure 6/Figure 6G/sh-MCT2+Tuc-72h.png]

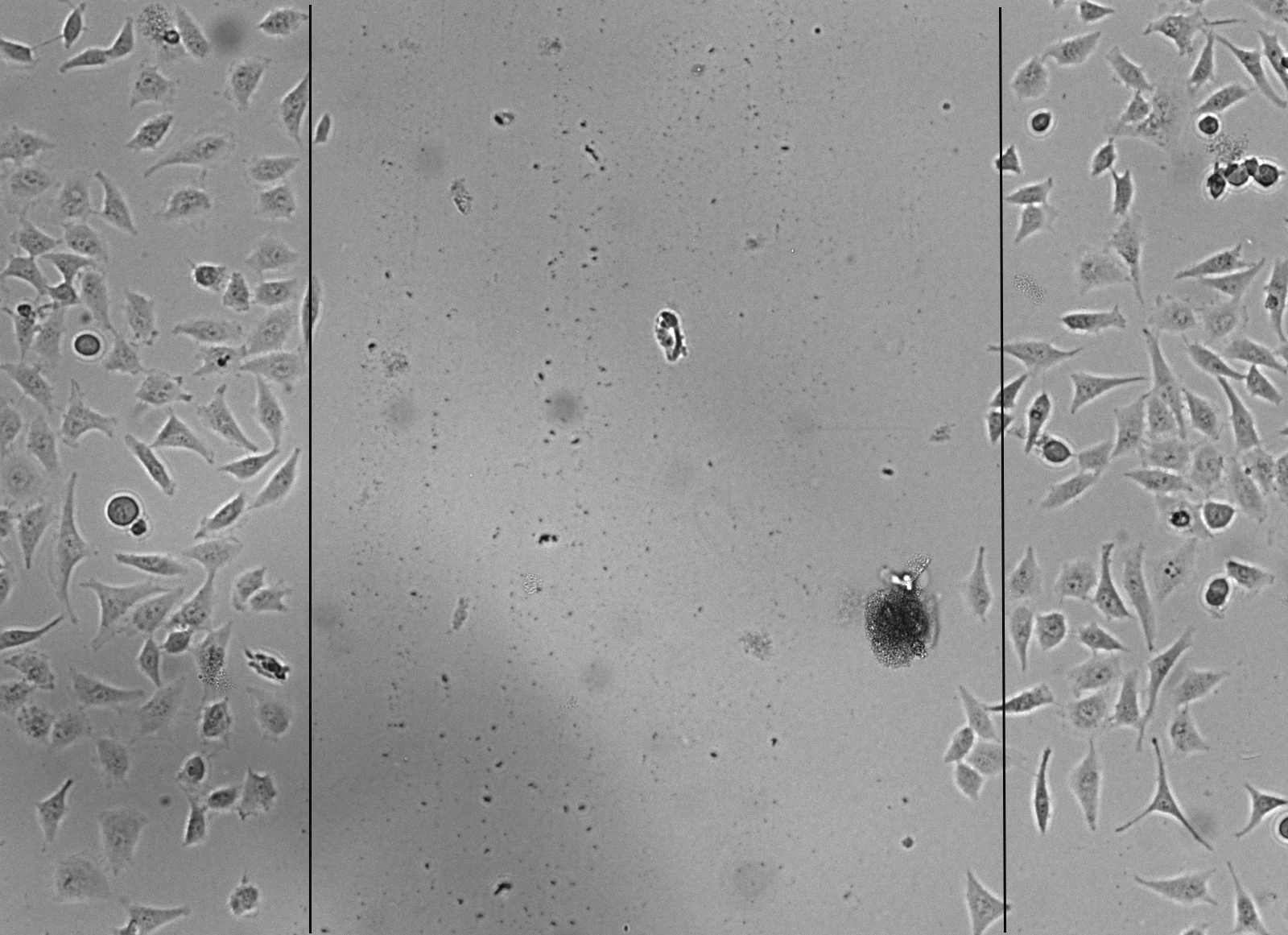

Supplement: Supplementary file 8 — Source data Fig. 6 [file 44319_2025_661_MOESM8_ESM.zip › Figure 6/Figure 6G/sh-MCT2-0h.png]

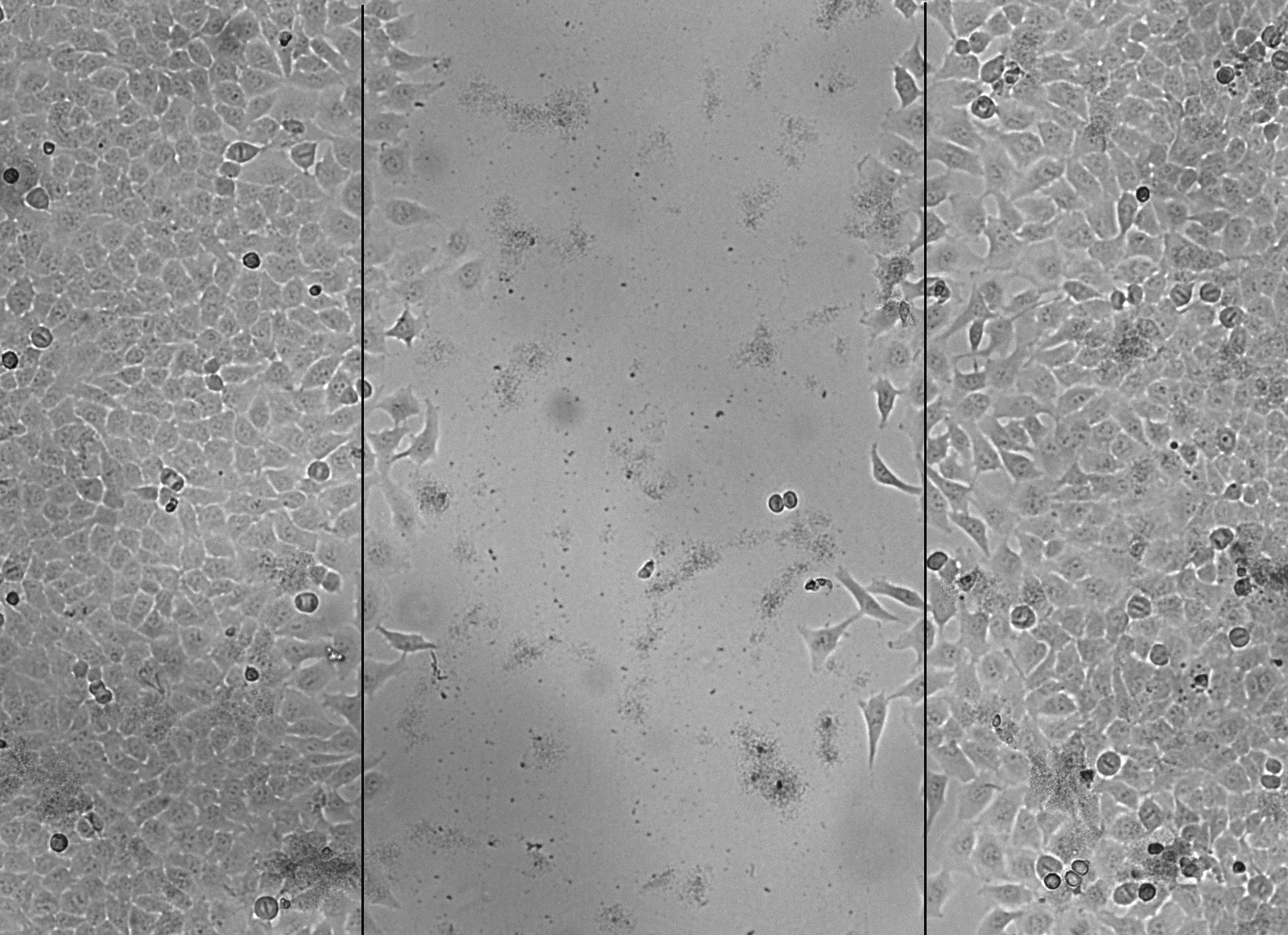

Supplement: Supplementary file 8 — Source data Fig. 6 [file 44319_2025_661_MOESM8_ESM.zip › Figure 6/Figure 6G/sh-MCT2-24h.png]

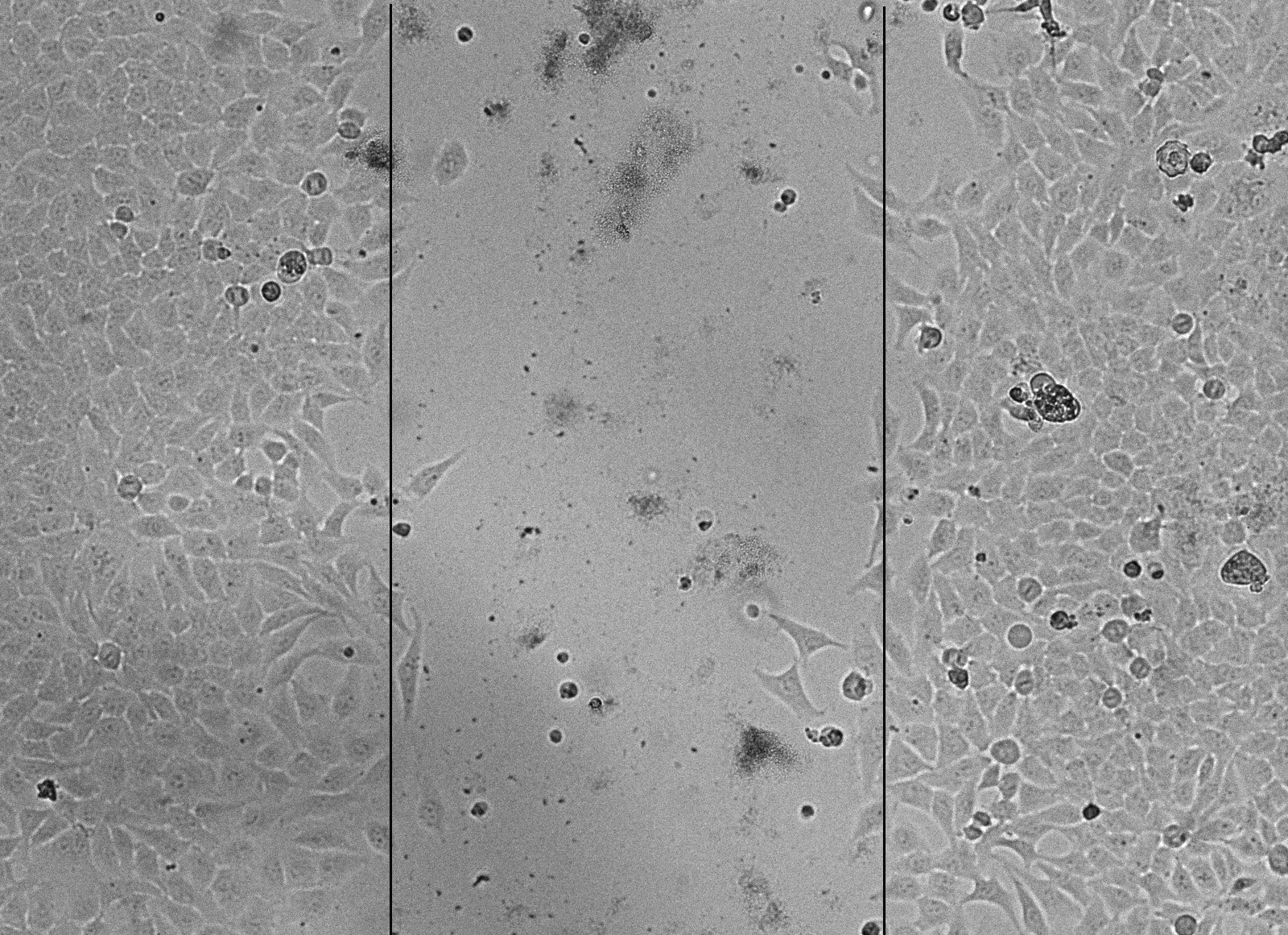

Supplement: Supplementary file 8 — Source data Fig. 6 [file 44319_2025_661_MOESM8_ESM.zip › Figure 6/Figure 6G/sh-MCT2-48h.png]

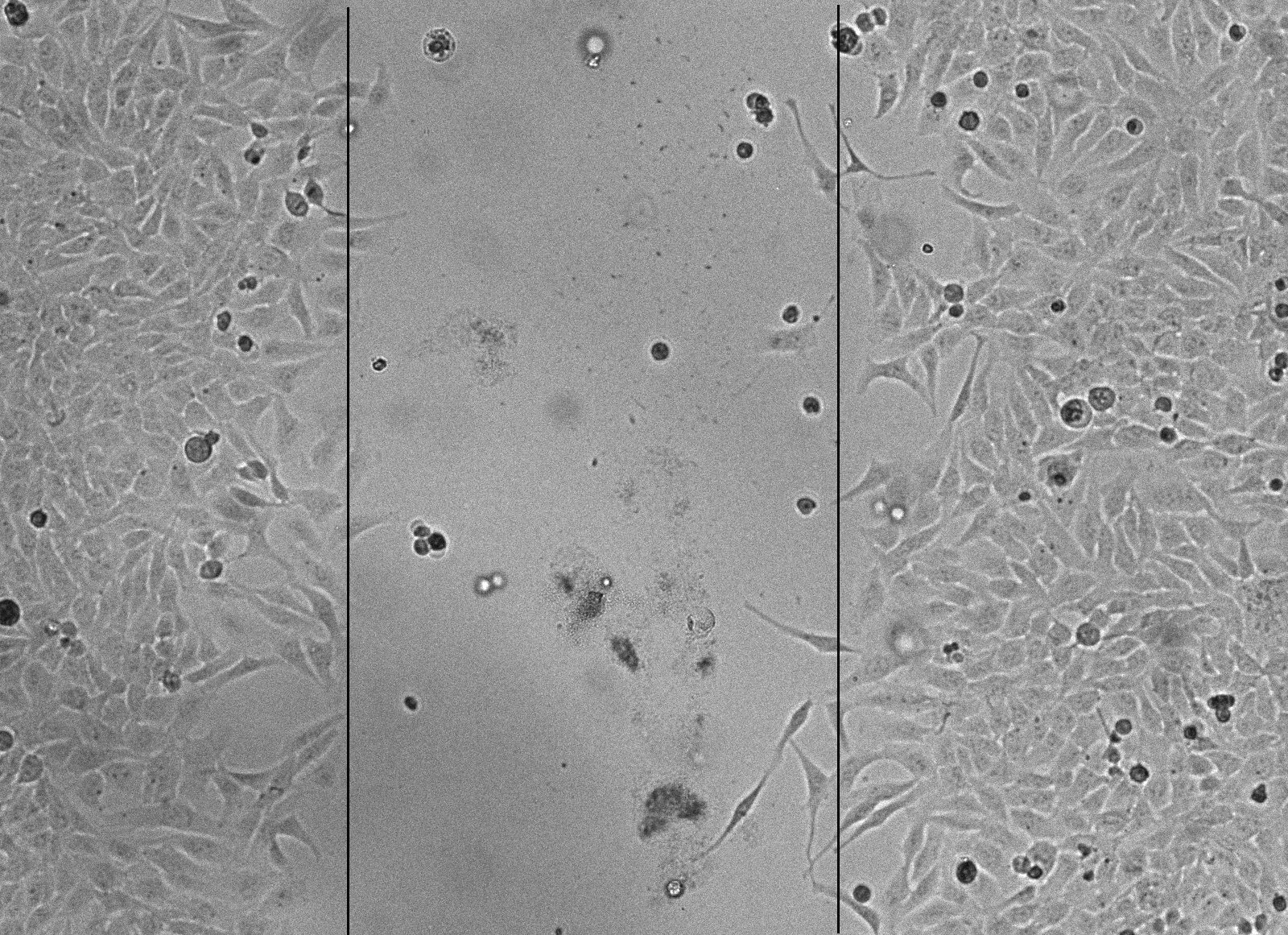

Supplement: Supplementary file 8 — Source data Fig. 6 [file 44319_2025_661_MOESM8_ESM.zip › Figure 6/Figure 6G/sh-MCT2-72h.png]

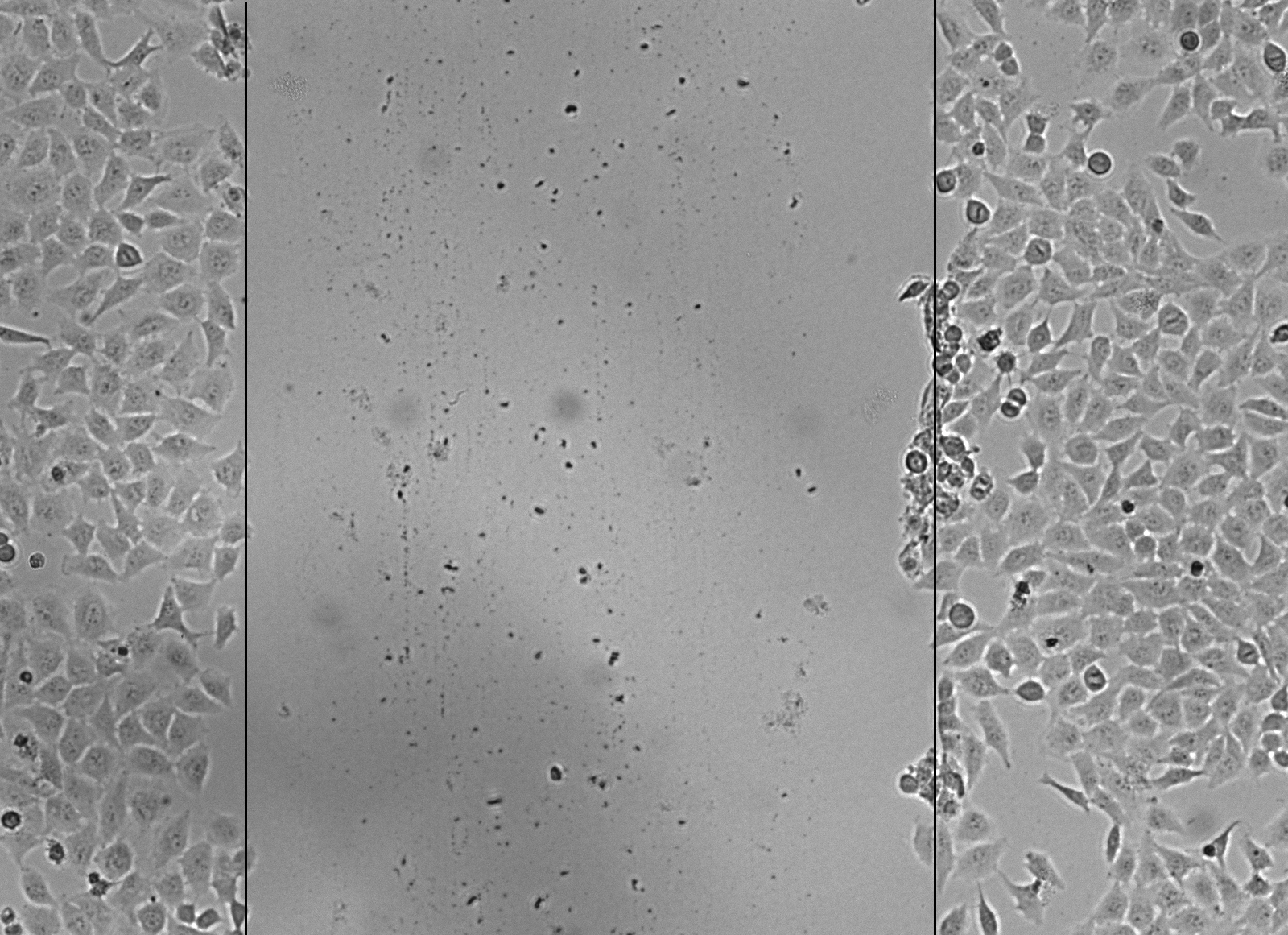

Supplement: Supplementary file 8 — Source data Fig. 6 [file 44319_2025_661_MOESM8_ESM.zip › Figure 6/Figure 6G/sh-NC+Tuc-0h.png]

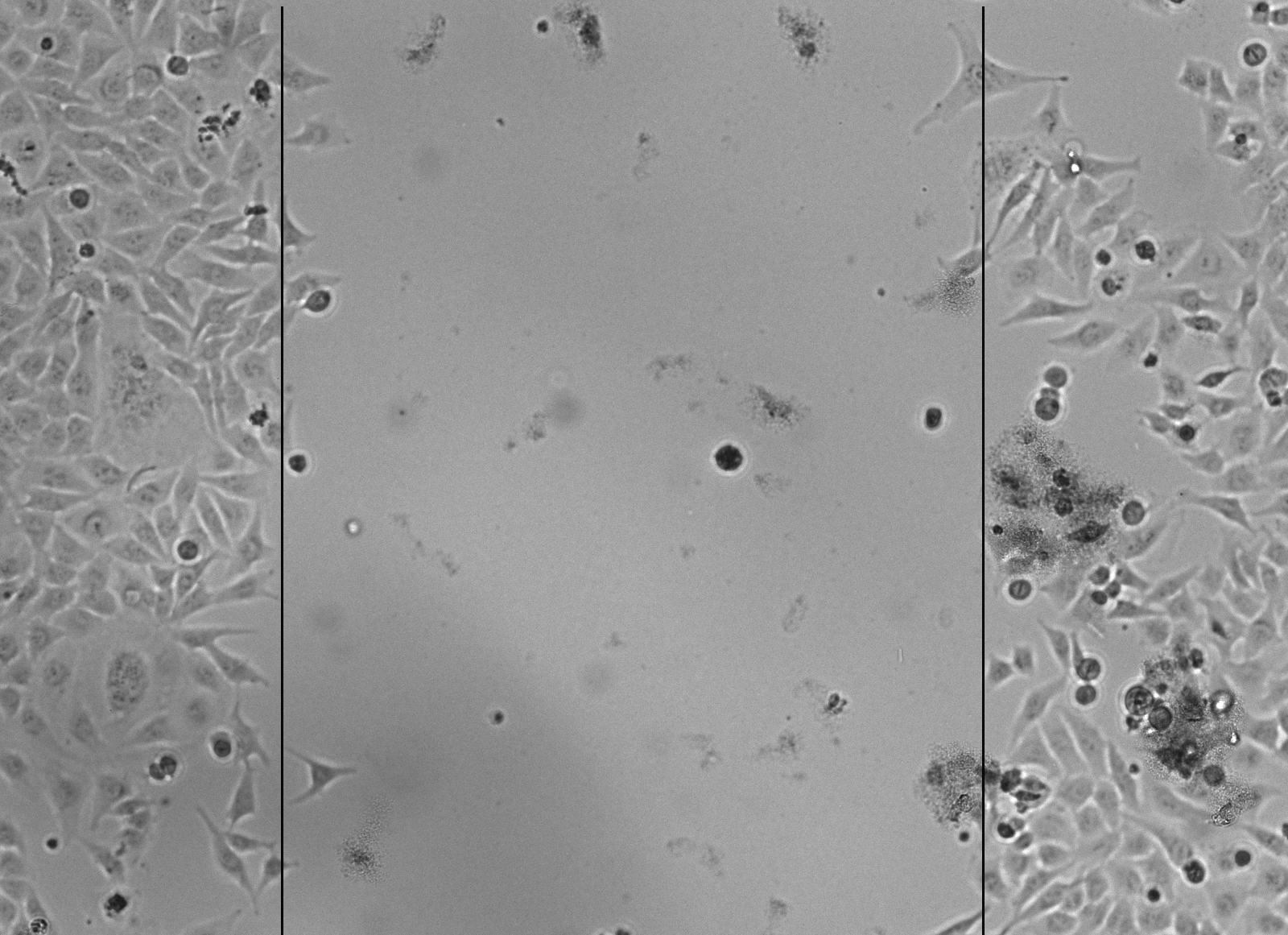

Supplement: Supplementary file 8 — Source data Fig. 6 [file 44319_2025_661_MOESM8_ESM.zip › Figure 6/Figure 6G/sh-NC+Tuc-24h.png]

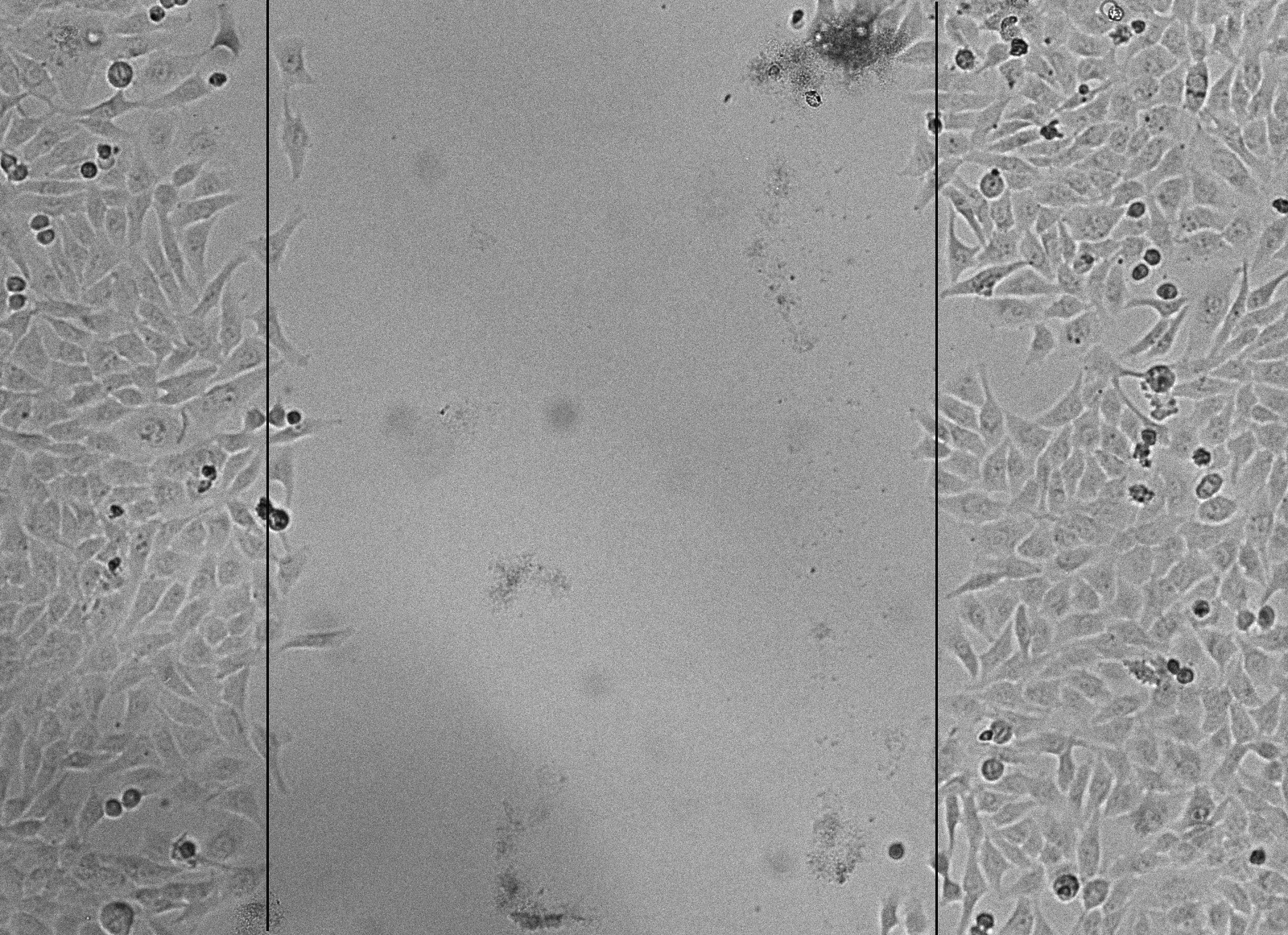

Supplement: Supplementary file 8 — Source data Fig. 6 [file 44319_2025_661_MOESM8_ESM.zip › Figure 6/Figure 6G/sh-NC+Tuc-48h.png]

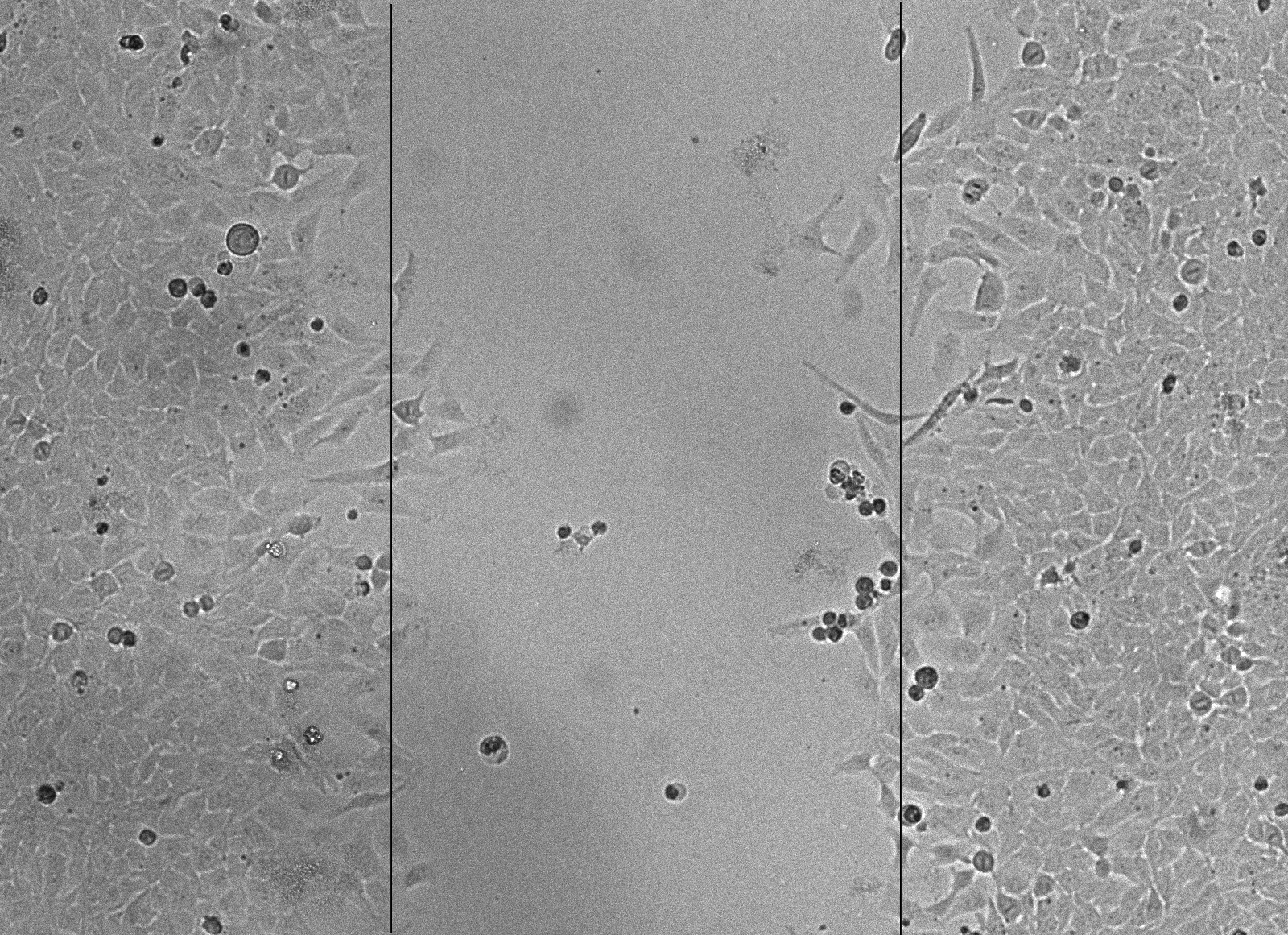

Supplement: Supplementary file 8 — Source data Fig. 6 [file 44319_2025_661_MOESM8_ESM.zip › Figure 6/Figure 6G/sh-NC+Tuc-72h.png]

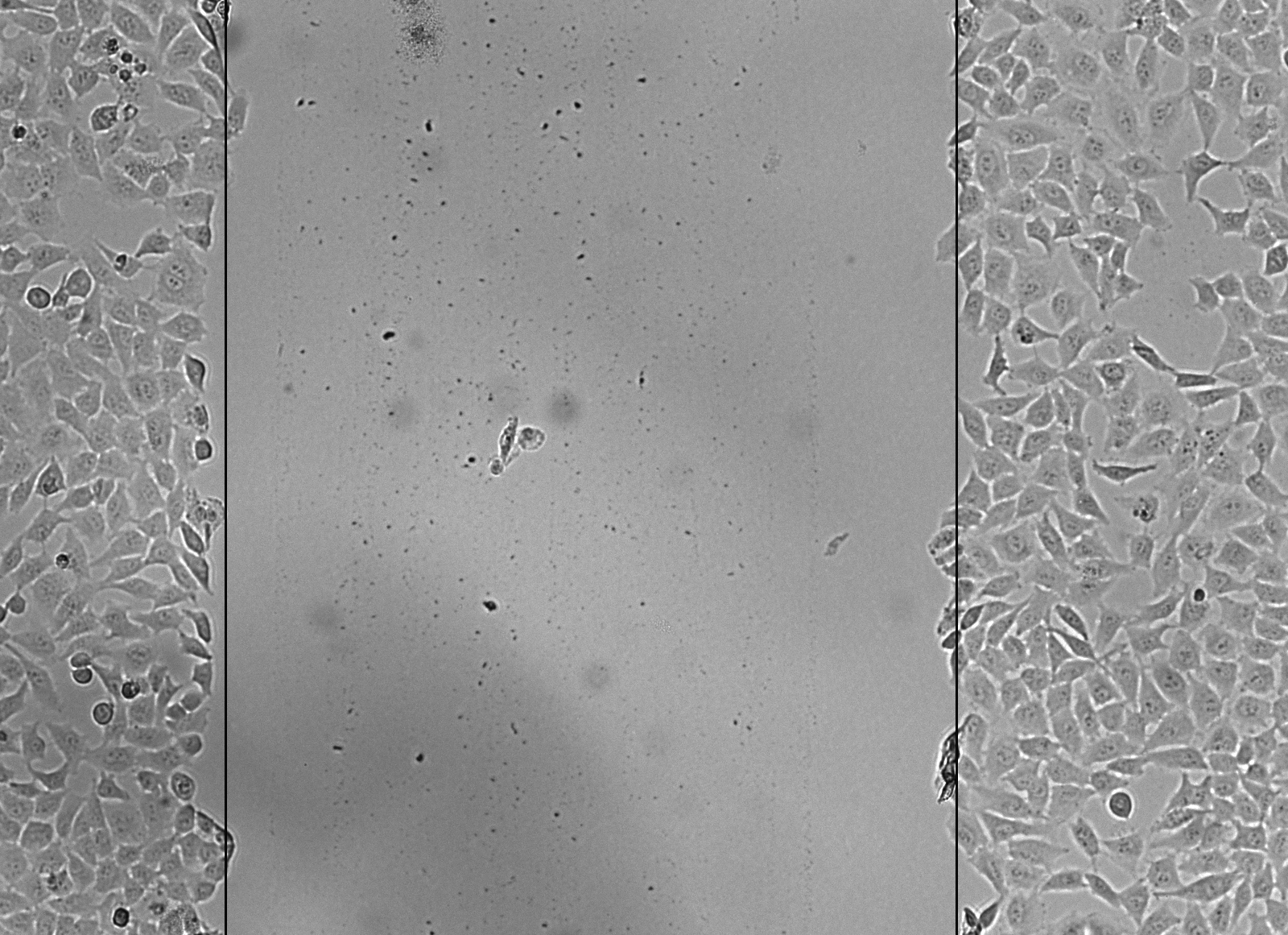

Supplement: Supplementary file 8 — Source data Fig. 6 [file 44319_2025_661_MOESM8_ESM.zip › Figure 6/Figure 6G/sh-NC-0h.png]

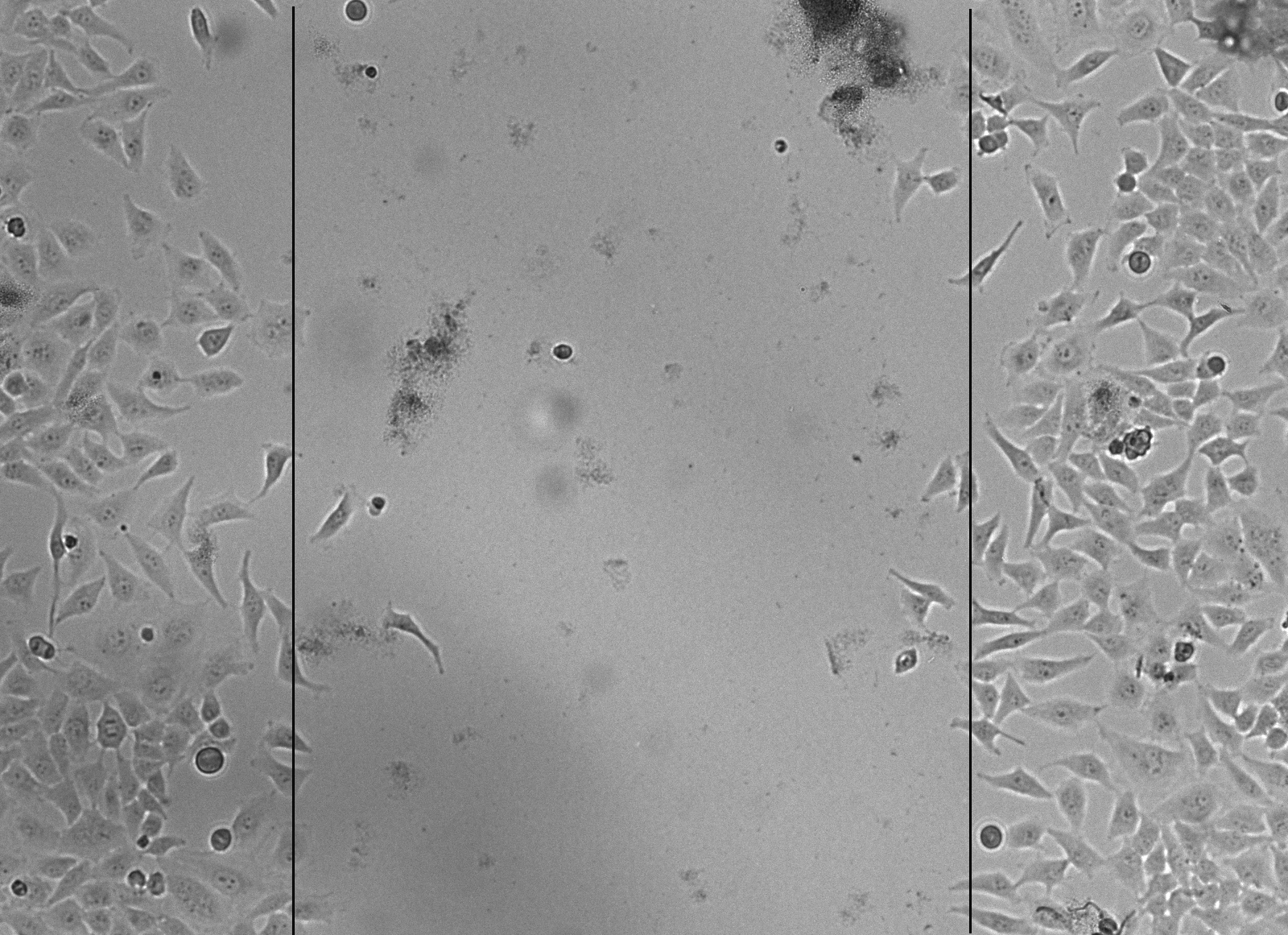

Supplement: Supplementary file 8 — Source data Fig. 6 [file 44319_2025_661_MOESM8_ESM.zip › Figure 6/Figure 6G/sh-NC-24h.png]

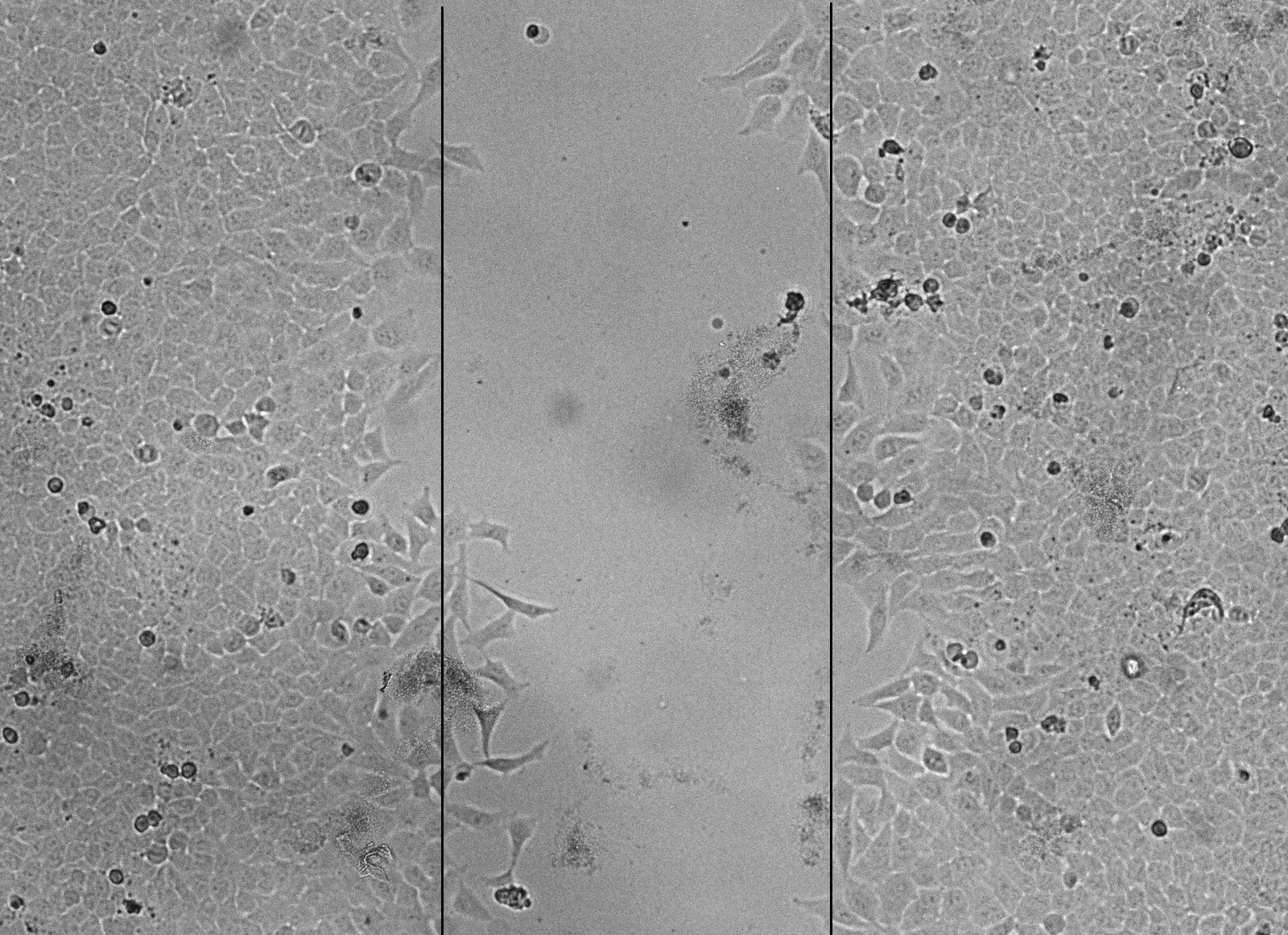

Supplement: Supplementary file 8 — Source data Fig. 6 [file 44319_2025_661_MOESM8_ESM.zip › Figure 6/Figure 6G/sh-NC-48h.png]

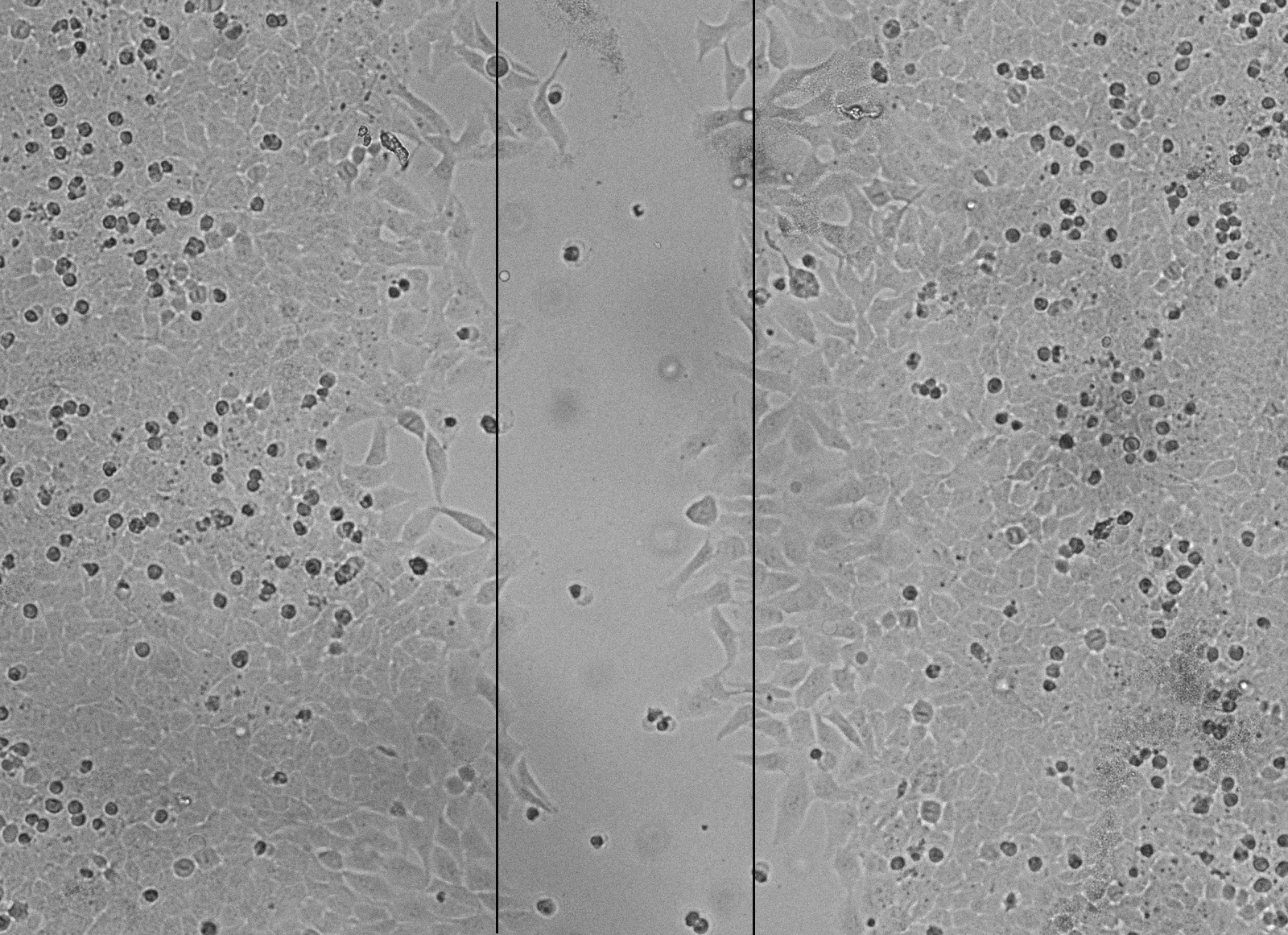

Supplement: Supplementary file 8 — Source data Fig. 6 [file 44319_2025_661_MOESM8_ESM.zip › Figure 6/Figure 6G/sh-NC-72h.png]
