# Supplementary material for: Structure-guided screening identifies Tucatinib as dual inhibitor for MCT1/2
Source: EMBO Rep. 2025 Dec 11;27(3):677–703. doi: 10.1038/s44319-025-00661-9 (PMC12894981; doi:10.1038/s44319-025-00661-9)
Supplement: Supplementary file 12 — Expanded View Figures [file 44319_2025_661_MOESM12_ESM.pdf]

Expanded View Figures

Figure EV1. Structural comparison between human MCT2-embigin and MCT1-embigin, MCT2, respectively, related to Fig. 1.

(**A**) Structural comparison of outward-open human MCT2-emb (PDB ID: 9L0B) and inward-open MCT1-emb (PDB ID: 7YR5). The NTD and CTD remain relatively unchanged during the alternating-access cycle. Structures of MCT2-emb and MCT1-emb are separately aligned relative to NTD or CTD. (**B**) Rotation of TM of representative TM segments between MCT2-emb and MCT1-emb relative to the membrane norm. (**C**) Structural alignments between MCT2-emb (blue, pink, and green) and MCT1-emb (gray). The N-domain was used as a reference for structural alignments. The two structures are superimposed with an RMSD of 1.04 Å over 184 aligned Cα atoms. Black arrows indicate the oscillations of the C-domain. Top: Lumen side view; Bottom: Cytoplasm side view. (**D**) Structure of the human MCT2. The cryo-EM structure of inward-open MCT2 (PDB code: 7BP3) viewed parallel to the plasma membrane (left), extracellularly (right). (**E**) Structural comparison of outward-open human MCT2-emb and one subunit of MCT2, which are separately aligned relative to NTD or CTD. (**F**) Structures of MCT2-emb and MCT2 are aligned to NTD from the intracellular side. TM regions of MCT2 are displayed as a cylinder. (**G**) Conformational changes of TM5 between human MCT2-emb and MCT2. TM5 is a relatively straight helix in MCT2-emb, but the cytosolic half of TM5 in MCT2 swings toward the center of the transporter by about 30 degrees.

© The Author(s)

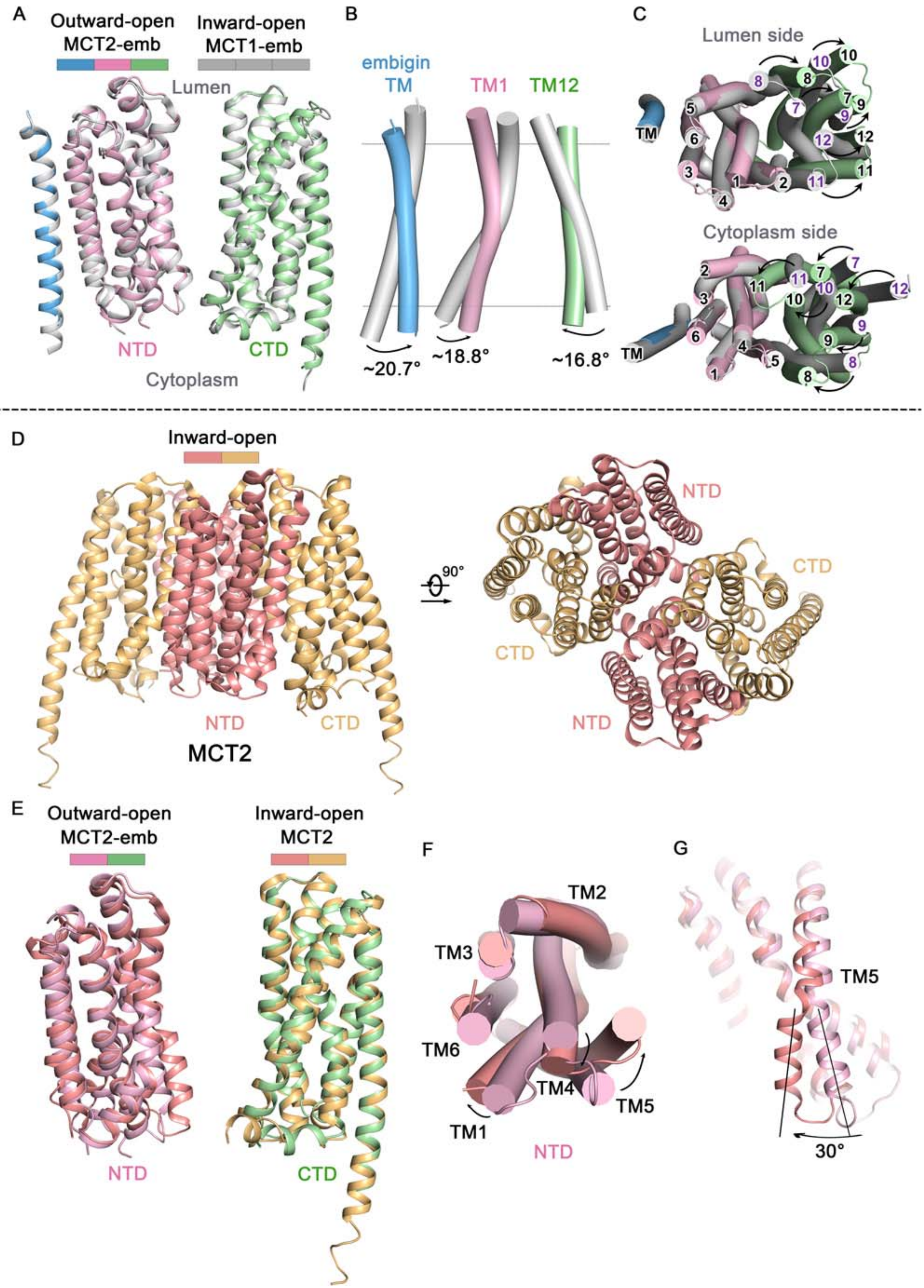
A
Outward-open
MCT2-emb
Inward-open
MCT1-emb
Lumen
NTD
CTD
Cytoplasm
B
embigin
TM
TM1
TM12
~20.7°
~18.8°
~16.8°
C
Lumen side
Cytoplasm side
TM
D
Inward-open
NTD
CTD
MCT2
90°
E
Outward-open
MCT2-emb
Inward-open
MCT2
NTD
CTD
F
TM2
TM3
TM6
TM4
TM5
TM1
NTD
G
TM5
30°

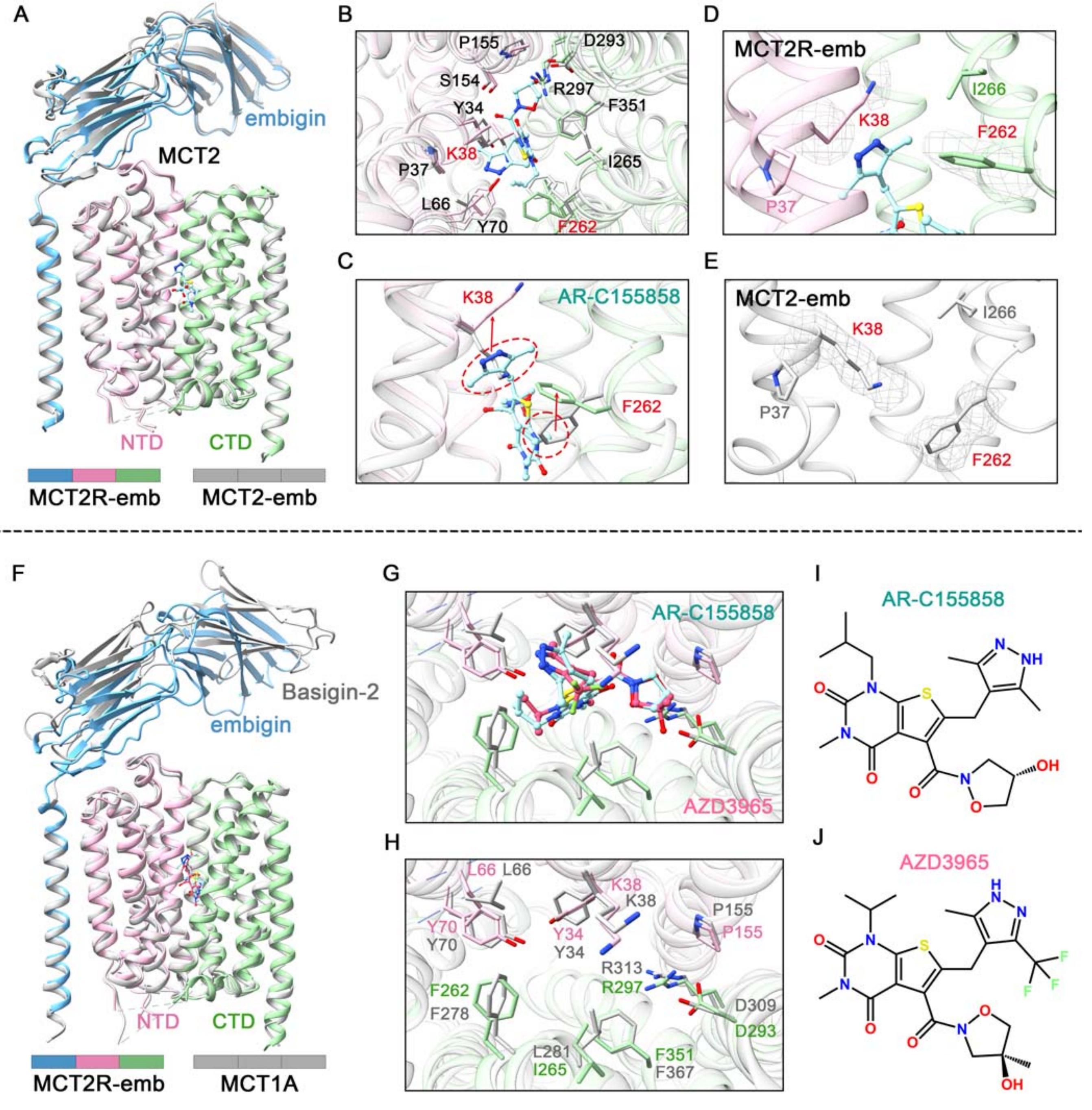

Figure EV2. Structural comparison between human MCT2R-emb and MCT2-emb, MCT1A, respectively, related to Fig. 2.

(**A**) MCT2-embigin exhibits identical conformation in the presence of AR-C155858. The two structures can be superimposed with an RMSD of 0.67 Å over 387 aligned Cα atoms. (**B**) Comparison of residues involved in the coordination of AR-C155858. AR-C155858 are shown as ball and sticks, and the residues are shown as sticks. (**C**) Conformational shift of K38 on TM1 and F262 on TM7 upon AR-C155858 binding. The red dashed circles indicate potential clashes should these residues exhibit the same conformation as in the apo state. (**D**, **E**) The cryo-EM density of K38 and F262 in MCT2R-emb and MCT2-emb, respectively. (**F**) Structural comparison of outward-open human MCT2R-emb (PDB ID: 9L0C) and outward-open MCT1A (PDB ID: 6LYY). The two structures can be superimposed with an RMSD of 0.85 Å over 367 aligned Cα atoms. (**G**, **H**) Comparison of residues involved in the coordination of AR-C155858 on MCT2R-emb or AZD3965 on MCT1A. AR-C155858 and AZD3965 are shown as cyan and magenta, respectively. The interacting residues are shown as sticks. The ligands are omitted for visual clarity in (**H**). Chemical structural formula of AR-C155858 (**I**), AZD3965 (**J**).

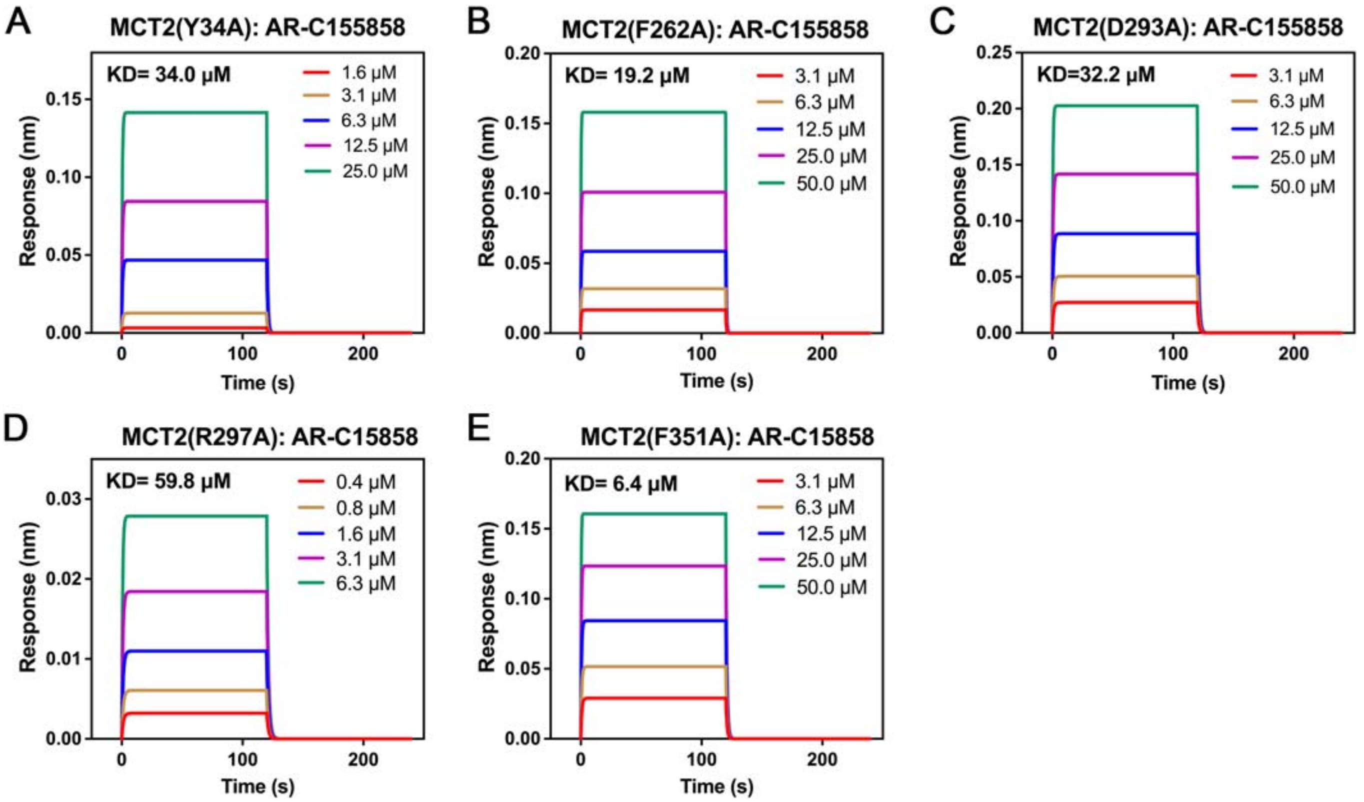

Figure EV3. Binding affinities between MCT2 variants and AR-C155858, related to Fig. 2.

(**A–E**) Bio-layer interferometry (BLI) measurement of the binding affinities between MCT2 variants and AR-C155858. The binding kinetics of AR-C155858 bound to MCT2(Y34A)-embigin (**A**), MCT2(F262A)-embigin (**B**), MCT2(D293A)-embigin (**C**), MCT2(R297A)-embigin (**D**), MCT2(F351A)-embigin (**E**). Source data are available online for this figure

 © The Author(s)

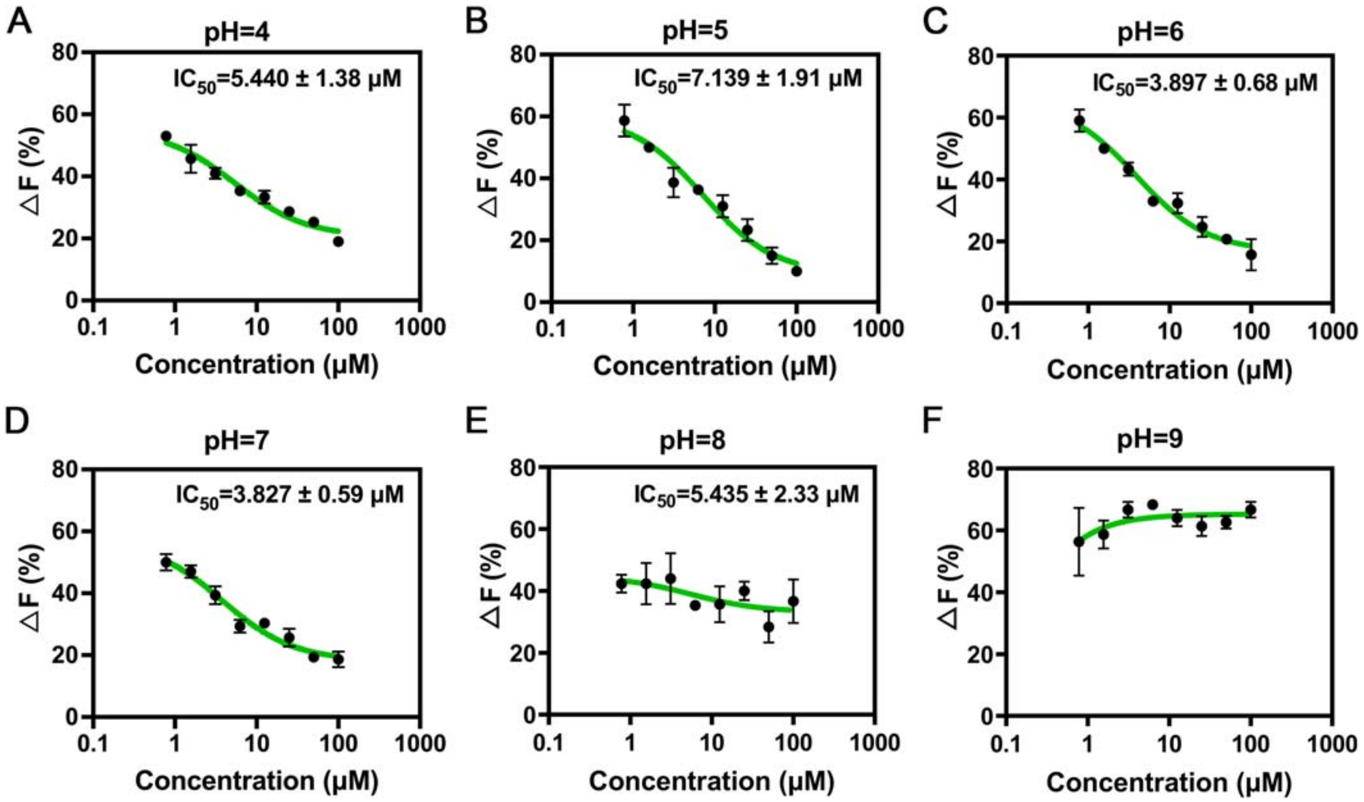

Figure EV4. pH-dependent inhibition of MCT2 by Tucatinib, related to Fig. 3.

The half maximal inhibitory concentration (IC_{50}) of Tucatinib on MCT2 across pH gradients, pH = 4 (**A**), pH = 5 (**B**), pH = 6 (**C**), pH = 7 (**D**), pH = 8 (**E**), pH = 9 (**F**). ($n = 3$). The graphical presentation and data analysis were conducted using GraphPad Prism 9. The data were displayed as mean ± standard deviation (SD). $n = 3$ biological replicates. Source data are available online for this figure
